# Sustained high prevalence of viral hepatitis and sexually transmissible infections among female sex workers in China: a systematic review and meta-analysis

**DOI:** 10.1186/s12879-015-1322-0

**Published:** 2016-01-05

**Authors:** Shu Su, Eric P. F. Chow, Kathryn E. Muessig, Lei Yuan, Joseph D. Tucker, Xiaohu Zhang, Jiehui Ren, Christopher K. Fairley, Jun Jing, Lei Zhang

**Affiliations:** 1 School of Public Health and Preventive Medicine, Faculty of Medicine, Nursing and Health Sciences, Monash University, Melbourne, VIC Australia; 2 Melbourne Sexual Health Centre, Alfred Health, Melbourne, Australia; 3 Central Clinical School, Faculty of Medicine, Nursing and Health Sciences, Monash University, Melbourne, VIC Australia; 4 The University of North Carolina Project-China, Guangzhou, China; 5 Department of Health Behaviour, Gillings School of Global Public Health at the University of North Carolina at Chapel Hill, Chapel Hill, USA; 6 The School of Sociology and Population Studies, Renmin University of China, Beijing, China; 7 London School of Hygiene and Tropical Medicine, London, UK; 8 China Food and Drug Administration Institute of Executive Development, Beijing, China; 9 Research Center for Public Health, School of Medicine, Tsinghua University, Beijing, China; 10 The School of Sociology, Tsinghua University, Beijing, China

**Keywords:** Female sex worker, Sexually transmitted infection, Hepatitis, China, meta-analysis

## Abstract

**Background:**

The 1980’s economic boom has been associated with a rapid expansion of China’s sex industry over the past three decades. Consequently, the spread of sexually transmitted infections (STIs) and hepatitis infections among female sex workers (FSW) has become an important public health issue in China. This study identifies prevalence and risks of hepatitis and STIs in Chinese FSWs.

**Method:**

Four electronic databases were searched for Chinese and English language peer-reviewed studies conducted between 01/2000-12/2011 that reported prevalence of hepatitis and STIs (excluding HIV) among Chinese FSW. Following the PRISMA guidelines, meta-analysis was used to estimate pooled prevalence and 95 % confidence intervals for each infection.

**Result:**

Three hundred and thirty nine articles (34 in English and 305 in Chinese) investigating 603,647 FSWs in 29 Chinese provinces were included in this review. Over the period 2000–2011, the seroprevalence of active hepatitis B and hepatitis C among FSW were 10.7 % (7.3–15.5 %) and 1.0 % (0.7–1.3 %), respectively. The most prevalent STI was human papillomavirus (HPV, 27.0 % [10.1–55.1 %]), followed by herpes simplex virus-2 (HSV-2, 15.8 % [11.7–20.9 %]), chlamydia (13.7 % [12.1–15.4 %]), gonorrhoea (6.1 % [5.3–7.0 %]), syphilis (5.2 % [4.8–5.7 %]), genital warts (3.3 % [2.5–4.2 %]) and *Trichomonas vaginitis* (2.1 % [1.5–24.2 %]). Disease burden of both hepatitis and STI among FSW were concentrated in South Central and Southwest China. In particular, chlamydia and syphilis demonstrated a significant declining trend during the studied period (P < 0.05). Compared with the general Chinese population, FSW had significantly higher prevalence of all STIs except *Trichomonas vaginitis*. Further, compared to the general FSW population, HIV-positive FSW had significantly higher prevalence of syphilis, chlamydia, HSV-2 and *Trichomonas vaginitis*.

**Conclusion:**

Prevalence of hepatitis and STIs remained high and mostly stable among Chinese FSW over the period of 2000–2011. Targeted STI and hepatitis surveillance and interventions should be strengthened among Chinese FSWs, especially those who are HIV-positive.

**Electronic supplementary material:**

The online version of this article (doi:10.1186/s12879-015-1322-0) contains supplementary material, which is available to authorized users.

## Background

Every year, sexually transmitted infections (STIs) affect more than 500 million people worldwide, posing a substantial threat to sexual and reproductive health of the world population [1]. While most STIs are curable with timely diagnosis and treatment, others lead to serious long-term effects including reproductive complications and death. Furthermore, individuals infected with STIs are more likely to contract or transmit HIV, and co-infection with hepatitis can complicate the treatment for HIV [2–4]. In response to these concerns, the World Health Organization has recommended prevention of STIs and hepatitis as a reproductive health priority [5, 6].

The 2013 statistics from the Chinese Ministry of Health reported a rapidly rising trend in STIs since the 1979 economic reform [7, 8]. In the contexts of increasingly open attitudes toward commercial sex and higher disposable incomes, soliciting female sex workers (FSW) is increasing in China [9, 10]. Commercial sex is illegal in China and subjected to harsh publishment, FSWs who are caught are sent to detention centres. Most Chinese FSWs sell sex in concealed venues and have established their own network to avoid being detected by the police. Given their opaque characters, frequent risk sexual activities and low awareness, Chinese FSWs are regarded as a highly at-risk group for STIs [11, 12]. Hepatitis C and B are also of concern among this group mainly due to higher rates of reported drug use (for HCV) and unsafe sexual behaviours (for HBV) among FSW as compared to the general population [13, 14]. Furthermore, transmission of STIs and hepatitis through male clients of FSW may have potential adverse impacts on the broader female population [15]. The estimated prevalence of HIV, syphilis, chlamydia and gonorrhoea were all higher among clients of female sex workers than among the general population, and a systematic review conducted in 2000–2012 demonstrated an significant increase in syphilis among male clients [16].

China’s STI surveillance system routinely collects epidemic data of STIs only on syphilis and gonorrhoea. The data is collected by China CDC, but most of the data remain unpublished. Although numerous independent studies and an earlier systematic review [17] have reported STI and hepatitis infection burden among FSWs across China, these data have not been integrated to inform the overall geographic and temporal trends of STI infections among FSWs. In this study, we conducted a comprehensive data synthesis of hepatitis and STI epidemic information to inform the current disease burden, temporal trends and geographic distribution of STI infections among FSWs in China. This knowledge is important for informing HIV/STI health policies and designing evidence-based strategies for hepatitis and STI prevention among FSWs in China.

## Methods

This meta-analysis was performed according to the PRISMA (Preferred Reporting Items for Systematic Reviews and Meta-Analyses) Statement issued in 2009 (Checklist S1).

### Literature and search strategy

We searched one English database (PubMed) and three Chinese databases (Wanfang Data, VIP Chinese Journal Database and China National Knowledge Infra-structure) for studies conducted between January 1, 2000 to December 31, 2011 that reported the prevalence of STIs or hepatitis infections among FSW in mainland China.

The search included Medical Subject Headings (MeSH) terms for ‘China’, ‘Chinese’, ‘CSW (commercial sex workers)’, ‘FSW’, ‘hepatitis’, ‘sexually transmitted diseases’ and ‘sexually transmitted infections’, and other keywords associated with each STI: ‘chlamydia’, ‘*Chlamydia trachomatis*’, ‘gonorrhoea’, ‘*Neisseria gonorrhoea*’, ‘syphilis’, ‘genital warts’, ‘hepatitis’, ‘HBV’, ‘hepatitis B’, ‘HCV’, ‘hepatitis C’, ‘HSV’, ‘herpes simplex virus’, ‘HPV’, ‘human papillomavirus’ and ‘*trichomonas vaginitis*’. Hand searching from the reference lists of the retrieved articles in the above databases was also included. Only articles published in Chinese or English language were included. Two reviewers (EPFC, XHZ) independently screened all retrieved abstracts from the four aforementioned databases to assess eligibility. Discrepancies were resolved by discussion with a third reviewer (LZ).

### Selection criteria

#### Type of studies

Eligible study designs included quantitative epidemiological studies, including cohort and cross-sectional studies. The following types of publications were excluded: news reports, review articles, conference abstracts, mathematical modelling studies, clinical case studies, dissertations, studies with sample size less than 30 for general FSW or less than 10 for HIV-positive FSW, and STI prevalence reported without authenticated diagnosis (syphilis and hepatitis prevalence without serological test). Studies not conducted in mainland China were excluded.

#### Type of participants

We only included studies with FSW who self-reported having any commercial sexual activity (sell sex for money) in the past 12 months. There were no restrictions on age, marital status, educational level, ethnicity or residency. Studies that targeted HIV-positive FSW were included to investigate the prevalence of co-infection.

#### Type of outcome measures

We included studies that measured the prevalence of the most common STIs among FSW including syphilis, chlamydia, gonorrhoea, HPV/genital warts, HSV-2, and *Trichomonas vaginitis*. We also included HBV infection which is sexually transmitted and HCV infection which can be transmitted through injecting drug use. Studies were included if syphilis, chlamydia, gonorrhoea, HPV/genital warts, HSV-2, *Trichomonas vaginitis,* HBV or HCV was diagnosed and confirmed by a serological testing approach. We included published studies in which HBV infection was defined by the presence of hepatitis B surface antigen (HBsAg) as HBsAg is a serologic marker representing either acute or chronic HBV infection rather than due to other cases such as HBV immunity; HCV infection was diagnosed by the presence of anti-HCV antibodies; HPV infection was tested by PCR for any of the genital HPV types; chlamydia, gonorrhoea were tested by PCR, EIA or cell culture; syphilis was tested by specific treponemal serological method, rapid plasma regain or PCR and any positive result was considered positive; *Trichomonas vaginitis* was tested by wet mount and PCR; genital warts were clinically diagnosed based on the presence of warts; HSV-2 was diagnosed by the presence of type specific Immunoglobulin G (IgG) by EIA. Self-reported infections were excluded from this review.

### Quality assessment

The authors assessed the methodological quality of every included study using an 8-item checklist tool for observational studies [18]. Based on the checklist guidelines, the quality of each item was categorized as either “High quality” (1 point) or “Low quality” (0 points) for a total possible score ranging between 0 and 8. Studies with higher scores were regarded as higher quality.

### Data extraction

Data were exported and entered into a Microsoft Excel database (Version 2010, Microsoft Corp., Redmond, WA, USA). Each study was given a unique ID number and the data collection form included information on: (1) Study design: location, sampling methods/venues, type of study, sample size, and study year; (2) epidemiology of STIs and hepatitis infections: prevalence estimates of STIs, HBV or HCV, prevalence estimates of HPV gene type subgroups and prevalence estimates of sites and biomarkers of chlamydia and gonorrhoea.

### Statistical analysis

Pooled estimates with 95 % confidence intervals (*CI*) were calculated for the prevalence of each STI and hepatitis infection among Chinese FSW. These estimates were expressed as a percentage (number of infections divided by the number of individuals tested for the infection). Pooled odds ratios *(OR)* with 95 % *CIs* were calculated by comparing the pooled estimates between FSW and the general Chinese adult population. To obtain STI prevalence for various subgroups, we also conducted sensitivity analyses, and stratified by geographic region (North, Northeast, Northwest, East, South Central, Southwest), FSW venue type (entertainment venues, national sentinel sites, detaining education centres, VCT and others), sample size, language of the publication, study quality score, and study year (Additional file 1: Table S1 and S3). To estimate the risk of STI acquisition in HIV-positive FSW, the pooled *OR* with a 95 % *CI* was calculated by comparing the pooled STI prevalence estimates among HIV-positive FSW and general FSW. STI and hepatitis prevalence of the general population was estimated for the Chinese population of reproductive age (i.e. >15 years old) using data from national or large cross-sectional studies [19–24].

**Fig. 1:**
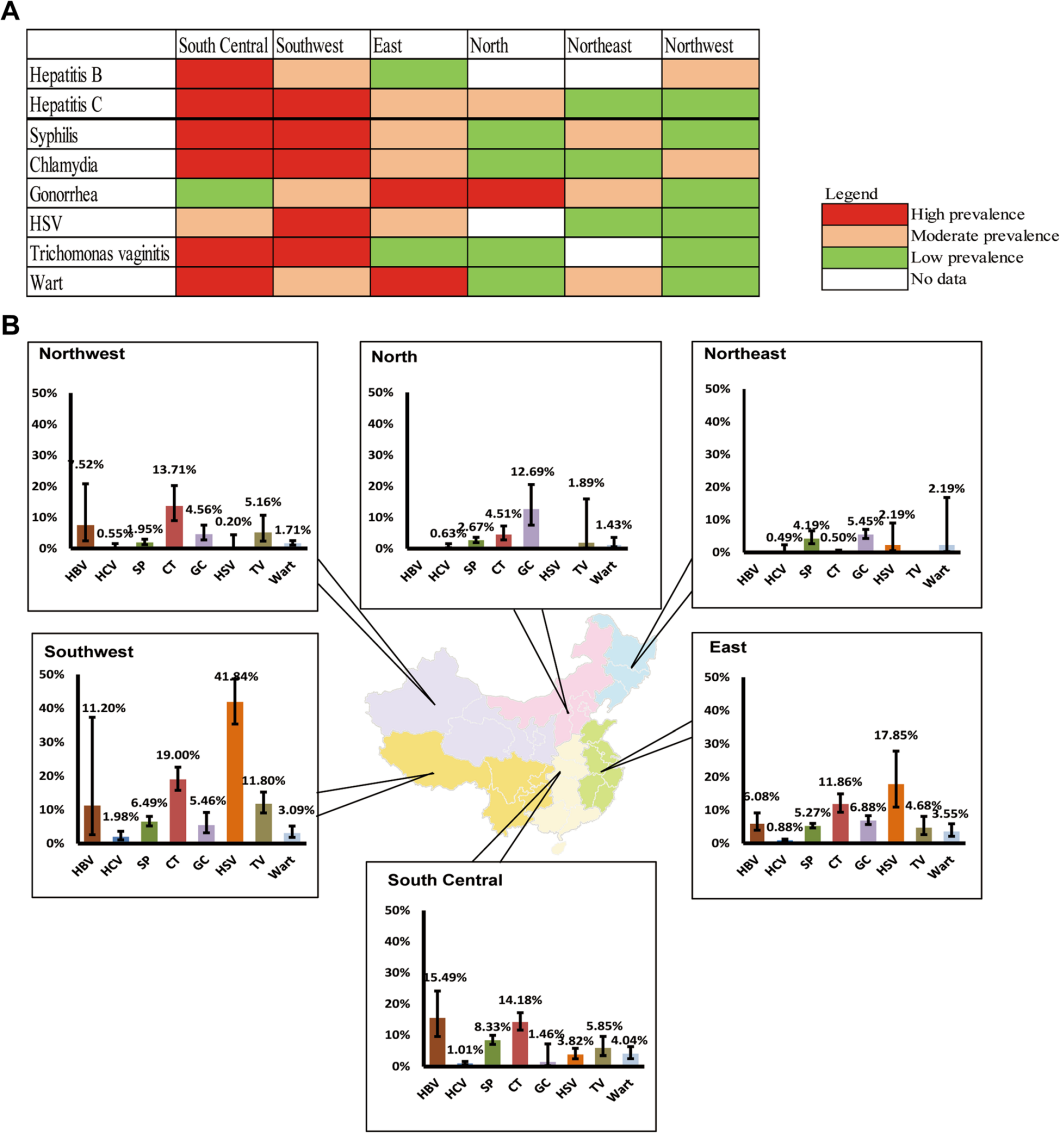
**a**. Estimated prevalence of sexually transmitted infections including Hepatitis B and C virus among female sex workers in six Chinese regions during 2000–2011. *Only infection with > 10 papers were included, so the analysis excluded HPV because there are only four papers being found. **b**. Level of disease burden among female workers across the six regions in China during 2000–2011 (high, moderate and low prevalence represent the top, middle and bottom one-third of prevalence ranking among the six Chinese regions). *Only infection with > 10 papers were included, so the analysis excluded HPV because there are only four papers being found

Heterogeneity across included studies was assessed using a chi-squared based Cochran’s Q test and an *I* ^*2*^ test. If the data showed low heterogeneity (*p* > 0.05, *I* ^*2*^ < 50 %), a fixed-effect model was used; otherwise, a random-effect model was used. Publication bias was tested by the Begg and Mazumdar rank correlation [25]. Geographical regions with first and second highest prevalence were defined as ‘high prevalence’ areas, the third and fourth ones were defined as ‘moderate prevalence’ whereas the fifth and sixth ones were defined as ‘low prevalence’ (Fig. 1b). The temporal trend of STIs was tested by chi-square trend test comparing the study periods 2000–2002, 2003–2005, 2006–2008, and 2009–2011 (Fig. 3). All meta-analyses were performed by using the Comprehensive Meta-Analysis (version 2.2, Biostat, Englewood, New Jersey).

## Results

### Characteristics of the studies

Out of the 3015 studies identified through initial searches, 339 studies (English: 34; Chinese: 305) were eligible and included in this review (Additional file 2). The majority of studies were cross-sectional observational studies (n = 286). Eighteen studies reported on HBV, 97 on HCV, 215 on syphilis, 96 on chlamydia, 116 on gonorrhoea, 38 on HSV-2, 43 on *Trichomonas vaginitis*, 68 on genital warts and 4 on HPV, respectively (Table 1). From these published studies, 603,647 FSWs in 29 Chinese provinces were included in this review. Sample size ranged from 11 to 12,363. The mean age of FSW was 24.2 ± 2.4, the average length of time working in commercial sex was 2.4 years. The estimated percentage of FSW who were married was 35.3 %, and 76.9 % had only completed junior middle school education or below.

**Table 1:** Comparison of sexually transmitted infections prevalence between female sex workers and general female population in China

Type of infection	No. of studies (No. of data points)	Total No of infection/Sample size	Pooled prevalence among FSW (95 % CI)	Background prevalence among general population (95 % CI)	Odds ratio (95 % CI)
HBV	18 (24)	848/7,614	10.71 (7.30–15.48)	6.40 (4.45–9.08)* [26]	1.75 (1.60–1.92)
HCV	97 (149)	1,366/114,722	0.96 (0.73–1.25)	0.12 (0.05–0.27)* [20]	7.99 (3.58–17.83)
Syphilis	215 (377)	13,507/245,455	5.24 (4.83–5.69)	0.37 (0.20–0.65)* [21]	14.89 (10.86–20.40)
HSV-2	38 (57)	9,529/30,345	15.77 (11.74–20.85)	5.80 (3.82–7.78)* [22]	3.05 (2.12–4.39)
Trichomonas vaginitis	43 (65)	2,271/24,381	2.12 (1.46–24.21)	2.52 (1.92–3.31)** [22]	0.83 (0.51–1.35)
Chlamydia	96 (125)	7,983/56,548	13.66 (12.11–15.37)	2.60 (1.60–4.10)** [23]	5.95 (4.42–8.01)
Cervix	41 (78)	5,503/33,231	13.56 (11.65–15.73)	--	--
Vaginal	3 (4)	402/872	36.80 (19.56–58.23)	--	--
PCR	48 (74)	5,839/31,695	15.48(13.70–17.45)	--	--
Rapid test	16 (19)	2,144/24,853	10.28(8.20–12.83)	--	--
Gonorrhea	116 (149)	3,723/62,430	6.07 (5.29–6.95)	0.16 (0.06–0.39)** [23]	40.32 (12.99–125.18)
Cervix	51 (74)	2,523/33,493	6.88 (5.68–8.31)	--	--
Vaginal	7 (9)	311/1,806	12.69 (7.52–20.60)	--	--
PCR	28 (55)	2,015/23,179	8.33(6.83–10.13)	--	--
Culture	52 (64)	1,417/28,143	5.62(4.51–6.97)	--	--
Wart	68 (76)	1,111/39,862	3.27 (2.53–4.21)	0.06 (0.01–0.07)** [20]	52.96 (26.42–106.15)
HPV (any)	4 (4)	531/1,631	27.04 (10.07–55.09)	16.80 (16.50–17.10)** [24]	1.84 (1.64–2.06)
Gene type 16	3 (3)	142/1,143	14.08 (4.13–38.40)	3.62 (0.82–14.62)* [61, 62]	4.39 (3.20–6.03)
Gene type 52	3 (3)	150/1,143	12.53 (6.36–23.30)	2.62 (1.75–3.48)* [61, 62]	5.32 (3.72–7.63)
Gene type 58	2 (2)	115/1,098	12.12 (2.65–41.11)	1.59 (1.07–2.36)* [61, 62]	8.60 (5.53–13.38)
Gene type 33	3 (3)	75/1,143	8.25 (2.61–23.19)	1.04 (0.63–1.72)* [61, 62]	8.48 (4.96–14.48)
Gene type 31	2 (2)	59/1,098	5.05 (3.84–42.25)	2.09 (1.44–3.02)* [61, 62]	2.46 (1.58–3.82)
Multiple type	2 (2)	216/1,098	23.57 (6.08–59.47)	--	--
Single type	2 (2)	328/1,098	29.9(27.3–32.7)	--	--

* refers to *p* < 0.05

** refers to *p* < 0.01

### Disease burden of hepatitis

For studies conducted between 2000 and 2011, the estimated overall HBV and HCV prevalence among FSW were 10.7 % (95 % *CI*: 7.3–15.5 %) and 1.0 % (0.7–1.3 %) (Table 1). Heterogeneities across the studies were substantial (HBV, *p* < 0.001; *I* ^*2*^ = 97.1; HCV: *p* < 0.001; *I* ^*2*^ = 94.8). HBV disease burden varied substantially from 6.1 % (4.0–9.2 %) in the East to 15.5 % (9.5–24.2 %) in the South Central (Fig. 1a). No included studies reported HBV prevalence from FSW samples in the North and Northwest. In contrast, the Northwest had the lowest HCV prevalence (0.5 % [0.2–1.6 %]); while Southwest was the highest (2.0 % [1.1–3.6 %], Fig. 1a). The temporal trends of both of them were not significant (P > 0.05). Compared to the general Chinese population, FSW had notably higher risk of HBV and HCV infection (HBV: *OR* = 1.8 [1.6–1.9]; HCV: *OR* = 8.0 [3.6–17.8]) (Table 1).

### Disease burden of sexually transmitted infections

Meta-analysis indicated that the most common STI among FSW was HPV, with a pooled prevalence of 27.0 % (10.1–55.1 %). Notably, genotype HPV16 (14.1 % [4.1–38.4 %]) was the most frequently identified sub-type. About 23.6 % (6.1–59.5 %) of FSW were infected with multiple HPV sub-types. HSV-2 ranked second reaching 15.8 % (11.7–20.9 %) during 2000–2011, followed by Chlamydia at 13.7 % (12.1–15.4 %). Subgroup analyses indicated that Chlamydia prevalence substantially varied by anatomical sites, ranging from 13.6 % (11.7–15.7 %) checked by cervical smear to 36.8 % (19.6–58.2 %) checked by vaginal swab. The pooled prevalence estimates of syphilis, genital warts and *Trichomonas vaginitis* were 5.2 % (4.8–5.7 %), 3.3 % (2.5–4.2 %) and 2.1 % (1.5–24.2 %), respectively (Additional file 1: Table S2).

Risk of STIs among FSW was far higher than in the general population. The odds ratio (OR) for having genital warts among FSW was 81.4 (40.6–163.2) compared to the general population. This was followed by gonorrhoea (OR 40.3 [13.0–125.2]) and syphilis (OR 14.9 [10.9–20.4]) (Table 1). The prevalence of STIs was also higher among HIV-infected FSW compared to boarder FSW. Based on available data among HIV-positive FSW, prevalence of HSV-2, Chlamydia, *Trichomonas vaginitis* and syphilis were 83.2 % (75.6–88.8 %), 20.2 % (14.3–27.7 %), 19.7 % (12.3–30.2 %) and 15.2 % (8.3–26.4 %), respectively. These corresponded to 27.2 (17.0–43.5), 2.2 (1.4–3.3), 11.5 (5.7–23.0) and 4.1 (2.8–6.0) higher risk comparing to the broader FSW population (Table 2).

**Table 2:** Comparison of sexually transmitted infections prevalence between HIV-positive female sex workers and general female sex workers in China

Type of infection	No. of studies (No. of data points)	Total No of infection/Sample size	Pooled prevalence among HIV-positive FSW (95 % CI)	Pooled prevalence among FSW (95 % CI)	Odds ratio (95 % CI)
Chlamydia	2 (2)	28/139	20.21 (14.33–27.72)	13.66 (12.11–15.37)	2.15 (1.42–3.26)
HSV-2	2 (2)	107/128	83.22 (75.60–88.82)	15.77 (11.74–20.85)	13.6 (8.4–22.2)
Syphilis	5 (5)	27/210	15.22 (8.26–26.36)	5.24 (4.83–5.69)	4.13 (2.83–6.02)
Trichomonas vaginitis	1 (1)	15/76	19.74 (12.26–30.20)	2.12 (1.46–24.21)	11.48 (5.73–22.99)

### Temporal and geographical trend of STIs

**Fig. 2:**
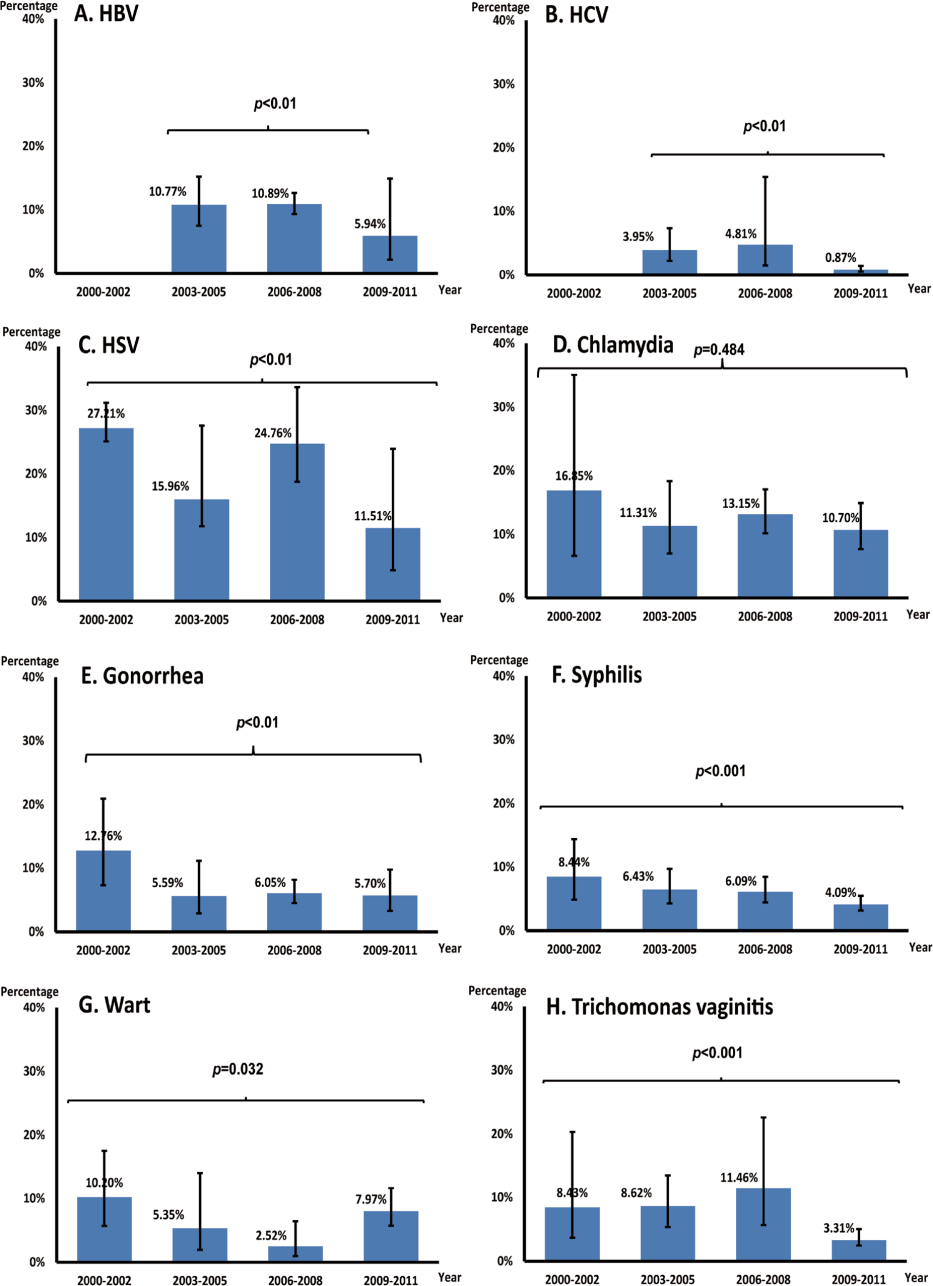
Estimated temporal trend of sexually transmitted infections among female sex workers in China during 2000–2011. *Only infection with > 10 papers were included, so the analysis excluded HPV because there are only four papers being found

Among sampled FSW, several STIs demonstrated a temporal declining trend. Over the study period (2000 to 2011), reports of Chlamydia declined from 16.9 % (6.6–35.1 %) in 2000–2002 to 10.7 % (7.7–14.9 %) in 2009–2011 (p = 0.02) while reports of syphilis declined from 8.4 % (4.9–14.4 %) to 4.1 % (3.2–5.5 %) (p < 0.01). The average annual decline in prevalence of chlamydia and syphilis were 9 % (1–16 %) and 2 % (0–5 %) respectively (Fig. 2). Temporal trends of other STIs did not show significant variations.

Southern Chinese regions had greater risk of STIs. In particular, FSW in the Southwest had high prevalence of HSV-2 (41.8 % [35.3–38.7 %]), Chlamydia (19.0 % [15.8–22.6 %]) and *Trichomonas Vaginitis* (11.8 % [9.1–15.2 %]) (Fig. 1a). The South Central region shared a similar disease burden of STIs as seen in the Southwest but had the highest nationwide prevalence of syphilis and genital warts (8.3 % [7.0–10.0 %] and 4.0 % [2.6–6.3 %], respectively). Prevalence of gonorrhoea among FSW was highest in the North (12.7 % [7.5–20.6 %]) and East (6.9 % [5.7–8.3 %]) regions (Fig. 1a). Overall, the prevalence of most STIs were low to moderate among FSW in the northern Chinese regions (Fig. 1b).

### Heterogeneities and risk of bias

Heterogeneities were significant in most of our meta-analyses (Additional file 1: Table S2). Larger sample size (≥400) tended to result in higher prevalence estimates (in all STIs except HSV-2). Prevalence of syphilis (8.0 % [7.4–10.1 %]), *Trichomonas Vaginitis* (13.2 % [7.9–21.1 %]) and gonorrhoea (7.9 % [5.9–10.6 %]) were substantially higher among FSW sampled from detention centres than among those sampled from entertainment venues (*p* < 0.05). Study quality score was not associated with prevalence estimates for any infections except HSV-2 (Additional file 1: Table S2).

## Discussion and conclusions

This meta-analysis has consistently demonstrated higher STI and viral hepatitis burden among FSWs in South Central and Southwest China compared to other Chinese regions. Compared with a previous similar systematic review [17], our study represents a significant extension in the investigation of temporal and geographical trend of STIs among FSW in reference to the general population. Apart from chlamydia and syphilis, prevalence levels of most STIs and hepatitis infections remain stable over the studied period. Compared with the general Chinese population, FSW had a significantly higher risk of STI and hepatitis infection. Similarly, HIV-positive FSW were more likely to have STIs than the broader FSW population.

Our study reported a national HBV prevalence of 10.7 % (HBsAg active) among Chinese FSW but with distinct regional differences. This prevalence level is higher than that of the general Chinese population (7.9 % in 2010, [26]) and the prevalence (7.18 %) reported by China CDC in 2006 [27]. Among all Chinese regions, the finding that South Central China (15.5 %) has shown the highest prevalence is consistent with the prevalence in the general Chinese population [13, 19]. HBV transmission in China is primarily through mother-to-child transmission [28, 29], but transmission by sex and injection sharing has also been reported in high-risk population groups [29]. The high HBV disease burden in Southern China is likely due to earlier onset of both the epidemic [30–32]. Also of note, China’s national HBV vaccination programme for newborns was only introduced in 1992 [33]; many of our studied subjects (average age 24.2) may have been born before the start or scale-up of the vaccination program. Vertical transmission may be the dominant route of transmission of HBV in FSW, but high risk sexual behaviours of FSW has also substantially added to the risk of HBV infection in comparison with the general Chinese females. Targeted interventions, orchestrated by China CDC and local NGOs, should be implemented in FSW to improve awareness and reduce further HBV transmission via sexual means and vertical transmission.

Southwest China has reported the highest prevalence of HCV (2.0 %). Southwest China has faced illicit drug trafficking issues due to its close proximity bordering the ‘golden triangle’, a well-known area for heroin production, manufacture and distribution in Southeast Asia [13]. It hosts estimated 3 million PWID and provides 42.7 % global seizures in heroin in 2013 [34]. Prevalent injection drug use may be the underlying reason for HCV transmission among FSW given that sexual transmission via heterosexual sex is rare [14, 35]. Among FSWs who use drugs, selling sex for drugs is notably more common in southern China than others regions [36, 37]. In order to alleviate the disease burden of HCV, specialised behavioural interventions for FSW with overlapping injecting and risk sexual behaviours should be designed and implemented.

We noted a fall in the prevalence of both chlamydia and syphilis over time. The fact that both of these infections are easily treatable and no significant fall has been observed in other viral infections suggest that testing and treatment of STIs either among sex workers or the general community may be the principle factor affecting their fall. On the other hand, syphilis is one of two STIs under sentinel surveillance in China and acquiring more attention from the local CDC. Thus the variation may be attributable to policy changes related to the scaling-up of syphilis testing in pregnancy [38] and as part of the 2010–2020 syphilis control plan [39]. Building on declines in chlamydia and syphilis, continued intervention efforts are needed to achieve a decrease in viral infections as well.

HPV is the most prevalent STI among Chinese FSWs (27.0 %), as well as in the general Chinese population (16.8 %, [24]). Further, HPV prevalence among Chinese FSWs appears to be higher than that found in neighbouring countries (22.9 % in Thailand [40] and 14.4 % in Singapore [41]), a finding also true for genital warts: 3.3 % in Chinese FSW versus 0.2 % in neighbouring Vietnam [42]. Genital warts are mostly caused by infection with HPV-6 or 11 [43, 44], but more virulent types of HPV (type 16 and 18) are major risk factors for cervical cancers in Chinese women [24, 45, 46]. Co-infection with HPV is also known to facilitate the transmission of chlamydia, gonorrhoea, HSV-2 and HIV [47]. Despite the evidence that early vaccination before sexual debut dramatically reduces the risk of HPV infection, genital warts and cervical cancers among females [48–50], currently there is no national HPV vaccination programme and no specific prevention method for women of reproductive age in China [51]. The majority of Chinese women (approximately 70 %) have no knowledge about HPV vaccines [52] and even lower levels of knowledge and awareness of HPV in FSW [53, 54]. Given the very high disease burden of HPV, Chinese FSW should be regarded as a priority population for HPV prevention and treatment, especially FSW who are of reproductive age.

A number of limitations should be noted. First, we reported high heterogeneities in our meta-analyses. Despite the efforts of subgroup analysis and meta-regression, significant heterogeneities were still present in subgroups. We have analysed the influences of geographical region, recruitment venue, sample size, publication language, study quality score, and year of publication. Second, statistical power in subgroup analysis was often restricted by the limited number of available studies, especially among the small number of studies from the Northeast. Third, although we only included studies diagnosed with the same methodology, the diagnostic accuracy of tests maybe improved during the study period, so part of the declining trend in prevalence may be owing to the improvement of technology. Fourth, publication bias in the publication language was significant. Fifth, the different clinical diagnostic standards of STIs and hepatitis infections may result in uncertainties in sensitivity and specificity of results.

Our findings illustrated above are consistent with previous reports [7, 55–58]. We have extended the previous literature by quantifying these risks in a systematic manner to demonstrate the urgency for appropriate prevention and treatment strategies among Chinese FSW, especially those co-infected with HIV. Health resources should be aligned to address these issues. Prevalence of hepatitis infection and STIs in Chinese FSWs is high but stable during the study period 2000–2011. The high prevalence of STIs may relate to the illegal status of sex work. Ongoing research indicates that countries with either regulated or legalized sex work have seen substantial falls in the prevalence of STIs in sex workers, as a result of governments’ initiatives for wide-spread condom programs and promotion of HIV testing and treatment [59, 60]. China is unlikely to legalize sex work in the near future, therefore comprehensive interventions and HIV programs for FSWs through community-based organizations should be promoted and prioritised.

## Additional files

Additional file 1: **Table S1.** Systematic review of 347 studies reporting the prevalence of sexually transmitted infections and/or viral hepatitis infections among female sex workers in China. **Table S2.** Systematic review of 19 studies reporting the co-infection prevalence of sexually transmitted infections and/or viral hepatitis infections among HIV-positive female sex workers in China. **Table S3.** Heterogeneity in subgroup meta-analyses. (DOCX 687 kb)

Additional file 2: **PRISMA flow chart for selection of studies.** (PNG 159 kb)
