# Supplementary material for: Sustained high prevalence of viral hepatitis and sexually transmissible infections among female sex workers in China: a systematic review and meta-analysis
Source: BMC Infect Dis. 2016 Jan 5;16:2. doi: 10.1186/s12879-015-1322-0 (PMC4702370; doi:10.1186/s12879-015-1322-0)
Supplement: Supplementary file 1 — Table S1. Systematic review of 347 studies reporting the prevalence of sexually transmitted infections and/or viral hepatitis infections among female sex workers in China. Table S2. Systematic review of 19 studies reporting the co-infection prevalence of sexually transmitted infections and/or viral hepatitis infections among HIV-positive female sex workers in China. Table S3. Heterogeneity in subgroup meta-analyses. (DOCX 687 kb) [file 12879_2015_1322_MOESM1_ESM.docx]

**Table S1. Systematic review of 347 studies reporting the prevalence of sexually transmitted infections and/or viral hepatitis infections among female sex workers in China.**

| **Study** | **Year** | **Province** | **Region** | **Sampling method** | **Venue** | **Quality score** | **Language** | **Disease Prevalence** | | |  |  |  |  |  |  |  |  |  |  |
| --- | --- | --- | --- | --- | --- | --- | --- | --- | --- | --- | --- | --- | --- | --- | --- | --- | --- | --- | --- | --- |
|  |  |  |  |  |  |  |  | **Sample size, N** | **n** | **Prevalence (%)** |  |  |  |  |  |  |  |  |  |  |
| *a) Chlamydia* | | | | | | | | | | |  |  |  |  |  |  |  |  |  |  |
| Wei CQ, 2007[[1](#_ENREF_1)] | 2006 |  |  | Cross-sectional | Entertainment venues | 3 | Chinese | 280 | 33 | 11.79% |  |  |  |  |  |  |  |  |  |  |
| Liu SF, 2007[[2](#_ENREF_2)] |  | Jiangsu | East | Cross-sectional | Entertainment venues | 4 | Chinese | 214 | 56 | 26.20% |  |  |  |  |  |  |  |  |  |  |
| Zhang ZX, 2009[[3](#_ENREF_3)] |  | Fujian | East | Cross-sectional | Others | 2 | Chinese | 128 | 14 | 10.90% |  |  |  |  |  |  |  |  |  |  |
| Zheng BZ, 2003[[4](#_ENREF_4)] | 2000/06 | Zhejiang | East | Cross-sectional | Entertainment venues | 5 | Chinese | 176 | 140 | 79.55% |  |  |  |  |  |  |  |  |  |  |
| Du YP, 2004a[[5](#_ENREF_5)] | 2001/11 | Jiangsu | East | Cross-sectional | Entertainment venues | 5 | Chinese | 303 | 64 | 21.10% |  |  |  |  |  |  |  |  |  |  |
| Yan HJ, 2004[[6](#_ENREF_6)] | 2002/04 | Jiangsu | East | Cross-sectional | Detaining education center | 5 | Chinese | 50 | 9 | 18.00% |  |  |  |  |  |  |  |  |  |  |
| Yang P, 2005a[[7](#_ENREF_7)] | 2002/12 | Fujian | East | Cross-sectional | Entertainment venues | 3 | Chinese | 149 | 41 | 27.52% |  |  |  |  |  |  |  |  |  |  |
| Xu LL, 2006a[[8](#_ENREF_8)] | 2003/04-2004/10 | Shanghai | East | Cross-sectional | Entertainment venues | 4 | Chinese | 191 | 7 | 3.70% |  |  |  |  |  |  |  |  |  |  |
| Lin YQ, 2005[[9](#_ENREF_9)] | 2003/09 | Zhejiang | East | Cross-sectional | Detaining education center | 3 | Chinese | 181 | 39 | 21.55% |  |  |  |  |  |  |  |  |  |  |
| Wang FJ, 2008[[10](#_ENREF_10)] | 2003-2006 | Zhejiang | East | Cross-sectional | Detaining education center | 3 | Chinese | 608 | 38 | 6.25% |  |  |  |  |  |  |  |  |  |  |
| Zhang Y, 2005[[11](#_ENREF_11)] | 2004/04-2004/05 | Shandong | East | Cross-sectional | Entertainment venues | 3 | Chinese | 180 | 3 | 1.70% |  |  |  |  |  |  |  |  |  |  |
| NI CM, 2011[[12](#_ENREF_12)] | 2004-2008 | Zhejiang | East | Cross-sectional | Entertainment venues | 6 | Chinese | 405 | 20 | 4.90% |  |  |  |  |  |  |  |  |  |  |
| Zeng YB, 2009a[[13](#_ENREF_13)] | 2005 | Shanghai | East | National Sentinel Site | Detaining education center | 4 | Chinese | 448 | 18 | 5.02% |  |  |  |  |  |  |  |  |  |  |
| Yan HJ, 2007[[14](#_ENREF_14)] | 2005 | Jiangsu | East | Cross-sectional | Entertainment venues | 3 | Chinese | 1,417 | 315 | 22.20% |  |  |  |  |  |  |  |  |  |  |
| Zeng YB, 2009b[[13](#_ENREF_13)] | 2006 | Shanghai | East | National Sentinel Site | Detaining education center | 4 | Chinese | 448 | 11 | 2.46% |  |  |  |  |  |  |  |  |  |  |
| Xu HQ, 2008[[15](#_ENREF_15)] | 2006 | Zhejiang | East | Cross-sectional | Entertainment venues | 5 | Chinese | 84 | 1 | 1.20% |  |  |  |  |  |  |  |  |  |  |
| Shu CH, 2011[[16](#_ENREF_16)] | 2006/03-2009/05 | Jiangsu | East | Cross-sectional | Detaining education center | 4 | Chinese | 225 | 38 | 16.90% |  |  |  |  |  |  |  |  |  |  |
| Hu JL, 2007[[17](#_ENREF_17)] | 2006/04-2006/05 | Anhui | East | Cross-sectional | Entertainment venues | 5 | Chinese | 256 | 57 | 24.37% |  |  |  |  |  |  |  |  |  |  |
| Yu X, 2007[[18](#_ENREF_18)] | 2006/05 | Jiangsu | East | Cross-sectional | Entertainment venues | 5 | Chinese | 121 | 4 | 3.31% |  |  |  |  |  |  |  |  |  |  |
| Zhu FG, 2009a[[19](#_ENREF_19)] | 2006/05 | Jiangsu | East | Cross-sectional | Entertainment venues | 4 | Chinese | 252 | 40 | 13.39% |  |  |  |  |  |  |  |  |  |  |
| Zeng YB, 2009c[[13](#_ENREF_13)] | 2007 | Shanghai | East | National Sentinel Site | Detaining education center | 4 | Chinese | 448 | 3 | 0.67% |  |  |  |  |  |  |  |  |  |  |
| Wang WM, 2008[[20](#_ENREF_20)] | 2007/01-2007/12 | Jiangsu | East | Cross-sectional | Detaining education center | 4 | Chinese | 297 | 34 | 11.45% |  |  |  |  |  |  |  |  |  |  |
| Yang JY, 2011[[21](#_ENREF_21)] | 2007/10-2007/11 | Jiangsu | East | Cross-sectional | Entertainment venues | 4 | Chinese | 247 | 11 | 4.45% |  |  |  |  |  |  |  |  |  |  |
| Chen JQ, 2009[[22](#_ENREF_22)] | 2008/01-2008/10 | Zhejiang | East | Cross-sectional | Others | 3 | Chinese | 168 | 123 | 73.21% |  |  |  |  |  |  |  |  |  |  |
| Zhao YQ, 2010a[[23](#_ENREF_23)] | 2008/04-2008/05 | Jiangsu | East | Cross-sectional | Entertainment venues | 4 | Chinese | 396 | 30 | 7.60% |  |  |  |  |  |  |  |  |  |  |
| Wang F, 2010a[[24](#_ENREF_24)] | 2009 | Anhui | East | Cross-sectional | Entertainment venues | 4 | Chinese | 75 | 6 | 8.00% |  |  |  |  |  |  |  |  |  |  |
| Li XY, 2011[[25](#_ENREF_25)] | 2009/01-2010/05 | Zhejiang | East | Cross-sectional | Detaining education center | 3 | Chinese | 98 | 12 | 12.24% |  |  |  |  |  |  |  |  |  |  |
| Zhang QQ, 2012[[26](#_ENREF_26)] | 2009/07-2009/09 | Jiangsu | East | Cross-sectional | Entertainment venues | 4 | Chinese | 1,806 | 125 | 6.92% |  |  |  |  |  |  |  |  |  |  |
| Wei ZY, 2010a[[27](#_ENREF_27)] | 2009/07-2009/09 | Jiangsu | East | Cross-sectional | Entertainment venues | 5 | Chinese | 940 | 55 | 13.32% |  |  |  |  |  |  |  |  |  |  |
| Zhang QQ, 2012[[26](#_ENREF_26)] | 2009/10-2009/12 | Jiangsu | East | Cross-sectional | Entertainment venues | 4 | Chinese | 737 | 48 | 13.50% |  |  |  |  |  |  |  |  |  |  |
| Tang ZL, 2011[[28](#_ENREF_28)] | 2010/03-2010/07 | Shandong | East | Cross-sectional | Entertainment venues | 4 | Chinese | 106 | 28 | 26.40% |  |  |  |  |  |  |  |  |  |  |
| Tang ZL, 2011[[28](#_ENREF_28)] | 2010/03-2010/07 | Shandong | East | Cross-sectional | Entertainment venues | 4 | Chinese | 216 | 46 | 21.30% |  |  |  |  |  |  |  |  |  |  |
| Li DL, 2006a[[29](#_ENREF_29)] | 2001 | Beijing | North | Cross-sectional | National sentinel site | 4 | Chinese | 354 | 38 | 11.18% |  |  |  |  |  |  |  |  |  |  |
| Zhao YR, 2003[[30](#_ENREF_30)] | 2001/07-2002/07 | Beijing | North | Cross-sectional | Detaining education center | 6 | Chinese | 510 | 143 | 28.04% |  |  |  |  |  |  |  |  |  |  |
| Li DL, 2006b[[29](#_ENREF_29)] | 2002 | Beijing | North | Cross-sectional | National sentinel site | 4 | Chinese | 571 | 18 | 3.52% |  |  |  |  |  |  |  |  |  |  |
| Li DL, 2006c[[29](#_ENREF_29)] | 2003 | Beijing | North | Cross-sectional | National sentinel site | 4 | Chinese | 174 | 2 | 7.30% |  |  |  |  |  |  |  |  |  |  |
| Li DL, 2006d[[29](#_ENREF_29)] | 2004 | Beijing | North | Cross-sectional | National sentinel site | 4 | Chinese | 413 | 32 | 7.75% |  |  |  |  |  |  |  |  |  |  |
| Li Y, 2011[[31](#_ENREF_31)] | 2010 | Heilongjiang | Northeast | Cross-sectional | National sentinel site | 4 | Chinese | 7,662 | 38 | 0.50% |  |  |  |  |  |  |  |  |  |  |
| Lu H, 2003[[32](#_ENREF_32)] | 2002 | Xinjiang | Northwest | Cross-sectional | Detaining education center | 2 | Chinese | 346 | 112 | 32.37% |  |  |  |  |  |  |  |  |  |  |
| Xi SH, 2004[[33](#_ENREF_33)] | 2002/05-2002/07 | Gansu | Northwest | Cross-sectional | Entertainment venues | 7 | Chinese | 88 | 43 | 48.90% |  |  |  |  |  |  |  |  |  |  |
| Wei XW, 2005[[34](#_ENREF_34)] | 2003/03-2003/05 | Xinjiang | Northwest | Cross-sectional | Entertainment venues | 2 | Chinese | 459 | 3 | 0.65% |  |  |  |  |  |  |  |  |  |  |
| Li F, 2006[[35](#_ENREF_35)] | 2004/04-2004/06 | Xinjiang | Northwest | Cross-sectional | Entertainment venues | 6 | Chinese | 611 | 135 | 22.09% |  |  |  |  |  |  |  |  |  |  |
| Wang HZ, 2006[[36](#_ENREF_36)] | 2005/06 | Xinjiang | Northwest | Cross-sectional | Entertainment venues | 3 | Chinese | 366 | 61 | 16.70% |  |  |  |  |  |  |  |  |  |  |
| Song Y, 2006[[37](#_ENREF_37)] | 2006 | Xinjiang | Northwest | Cross-sectional | Entertainment venues | 4 | Chinese | 103 | 24 | 23.30% |  |  |  |  |  |  |  |  |  |  |
| Yang SP, 2008a[[38](#_ENREF_38)] | 2007/08-2007/09 | Xinjiang | Northwest | Cross-sectional | Entertainment venues | 3 | Chinese | 251 | 32 | 3.19% |  |  |  |  |  |  |  |  |  |  |
| A SI YA, 2011[[39](#_ENREF_39)] | 2008/07-2008/09 | Xinjiang | Northwest | Cross-sectional | Others | 2 | Chinese | 200 | 14 | 7.10% |  |  |  |  |  |  |  |  |  |  |
| Gao W, 2012 (a)[[40](#_ENREF_40)] | 2008/11-2009/01 | Gansu | Northwest | Cross-sectional | Entertainment venues | 7 | English | 350 | 21 | 6.00% |  |  |  |  |  |  |  |  |  |  |
| Zhang CJ, 2001[[41](#_ENREF_41)] | 2000/03-2000/08 | Henan | South Central | Cross-sectional | Detaining education center | 3 | Chinese | 65 | 8 | 12.31% |  |  |  |  |  |  |  |  |  |  |
| Zhnagdan C, 2008[[42](#_ENREF_42)] | 2001/10 | Hubei | South Central | Cross-sectional | Entertainment venues | 3 | English | 170 | 37 | 27.16% |  |  |  |  |  |  |  |  |  |  |
| Hu B, 2004a[[43](#_ENREF_43)] | 2002 | Hainan | South Central | Cross-sectional | Entertainment venues | 5 | Chinese | 422 | 47 | 11.03% |  |  |  |  |  |  |  |  |  |  |
| Liang YW, 2003[[44](#_ENREF_44)] | 2002/09 | Hainan | South Central | Cross-sectional | Entertainment venues | 0 | Chinese | 62 | 24 | 38.71% |  |  |  |  |  |  |  |  |  |  |
| He QY, 2003[[45](#_ENREF_45)] | 2002/09 | Hainan | South Central | Cross-sectional | Entertainment venues | 5 | Chinese | 417 | 47 | 11.03% |  |  |  |  |  |  |  |  |  |  |
| Luo J, 2005c[[46](#_ENREF_46)] | 2003/06 | Guangxi | South Central | Cross-sectional | Entertainment venues | 4 | Chinese | 126 | 20 | 15.87% |  |  |  |  |  |  |  |  |  |  |
| Luo J, 2005a[[46](#_ENREF_46)] | 2003/06 | Guangxi | South Central | Cross-sectional | Entertainment venues | 4 | Chinese | 278 | 70 | 25.18% |  |  |  |  |  |  |  |  |  |  |
| Luo J, 2005e[[46](#_ENREF_46)] | 2003/06 | Guangxi | South Central | Cross-sectional | Entertainment venues | 4 | Chinese | 152 | 50 | 32.89% |  |  |  |  |  |  |  |  |  |  |
| Peng SQ, 2004[[47](#_ENREF_47)] | 2003/09-2003/12 | Guangdong | South Central | Cross-sectional | Detaining education center | 4 | Chinese | 110 | 26 | 23.64% |  |  |  |  |  |  |  |  |  |  |
| Tang GB, 2007a[[48](#_ENREF_48)] | 2004 | Hunan | South Central | Cross-sectional | Entertainment venues | 4 | Chinese | 397 | 24 | 6.00% |  |  |  |  |  |  |  |  |  |  |
| Xiong CB, 2006a[[49](#_ENREF_49)] | 2004 | Hunan | South Central | Cross-sectional | Entertainment venues | 4 | Chinese | 136 | 43 | 31.60% |  |  |  |  |  |  |  |  |  |  |
| Hong Y, 2009[[50](#_ENREF_50)] | 2004 | Guangxi | South Central | Cross-sectional | Entertainment venues | 4 | English | 411 | 77 | 18.70% |  |  |  |  |  |  |  |  |  |  |
| Wang H, 2005[[51](#_ENREF_51)] | 2004/03-2004/05 | Guangxi | South Central | Cross-sectional | Entertainment venues | 4 | English | 410 | 77 | 18.90% |  |  |  |  |  |  |  |  |  |  |
| Zhou YJ, 2007[[52](#_ENREF_52)] | 2004/05 | Guangxi | South Central | Cross-sectional | Entertainment venues | 6 | Chinese | 80 | 7 | 8.80% |  |  |  |  |  |  |  |  |  |  |
| Jiang M, 2005[[53](#_ENREF_53)] | 2004/08-2004/10 | Hubei | South Central | Cross-sectional | Entertainment venues | 4 | Chinese | 211 | 10 | 9.35% |  |  |  |  |  |  |  |  |  |  |
| Yang BF, 2006a[[54](#_ENREF_54)] | 2004/09 | Hubei | South Central | Cross-sectional | Entertainment venues | 2 | Chinese | 577 | 82 | 14.21% |  |  |  |  |  |  |  |  |  |  |
| Zhang WS, 2006a[[55](#_ENREF_55)] | 2004/10-2005/04 | Hubei | South Central | Cross-sectional | Entertainment venues | 4 | Chinese | 228 | 65 | 28.51% |  |  |  |  |  |  |  |  |  |  |
| Wu MS, 2006[[56](#_ENREF_56)] | 2005/03 | Guangxi | South Central | Cross-sectional | Entertainment venues | 2 | Chinese | 187 | 47 | 25.13% |  |  |  |  |  |  |  |  |  |  |
| Zhang JL, 2008a[[57](#_ENREF_57)] | 2006/01-2007/05 | Guangxi | South Central | Cross-sectional | Entertainment venues | 4 | Chinese | 59 | 12 | 20.34% |  |  |  |  |  |  |  |  |  |  |
| Li CG, 2007a[[58](#_ENREF_58)] | 2006/05 | Guangdong | South Central | Cross-sectional | Entertainment venues | 6 | Chinese | 522 | 74 | 14.18% |  |  |  |  |  |  |  |  |  |  |
| Li Y, 2012[[59](#_ENREF_59)] | 2006/08 - 2007/01 | Guangdong | South Central | Cross-sectional | Entertainment venues | 6 | English | 318 | 10 | 3.20% |  |  |  |  |  |  |  |  |  |  |
| Li Y, 2009[[60](#_ENREF_60)] | 2006/08-2007/01 | Guangdong | South Central | Cross-sectional | Entertainment venues | 6 | Chinese | 320 | 12 | 3.75% |  |  |  |  |  |  |  |  |  |  |
| Wu ZZ, 2008[[61](#_ENREF_61)] | 2006/11-2007/01 | Guangdong | South Central | Cross-sectional | Entertainment venues | 1 | Chinese | 213 | 38 | 17.84% |  |  |  |  |  |  |  |  |  |  |
| Zhu BY, 2012[[62](#_ENREF_62)] | 2007/05-2007/10 | Guangxi | South Central | Cross-sectional | Entertainment venues | 6 | English | 488 | 89 | 18.24% |  |  |  |  |  |  |  |  |  |  |
| Jiang M, 2012[[63](#_ENREF_63)] | 2007/07-2007/10 | Hubei | South Central | Cross-sectional | Entertainment venues | 4 | Chinese | 211 | 10 | 9.30% |  |  |  |  |  |  |  |  |  |  |
| Yi QM, 2009b[[64](#_ENREF_64)] | 2008/09 | Henan | South Central | Cross-sectional | Entertainment venues | 3 | Chinese | 88 | 9 | 10.20% |  |  |  |  |  |  |  |  |  |  |
| Yi QM, 2009c[[64](#_ENREF_64)] | 2008/09 | Henan | South Central | Cross-sectional | Entertainment venues | 3 | Chinese | 85 | 10 | 11.76% |  |  |  |  |  |  |  |  |  |  |
| Yi QM, 2009d[[64](#_ENREF_64)] | 2008/09 | Henan | South Central | Cross-sectional | Entertainment venues | 3 | Chinese | 127 | 15 | 11.81% |  |  |  |  |  |  |  |  |  |  |
| Zhao YY, 2010[[65](#_ENREF_65)] | 2008/10-2008/12 | Guangdong | South Central | Cross-sectional | Entertainment venues | 4 | Chinese | 622 | 64 | 10.29% |  |  |  |  |  |  |  |  |  |  |
| Xiang SB, 2010b[[66](#_ENREF_66)] | 2009 | Hunan | South Central | Cross-sectional | Entertainment venues | 4 | Chinese | 401 | 3 | 0.75% |  |  |  |  |  |  |  |  |  |  |
| Zhong J, 2011[[67](#_ENREF_67)] | 2009/06-2009/08 | Guangxi | South Central | Cross-sectional | Entertainment venues | 4 | Chinese | 1,166 | 88 | 7.55% |  |  |  |  |  |  |  |  |  |  |
| Zhong J, 2011[[67](#_ENREF_67)] | 2009/06-2009/08 | Guangxi | South Central | Cross-sectional | Entertainment venues | 4 | Chinese | 1,166 | 88 | 7.55% |  |  |  |  |  |  |  |  |  |  |
| Zhong J, 2010a[[68](#_ENREF_68)] | 2009/06-2009/08 | Guangxi | South Central | Cross-sectional | Entertainment venues | 5 | Chinese | 406 | 88 | 21.67% |  |  |  |  |  |  |  |  |  |  |
| Zhong J, 2010c[[68](#_ENREF_68)] | 2009/06-2009/08 | Guangxi | South Central | Cross-sectional | Entertainment venues | 5 | Chinese | 80 | 14 | 17.50% |  |  |  |  |  |  |  |  |  |  |
| Zhong J, 2010d[[68](#_ENREF_68)] | 2009/06-2009/08 | Guangxi | South Central | Cross-sectional | Entertainment venues | 5 | Chinese | 165 | 33 | 20.00% |  |  |  |  |  |  |  |  |  |  |
| Zhong J, 2010e[[68](#_ENREF_68)] | 2009/06-2009/08 | Guangxi | South Central | Cross-sectional | Entertainment venues | 5 | Chinese | 161 | 41 | 25.47% |  |  |  |  |  |  |  |  |  |  |
| Zhao JZ, 2011[[69](#_ENREF_69)] | 2009/07-2009/12 | Hubei | South Central | Cross-sectional | Entertainment venues | 4 | Chinese | 52 | 31 | 59.60% |  |  |  |  |  |  |  |  |  |  |
| Zhu L, 2011[[70](#_ENREF_70)] | 2010/04-2010/06 | Hubei | South Central | Cross-sectional | National sentinel site | 4 | Chinese | 402 | 1 | 0.20% |  |  |  |  |  |  |  |  |  |  |
| Chen ZH, 2007a[[71](#_ENREF_71)] |  | Guangdong | South Central | Cross-sectional | Entertainment venues | 3 | Chinese | 1,200 | 84 | 7.00% |  |  |  |  |  |  |  |  |  |  |
| Yang P, 2006[[72](#_ENREF_72)] |  | Sichuan | Southwest | Cross-sectional | Entertainment venues | 2 | Chinese | 407 | 138 | 33.90% |  |  |  |  |  |  |  |  |  |  |
| Wu Y, 2002a[[73](#_ENREF_73)] | 2001/09 | Chongqing | Southwest | Cohort | Entertainment venues | 6 | Chinese | 184 | 24 | 13.00% |  |  |  |  |  |  |  |  |  |  |
| Wu Y, 2002b[[73](#_ENREF_73)] | 2001/09 | Chongqing | Southwest | Cohort | Detaining education center | 6 | Chinese | 195 | 23 | 11.80% |  |  |  |  |  |  |  |  |  |  |
| Wang QQ, 2005[[74](#_ENREF_74)] | 2004/02 | Sichuan | Southwest | Cross-sectional | Entertainment venues | 3 | Chinese | 206 | 71 | 34.50% |  |  |  |  |  |  |  |  |  |  |
| Wu Y, 2007a[[75](#_ENREF_75)] | 2004/09 | Chongqing | Southwest | Cohort | Entertainment venues | 4 | Chinese | 157 | 9 | 3.20% |  |  |  |  |  |  |  |  |  |  |
| Wu Y, 2007b[[75](#_ENREF_75)] | 2004/09 | Chongqing | Southwest | Cohort | Entertainment venues | 4 | Chinese | 169 | 3 | 1.80% |  |  |  |  |  |  |  |  |  |  |
| Dai XQ, 2007[[76](#_ENREF_76)] | 2005/06-2005/09 | Chongqing | Southwest | Cross-sectional | Entertainment venues | 5 | Chinese | 341 | 47 | 15.60% |  |  |  |  |  |  |  |  |  |  |
| Tan XJ, 2007[[77](#_ENREF_77)] | 2005/08-2005/09 | Chongqing | Southwest | Cross-sectional | Entertainment venues | 5 | Chinese | 266 | 30 | 11.30% |  |  |  |  |  |  |  |  |  |  |
| Ding XB, 2006[[78](#_ENREF_78)] | 2005/08-2005/09 | Chongqing | Southwest | Cross-sectional | Entertainment venues | 4 | Chinese | 519 | 78 | 14.50% |  |  |  |  |  |  |  |  |  |  |
| Wang HB, 2010a[[79](#_ENREF_79)] | 2006/03 | Yunnan | Southwest | Cross-sectional | Entertainment venues | 7 | English | 737 | 191 | 25.90% |  |  |  |  |  |  |  |  |  |  |
| Xu J, 2011[[80](#_ENREF_80)] | 2006/03 - 2007/04 | Yunnan | Southwest | Cohort | Others | 3 | English | 1,642 | 432 | 26.31% |  |  |  |  |  |  |  |  |  |  |
| Wang H, 2012[[81](#_ENREF_81)] | 2006/03 - 2009/11 | Yunnan | Southwest | Cohort | Entertainment venues | 6 | English | 851 | 184 | 21.60% |  |  |  |  |  |  |  |  |  |  |
| Li QH, 2010a[[82](#_ENREF_82)] | 2006/03-2006/04 | Yunnan | Southwest | Cross-sectional | Entertainment venues | 6 | Chinese | 716 | 187 | 26.10% |  |  |  |  |  |  |  |  |  |  |
| Xu JJ, 2008[[83](#_ENREF_83)] | 2006/03-2006/04 | Yunnan | Southwest | Cross-sectional | Entertainment venues | 6 | English | 95 | 44 | 46.30% |  |  |  |  |  |  |  |  |  |  |
| Wang H, 2009[[84](#_ENREF_84)] | 2006/03-2006/05 | Yunnan | Southwest | Cross-sectional | Entertainment venues | 4 | English | 737 | 191 | 25.92% |  |  |  |  |  |  |  |  |  |  |
| Wang H, 2009e[[84](#_ENREF_84)] | 2006/03-2006/05 | Yunnan | Southwest | Cross-sectional | Entertainment venues | 4 | English | 458 | 108 | 23.60% |  |  |  |  |  |  |  |  |  |  |
| Wang H, 2009f[[84](#_ENREF_84)] | 2006/03-2006/05 | Yunnan | Southwest | Cross-sectional | Entertainment venues | 4 | English | 279 | 83 | 29.70% |  |  |  |  |  |  |  |  |  |  |
| Wang HB, 2010a[[79](#_ENREF_79)] | 2006/03-2006/12 | Yunnan | Southwest | Cross-sectional | Entertainment venues | 6 | English | 1,484 | 338 | 22.78% |  |  |  |  |  |  |  |  |  |  |
| Wang H, 2011[[85](#_ENREF_85)] | 2006/03-2009/11 | Yunnan | Southwest | Cohort | Entertainment venues | 6 | English | 741 | 173 | 23.30% |  |  |  |  |  |  |  |  |  |  |
| Wang H, 2011[[86](#_ENREF_86)] | 2006/03-2009/11 | Yunnan | Southwest | Cohort | Entertainment venues | 4 | English | 1,836 | 457 | 24.89% |  |  |  |  |  |  |  |  |  |  |
| Luo XR, 2008a[[87](#_ENREF_87)] | 2006/05-2006/06 | Sichuan | Southwest | Cross-sectional | Entertainment venues | 5 | Chinese | 194 | 47 | 24.23% |  |  |  |  |  |  |  |  |  |  |
| Li QH, 2009a[[88](#_ENREF_88)] | 2006/09-2006/10 | Yunnan | Southwest | Cross-sectional | Entertainment venues | 7 | Chinese | 335 | 59 | 17.61% |  |  |  |  |  |  |  |  |  |  |
| Li QH, 2009b[[88](#_ENREF_88)] | 2006/09-2006/10 | Yunnan | Southwest | Cross-sectional | Entertainment venues | 7 | Chinese | 312 | 67 | 21.47% |  |  |  |  |  |  |  |  |  |  |
| Li QH, 2009c[[88](#_ENREF_88)] | 2006/09-2006/10 | Yunnan | Southwest | Cross-sectional | Entertainment venues | 7 | Chinese | 87 | 21 | 24.14% |  |  |  |  |  |  |  |  |  |  |
| Wang GX, 2008a[[89](#_ENREF_89)] | 2006/09-2006/10 | Yunnan | Southwest | Cross-sectional | Entertainment venues | 5 | Chinese | 734 | 147 | 20.03% |  |  |  |  |  |  |  |  |  |  |
| Wang GX, 2008b[[89](#_ENREF_89)] | 2006/09-2006/10 | Yunnan | Southwest | Cross-sectional | Entertainment venues | 5 | Chinese | 266 | 58 | 21.80% |  |  |  |  |  |  |  |  |  |  |
| Wang GX, 2008c[[89](#_ENREF_89)] | 2006/09-2006/10 | Yunnan | Southwest | Cross-sectional | Entertainment venues | 5 | Chinese | 351 | 92 | 26.21% |  |  |  |  |  |  |  |  |  |  |
| Wang GX, 2008d[[89](#_ENREF_89)] | 2006/09-2006/10 | Yunnan | Southwest | Cross-sectional | Entertainment venues | 5 | Chinese | 120 | 41 | 34.17% |  |  |  |  |  |  |  |  |  |  |
| Wang HB, 2010b[[79](#_ENREF_79)] | 2006/10 | Yunnan | Southwest | Cross-sectional | Entertainment venues | 5 | English | 747 | 147 | 19.70% |  |  |  |  |  |  |  |  |  |  |
| Gao LL, 2008a[[90](#_ENREF_90)] | 2007 | Yunnan | Southwest | Cross-sectional | Detaining education center | 4 | Chinese | 270 | 6 | 2.20% |  |  |  |  |  |  |  |  |  |  |
| Xu JJ, 2012[[91](#_ENREF_91)] | 2007/03-2007-07 | Yunnan | Southwest | Cross-sectional | Entertainment venues | 6 | English | 705 | 128 | 18.10% |  |  |  |  |  |  |  |  |  |  |
| Wang HB, 2010c[[79](#_ENREF_79)] | 2007/05 | Yunnan | Southwest | Cross-sectional | Entertainment venues | 5 | English | 705 | 126 | 17.90% |  |  |  |  |  |  |  |  |  |  |
| Wang HB, 2010b[[79](#_ENREF_79)] | 2007/05 | Yunnan | Southwest |  | Others | 5 | English | 705 | 126 | 17.87% |  |  |  |  |  |  |  |  |  |  |
| Yao Y, 2012[[92](#_ENREF_92)] | 2007/09-2007/10 | Yunnan | Southwest | Cross-sectional | Entertainment venues | 7 | English | 373 | 85 | 22.80% |  |  |  |  |  |  |  |  |  |  |
| Jin X, 2011[[93](#_ENREF_93)] | 2008/04 - 2008/05 | Yunnan | Southwest | Cross-sectional | Entertainment venues | 7 | English | 568 | 99 | 17.40% |  |  |  |  |  |  |  |  |  |  |
| Jin X, 2011[[93](#_ENREF_93)] | 2008/04-2008/05 | Yunnan | Southwest | Cross-sectional | Entertainment venues | 5 | English | 568 | 99 | 17.40% |  |  |  |  |  |  |  |  |  |  |
| Yan WZ, 2011[[94](#_ENREF_94)] | 2009 | Yunnan | Southwest | Cross-sectional | Entertainment venues | 3 | Chinese | 180 | 2 | 1.11% |  |  |  |  |  |  |  |  |  |  |
| Zhang XD, 2012[[95](#_ENREF_95)] | 2010/07-2011/02 | Yunnan | Southwest | Cross-sectional | Entertainment venues | 6 | English | 191 | 28 | 14.70% |  |  |  |  |  |  |  |  |  |  |
| Shao ZP, 2012[[96](#_ENREF_96)] | 2011/05-2011/06 | Chongqing | Southwest | Cross-sectional | Entertainment venues | 4 | Chinese | 318 | 34 | 10.69% |  |  |  |  |  |  |  |  |  |  |
| *b) Gorrhoeaon* |  |  |  |  |  |  |  |  |  |  |  |  |  |  |  |  |  |  |  |  |
| Liao CQ, 2011[[97](#_ENREF_97)] |  | Shanghai | East | Cross-sectional | Entertainment venues | 3 | Chinese | 400 | 1 | 1.00% |  |  |  |  |  |  |  |  |  |  |
| Wei CQ, 2007[[1](#_ENREF_1)] | 2006 |  |  | Cross-sectional | Entertainment venues | 3 | Chinese | 280 | 26 | 9.29% |  |  |  |  |  |  |  |  |  |  |
| Liu SF, 2007[[2](#_ENREF_2)] |  | Jiangsu | East | Cross-sectional | Entertainment venues | 4 | Chinese | 214 | 5 | 2.30% |  |  |  |  |  |  |  |  |  |  |
| Zhang ZX, 2009[[3](#_ENREF_3)] |  | Fujian | East | Cross-sectional | Others | 2 | Chinese | 128 | 2 | 1.56% |  |  |  |  |  |  |  |  |  |  |
| Zheng BZ, 2003[[4](#_ENREF_4)] | 2000/06 | Zhejiang | East | Cross-sectional | Entertainment venues | 5 | Chinese | 176 | 2 | 1.14% |  |  |  |  |  |  |  |  |  |  |
| Guo Y, 2010a[[98](#_ENREF_98)] | 2001 | Zhejiang | East | Cross-sectional | Detaining education center | 5 | Chinese | 331 | 77 | 23.26% |  |  |  |  |  |  |  |  |  |  |
| Du YP, 2004a[[5](#_ENREF_5)] | 2001/11 | Jiangsu | East | Cross-sectional | Entertainment venues | 5 | Chinese | 303 | 10 | 3.30% |  |  |  |  |  |  |  |  |  |  |
| Li L, 2003[[99](#_ENREF_99)] | 2001/11-2001/12 | Jiangsu | East | Cross-sectional | Entertainment venues | 4 | Chinese | 288 | 10 | 3.50% |  |  |  |  |  |  |  |  |  |  |
| Yan HJ, 2004[[6](#_ENREF_6)] | 2002/04 | Jiangsu | East | Cross-sectional | Detaining education center | 5 | Chinese | 50 | 2 | 4.00% |  |  |  |  |  |  |  |  |  |  |
| Yang P, 2005a[[7](#_ENREF_7)] | 2002/12 | Fujian | East | Cross-sectional | Entertainment venues | 3 | Chinese | 149 | 3 | 2.01% |  |  |  |  |  |  |  |  |  |  |
| Xu LL, 2006a[[8](#_ENREF_8)] | 2003/04-2004/10 | Shanghai | East | Cross-sectional | Entertainment venues | 4 | Chinese | 191 | 0 | 0.00% |  |  |  |  |  |  |  |  |  |  |
| Lin YQ, 2005[[9](#_ENREF_9)] | 2003/09 | Zhejiang | East | Cross-sectional | Detaining education center | 3 | Chinese | 181 | 13 | 7.18% |  |  |  |  |  |  |  |  |  |  |
| Wang FJ, 2008[[10](#_ENREF_10)] | 2003-2006 | Zhejiang | East | Cross-sectional | Detaining education center | 3 | Chinese | 608 | 32 | 5.26% |  |  |  |  |  |  |  |  |  |  |
| Zhang Y, 2005[[11](#_ENREF_11)] | 2004/04-2004/05 | Shandong | East | Cross-sectional | Entertainment venues | 3 | Chinese | 180 | 0 | 0.00% |  |  |  |  |  |  |  |  |  |  |
| NI CM, 2011[[12](#_ENREF_12)] | 2004-2008 | Zhejiang | East | Cross-sectional | Entertainment venues | 6 | Chinese | 405 | 40 | 9.90% |  |  |  |  |  |  |  |  |  |  |
| Zeng YB, 2009a[[13](#_ENREF_13)] | 2005 | Shanghai | East | National Sentinel Site | Detaining education center | 4 | Chinese | 448 | 19 | 4.24% |  |  |  |  |  |  |  |  |  |  |
| Qi HZ, 2007[[100](#_ENREF_100)] | 2005/01-2005/12 | Jiangsu | East | Cross-sectional | Entertainment venues | 4 | Chinese | 207 | 2 | 0.97% |  |  |  |  |  |  |  |  |  |  |
| Zeng YB, 2009b[[13](#_ENREF_13)] | 2006 | Shanghai | East | National Sentinel Site | Detaining education center | 4 | Chinese | 448 | 19 | 4.24% |  |  |  |  |  |  |  |  |  |  |
| Song SJ, 2008[[101](#_ENREF_101)] | 2006/01-2006/12 | Zhejiang | East | Cross-sectional | Entertainment venues | 3 | Chinese | 272 | 42 | 15.44% |  |  |  |  |  |  |  |  |  |  |
| Yu X, 2007[[18](#_ENREF_18)] | 2006/05 | Jiangsu | East | Cross-sectional | Entertainment venues | 5 | Chinese | 121 | 0 | 0.00% |  |  |  |  |  |  |  |  |  |  |
| Zhu FG, 2009a[[19](#_ENREF_19)] | 2006/05 | Jiangsu | East | Cross-sectional | Entertainment venues | 4 | Chinese | 252 | 39 | 15.48% |  |  |  |  |  |  |  |  |  |  |
| Zeng YB, 2009c[[13](#_ENREF_13)] | 2007 | Shanghai | East | National Sentinel Site | Detaining education center | 4 | Chinese | 448 | 9 | 2.01% |  |  |  |  |  |  |  |  |  |  |
| Wang WM, 2008[[20](#_ENREF_20)] | 2007/01-2007/12 | Jiangsu | East | Cross-sectional | Detaining education center | 4 | Chinese | 297 | 7 | 2.36% |  |  |  |  |  |  |  |  |  |  |
| Xu lq, 2008[[102](#_ENREF_102)] | 2007/06-2007/07 | Jiangsu | East | Cross-sectional | Entertainment venues | 2 | Chinese | 115 | 7 | 6.09% |  |  |  |  |  |  |  |  |  |  |
| Yang JY, 2011[[21](#_ENREF_21)] | 2007/10-2007/11 | Jiangsu | East | Cross-sectional | Entertainment venues | 4 | Chinese | 247 | 22 | 8.91% |  |  |  |  |  |  |  |  |  |  |
| Chen JQ, 2009[[22](#_ENREF_22)] | 2008/01-2008/10 | Zhejiang | East | Cross-sectional | Others | 3 | Chinese | 168 | 4 | 2.38% |  |  |  |  |  |  |  |  |  |  |
| Zhao YQ, 2010a[[23](#_ENREF_23)] | 2008/04-2008/05 | Jiangsu | East | Cross-sectional | Entertainment venues | 4 | Chinese | 396 | 2 | 0.51% |  |  |  |  |  |  |  |  |  |  |
| Wang J, 2008a[[103](#_ENREF_103)] | 2008/06 | Jiangsu | East | Cross-sectional | Entertainment venues | 4 | Chinese | 400 | 11 | 2.75% |  |  |  |  |  |  |  |  |  |  |
| Chen JQ, 2009[[104](#_ENREF_104)] | 2008/10-2008/11 | Zhejiang | East | Cross-sectional | Entertainment venues | 3 | Chinese | 111 | 2 | 1.80% |  |  |  |  |  |  |  |  |  |  |
| Wu RH, 2012[[105](#_ENREF_105)] | 2008-2011 | Shanghai | East | Cross-sectional | Detaining education center | 4 | Chinese | 422 | 49 | 11.61% |  |  |  |  |  |  |  |  |  |  |
| Wang F, 2010a[[24](#_ENREF_24)] | 2009 | Anhui | East | Cross-sectional | Entertainment venues | 4 | Chinese | 75 | 2 | 2.67% |  |  |  |  |  |  |  |  |  |  |
| Li XY, 2011[[25](#_ENREF_25)] | 2009/01-2010/05 | Zhejiang | East | Cross-sectional | Detaining education center | 3 | Chinese | 98 | 40 | 40.80% |  |  |  |  |  |  |  |  |  |  |
| Shao MC, 2010[[106](#_ENREF_106)] | 2009/04 | Jiangsu | East | Cross-sectional | Entertainment venues | 1 | Chinese | 402 | 1 | 0.25% |  |  |  |  |  |  |  |  |  |  |
| Zhang QQ, 2012[[107](#_ENREF_107)] | 2009/07-2009/09 | Jiangsu | East | Cross-sectional | Entertainment venues | 4 | Chinese | 1,806 | 46 | 2.55% |  |  |  |  |  |  |  |  |  |  |
| Wei ZY, 2010a[[27](#_ENREF_27)] | 2009/07-2009/09 | Jiangsu | East | Cross-sectional | Entertainment venues | 5 | Chinese | 940 | 36 | 3.83% |  |  |  |  |  |  |  |  |  |  |
| Zhang QQ, 2012[[107](#_ENREF_107)] | 2009/10-2009/12 | Jiangsu | East | Cross-sectional | Entertainment venues | 4 | Chinese | 737 | 15 | 4.20% |  |  |  |  |  |  |  |  |  |  |
| Tang ZL, 2011[[28](#_ENREF_28)] | 2010/03-2010/07 | Shandong | East | Cross-sectional | Entertainment venues | 4 | Chinese | 106 | 29 | 27.40% |  |  |  |  |  |  |  |  |  |  |
| Tang ZL, 2011[[28](#_ENREF_28)] | 2010/03-2010/07 | Shandong | East | Cross-sectional | Entertainment venues | 4 | Chinese | 216 | 30 | 13.90% |  |  |  |  |  |  |  |  |  |  |
| Li DL, 2006a[[29](#_ENREF_29)] | 2001 | Beijing | North | Cross-sectional | National sentinel site | 4 | Chinese | 354 | 64 | 18.82% |  |  |  |  |  |  |  |  |  |  |
| Zhao YR, 2003[[30](#_ENREF_30)] | 2001/07-2002/07 | Beijing | North | Cross-sectional | Detaining education center | 6 | Chinese | 510 | 53 | 10.39% |  |  |  |  |  |  |  |  |  |  |
| Li DL, 2006b[[29](#_ENREF_29)] | 2002 | Beijing | North | Cross-sectional | National sentinel site | 4 | Chinese | 571 | 25 | 4.89% |  |  |  |  |  |  |  |  |  |  |
| Li DL, 2006c[[29](#_ENREF_29)] | 2003 | Beijing | North | Cross-sectional | National sentinel site | 4 | Chinese | 174 | 2 | 1.46% |  |  |  |  |  |  |  |  |  |  |
| Li DL, 2006d[[29](#_ENREF_29)] | 2004 | Beijing | North | Cross-sectional | National sentinel site | 4 | Chinese | 413 | 28 | 6.78% |  |  |  |  |  |  |  |  |  |  |
| Li JE, 2012[[108](#_ENREF_108)] | 2008/03 | Hebei | North | Cross-sectional | Entertainment venues | 2 | Chinese | 370 | 6 | 1.62% |  |  |  |  |  |  |  |  |  |  |
| Zhang XY, 2012[[109](#_ENREF_109)] | 2010/12-2011/12 | Inner Mongolia | North | Cross-sectional | Entertainment venues | 4 | Chinese | 247 | 9 | 3.60% |  |  |  |  |  |  |  |  |  |  |
| Li Y, 2011[[31](#_ENREF_31)] | 2010 | Heilongjiang | Northeast | Cross-sectional | National sentinel site | 4 | Chinese | 7,662 | 112 | 1.59% |  |  |  |  |  |  |  |  |  |  |
| Lin L, 2002b[[110](#_ENREF_110)] | 2000/08-2000/11 | Xinjiang | Northwest | Cross-sectional | Entertainment venues | 4 | Chinese | 81 | 2 | 2.47% |  |  |  |  |  |  |  |  |  |  |
| Wei XW, 2002[[111](#_ENREF_111)] | 2001/07-2001/08 | Xinjiang | Northwest | Cross-sectional | Entertainment venues | 3 | Chinese | 181 | 26 | 14.36% |  |  |  |  |  |  |  |  |  |  |
| Lu H, 2003[[32](#_ENREF_32)] | 2002 | Xinjiang | Northwest | Cross-sectional | Detaining education center | 2 | Chinese | 346 | 22 | 0.64% |  |  |  |  |  |  |  |  |  |  |
| Xi SH, 2004[[33](#_ENREF_33)] | 2002/05-2002/07 | Gansu | Northwest | Cross-sectional | Entertainment venues | 7 | Chinese | 88 | 3 | 3.41% |  |  |  |  |  |  |  |  |  |  |
| Wei XW, 2005[[34](#_ENREF_34)] | 2003/03-2003/05 | Xinjiang | Northwest | Cross-sectional | Entertainment venues | 2 | Chinese | 459 | 32 | 6.97% |  |  |  |  |  |  |  |  |  |  |
| Li F, 2006[[35](#_ENREF_35)] | 2004/04-2004/06 | Xinjiang | Northwest | Cross-sectional | Entertainment venues | 6 | Chinese | 611 | 11 | 1.80% |  |  |  |  |  |  |  |  |  |  |
| Wang HZ, 2006[[36](#_ENREF_36)] | 2005/06 | Xinjiang | Northwest | Cross-sectional | Entertainment venues | 3 | Chinese | 366 | 6 | 1.64% |  |  |  |  |  |  |  |  |  |  |
| Song Y, 2006[[37](#_ENREF_37)] | 2006 | Xinjiang | Northwest | Cross-sectional | Entertainment venues | 4 | Chinese | 103 | 8 | 7.77% |  |  |  |  |  |  |  |  |  |  |
| Yang SP, 2008a[[38](#_ENREF_38)] | 2007/08-2007/09 | Xinjiang | Northwest | Cross-sectional | Entertainment venues | 3 | Chinese | 251 | 26 | 10.36% |  |  |  |  |  |  |  |  |  |  |
| Gao W, 2012 (a)[[40](#_ENREF_40)] | 2008/11-2009/01 | Gansu | Northwest | Cross-sectional | Entertainment venues | 7 | English | 350 | 30 | 8.57% |  |  |  |  |  |  |  |  |  |  |
| Zhang CJ, 2001[[41](#_ENREF_41)] | 2000/03-2000/08 | Henan | South Central | Cross-sectional | Detaining education center | 3 | Chinese | 65 | 5 | 7.69% |  |  |  |  |  |  |  |  |  |  |
| Sun L, 2003[[112](#_ENREF_112)] | 2000/06 | Henan | South Central |  | Entertainment venues | 3 | Chinese | 35 | 1 | 2.86% |  |  |  |  |  |  |  |  |  |  |
| Sun J, 2003[[113](#_ENREF_113)] | 2001/09-2001/12 | Henan | South Central | Cross-sectional | Entertainment venues | 2 | Chinese | 220 | 20 | 9.09% |  |  |  |  |  |  |  |  |  |  |
| Hu B, 2004a[[43](#_ENREF_43)] | 2002 | Hainan | South Central | Cross-sectional | Entertainment venues | 5 | Chinese | 420 | 15 | 3.57% |  |  |  |  |  |  |  |  |  |  |
| Peng SQ, 2004[[114](#_ENREF_114)] | 2002/08-10 | Guangdong | South Central | Cross-sectional | Detaining education center | 4 | Chinese | 80 | 11 | 13.75% |  |  |  |  |  |  |  |  |  |  |
| Liang YW, 2003[[44](#_ENREF_44)] | 2002/09 | Hainan | South Central | Cross-sectional | Entertainment venues | 0 | Chinese | 62 | 9 | 14.52% |  |  |  |  |  |  |  |  |  |  |
| He QY, 2003[[45](#_ENREF_45)] | 2002/09 | Hainan | South Central | Cross-sectional | Entertainment venues | 5 | Chinese | 417 | 15 | 3.60% |  |  |  |  |  |  |  |  |  |  |
| Liang BH, 2008[[115](#_ENREF_115)] | 2002-2005 | Guangdong | South Central | Cross-sectional | Detaining education center | 5 | Chinese | 1,508 | 15 | 0.99% |  |  |  |  |  |  |  |  |  |  |
| Fan XJ, 2007a[[116](#_ENREF_116)] | 2003 | Hunan | South Central | Cross-sectional | Entertainment venues | 5 | Chinese | 106 | 15 | 14.10% |  |  |  |  |  |  |  |  |  |  |
| Luo J, 2005c[[46](#_ENREF_46)] | 2003/06 | Guangxi | South Central | Cross-sectional | Entertainment venues | 4 | Chinese | 126 | 5 | 3.96% |  |  |  |  |  |  |  |  |  |  |
| Li MQ, 2005a[[117](#_ENREF_117)] | 2003/06 | Guangxi | South Central | Cross-sectional | Entertainment venues | 4 | Chinese | 278 | 24 | 8.60% |  |  |  |  |  |  |  |  |  |  |
| Luo J, 2005a[[46](#_ENREF_46)] | 2003/06 | Guangxi | South Central | Cross-sectional | Entertainment venues | 4 | Chinese | 278 | 24 | 8.63% |  |  |  |  |  |  |  |  |  |  |
| Luo J, 2005e[[46](#_ENREF_46)] | 2003/06 | Guangxi | South Central | Cross-sectional | Entertainment venues | 4 | Chinese | 152 | 19 | 12.50% |  |  |  |  |  |  |  |  |  |  |
| Tang GB, 2007a[[48](#_ENREF_48)] | 2004 | Hunan | South Central | Cross-sectional | Entertainment venues | 4 | Chinese | 397 | 11 | 2.77% |  |  |  |  |  |  |  |  |  |  |
| Hong Y, 2009[[50](#_ENREF_50)] | 2004 | Guangxi | South Central | Cross-sectional | Entertainment venues | 4 | English | 411 | 68 | 16.50% |  |  |  |  |  |  |  |  |  |  |
| Ren Y, 2007[[118](#_ENREF_118)] | 2004 | Guangdong | South Central | National sentinel site | Detaining education center | 6 | Chinese | 518 | 3 | 0.58% |  |  |  |  |  |  |  |  |  |  |
| Xiong CB, 2006a[[49](#_ENREF_49)] | 2004 | Hunan | South Central | Cross-sectional | Entertainment venues | 4 | Chinese | 136 | 5 | 3.68% |  |  |  |  |  |  |  |  |  |  |
| Wang H, 2005[[51](#_ENREF_51)] | 2004/03-2004/05 | Guangxi | South Central | Cross-sectional | Entertainment venues | 4 | English | 410 | 67 | 16.40% |  |  |  |  |  |  |  |  |  |  |
| Zhou YJ, 2007[[52](#_ENREF_52)] | 2004/05 | Guangxi | South Central | Cross-sectional | Entertainment venues | 6 | Chinese | 80 | 17 | 21.25% |  |  |  |  |  |  |  |  |  |  |
| Sun CX, 2009a[[119](#_ENREF_119)] | 2004/07 | Hubei | South Central | Cross-sectional | Entertainment venues | 3 | Chinese | 59 | 7 | 11.86% |  |  |  |  |  |  |  |  |  |  |
| Wu MS, 2006[[56](#_ENREF_56)] | 2005/03 | Guangxi | South Central | Cross-sectional | Entertainment venues | 2 | Chinese | 187 | 16 | 8.56% |  |  |  |  |  |  |  |  |  |  |
| Zhang JL, 2008a[[57](#_ENREF_57)] | 2006/01-2007/05 | Guangxi | South Central | Cross-sectional | Entertainment venues | 4 | Chinese | 103 | 1 | 0.97% |  |  |  |  |  |  |  |  |  |  |
| Li CG, 2007a[[58](#_ENREF_58)] | 2006/05 | Guangdong | South Central | Cross-sectional | Entertainment venues | 6 | Chinese | 522 | 81 | 15.52% |  |  |  |  |  |  |  |  |  |  |
| Li Y, 2012[[59](#_ENREF_59)] | 2006/08 - 2007/01 | Guangdong | South Central | Cross-sectional | Entertainment venues | 6 | English | 318 | 27 | 8.50% |  |  |  |  |  |  |  |  |  |  |
| Li Y, 2009[[60](#_ENREF_60)] | 2006/08-2007/01 | Guangdong | South Central | Cross-sectional | Entertainment venues | 6 | Chinese | 320 | 30 | 9.38% |  |  |  |  |  |  |  |  |  |  |
| Wu ZZ, 2008[[61](#_ENREF_61)] | 2006/11-2007/01 | Guangdong | South Central | Cross-sectional | Entertainment venues | 1 | Chinese | 213 | 7 | 3.29% |  |  |  |  |  |  |  |  |  |  |
| Zhang SJ, 2008i[[120](#_ENREF_120)] | 2007 | Guangxi | South Central | Cross-sectional | VCT | 4 | Chinese | 385 | 8 | 2.08% |  |  |  |  |  |  |  |  |  |  |
| Li XF, 2008a[[121](#_ENREF_121)] | 2007/01-2007/03 | Hunan | South Central | Cross-sectional | Entertainment venues | 4 | Chinese | 142 | 5 | 2.10% |  |  |  |  |  |  |  |  |  |  |
| Zhu BY, 2012[[62](#_ENREF_62)] | 2007/05-2007/10 | Guangxi | South Central | Cross-sectional | Entertainment venues | 6 | English | 488 | 9 | 1.84% |  |  |  |  |  |  |  |  |  |  |
| Yi QM, 2009a[[64](#_ENREF_64)] | 2008/09 | Henan | South Central | Cross-sectional | Entertainment venues | 3 | Chinese | 300 | 33 | 11.00% |  |  |  |  |  |  |  |  |  |  |
| Yi QM, 2009b[[64](#_ENREF_64)] | 2008/09 | Henan | South Central | Cross-sectional | Entertainment venues | 3 | Chinese | 88 | 7 | 8.00% |  |  |  |  |  |  |  |  |  |  |
| Yi QM, 2009c[[64](#_ENREF_64)] | 2008/09 | Henan | South Central | Cross-sectional | Entertainment venues | 3 | Chinese | 85 | 10 | 11.76% |  |  |  |  |  |  |  |  |  |  |
| Yi QM, 2009d[[64](#_ENREF_64)] | 2008/09 | Henan | South Central | Cross-sectional | Entertainment venues | 3 | Chinese | 127 | 16 | 12.60% |  |  |  |  |  |  |  |  |  |  |
| Zhao YY, 2010[[65](#_ENREF_65)] | 2008/10-2008/12 | Guangdong | South Central | Cross-sectional | Entertainment venues | 4 | Chinese | 622 | 21 | 3.38% |  |  |  |  |  |  |  |  |  |  |
| Xiang SB, 2010b[[66](#_ENREF_66)] | 2009 | Hunan | South Central | Cross-sectional | Entertainment venues | 4 | Chinese | 401 | 31 | 7.73% |  |  |  |  |  |  |  |  |  |  |
| Zhong J, 2011[[67](#_ENREF_67)] | 2009/06-2009/08 | Guangxi | South Central | Cross-sectional | Entertainment venues | 4 | Chinese | 1,166 | 31 | 2.66% |  |  |  |  |  |  |  |  |  |  |
| Zhong J, 2011[[67](#_ENREF_67)] | 2009/06-2009/08 | Guangxi | South Central | Cross-sectional | Entertainment venues | 4 | Chinese | 1,166 | 31 | 2.66% |  |  |  |  |  |  |  |  |  |  |
| Zhong J, 2010a[[68](#_ENREF_68)] | 2009/06-2009/08 | Guangxi | South Central | Cross-sectional | Entertainment venues | 5 | Chinese | 406 | 31 | 7.64% |  |  |  |  |  |  |  |  |  |  |
| Zhong J, 2010c[[68](#_ENREF_68)] | 2009/06-2009/08 | Guangxi | South Central | Cross-sectional | Entertainment venues | 5 | Chinese | 80 | 1 | 1.25% |  |  |  |  |  |  |  |  |  |  |
| Zhong J, 2010d[[68](#_ENREF_68)] | 2009/06-2009/08 | Guangxi | South Central | Cross-sectional | Entertainment venues | 5 | Chinese | 165 | 9 | 5.45% |  |  |  |  |  |  |  |  |  |  |
| Zhong J, 2010e[[68](#_ENREF_68)] | 2009/06-2009/08 | Guangxi | South Central | Cross-sectional | Entertainment venues | 5 | Chinese | 161 | 21 | 13.04% |  |  |  |  |  |  |  |  |  |  |
| Zhao JZ, 2011[[69](#_ENREF_69)] | 2009/07-2009/12 | Hubei | South Central | Cross-sectional | Entertainment venues | 4 | Chinese | 149 | 6 | 4.00% |  |  |  |  |  |  |  |  |  |  |
| Liang HX, 2011[[122](#_ENREF_122)] | 2009-2010 | Hubei | South Central | Cross-sectional | Others | 4 | Chinese | 800 | 43 | 5.38% |  |  |  |  |  |  |  |  |  |  |
| Wang WP, 2008[[123](#_ENREF_123)] | N/A | Hunan | South Central | Cross-sectional | Entertainment venues | 3 | Chinese | 625 | 72 | 11.52% |  |  |  |  |  |  |  |  |  |  |
| Chen SH, 2005[[124](#_ENREF_124)] | N/A | Guangdong | South Central | Cross-sectional | Entertainment venues | 3 | Chinese | 271 | 4 | 1.50% |  |  |  |  |  |  |  |  |  |  |
| Chen ZH, 2007a[[71](#_ENREF_71)] | N/A | Guangdong | South Central | Cross-sectional | Entertainment venues | 3 | Chinese | 1,200 | 44 | 3.67% |  |  |  |  |  |  |  |  |  |  |
| Yang P, 2006[[72](#_ENREF_72)] |  | Sichuan | Southwest | Cross-sectional | Entertainment venues | 2 | Chinese | 407 | 100 | 24.57% |  |  |  |  |  |  |  |  |  |  |
| Wu JW, 2002[[125](#_ENREF_125)] | 2000/10-2000/11 | Chongqing | Southwest | National Sentinel Site | Entertainment venues | 4 | Chinese | 103 | 16 | 15.53% |  |  |  |  |  |  |  |  |  |  |
| Wu Y, 2002a[[73](#_ENREF_73)] | 2001/09 | Chongqing | Southwest | Cohort | Entertainment venues | 6 | Chinese | 184 | 44 | 23.91% |  |  |  |  |  |  |  |  |  |  |
| Wu Y, 2002b[[73](#_ENREF_73)] | 2001/09 | Chongqing | Southwest | Cohort | Detaining education center | 6 | Chinese | 195 | 69 | 35.38% |  |  |  |  |  |  |  |  |  |  |
| Wang QQ, 2005[[74](#_ENREF_74)] | 2004/02 | Sichuan | Southwest | Cross-sectional | Entertainment venues | 3 | Chinese | 206 | 49 | 23.79% |  |  |  |  |  |  |  |  |  |  |
| Wu Y, 2007a[[75](#_ENREF_75)] | 2004/09 | Chongqing | Southwest | Cohort | Entertainment venues | 4 | Chinese | 157 | 9 | 5.73% |  |  |  |  |  |  |  |  |  |  |
| Wu Y, 2007b[[75](#_ENREF_75)] | 2004/09 | Chongqing | Southwest | Cohort | Entertainment venues | 4 | Chinese | 169 | 2 | 1.18% |  |  |  |  |  |  |  |  |  |  |
| Li YY, 2009a[[126](#_ENREF_126)] | 2004/10-2006/12 | Yunnan | Southwest | Cross-sectional | Entertainment venues | 4 | Chinese | 100 | 1 | 1.00% |  |  |  |  |  |  |  |  |  |  |
| Ruan Y, 2006[[127](#_ENREF_127)] | 2004/12-2005/01 | Sichuan | Southwest | Cross-sectional | Entertainment venues | 5 | English | 343 | 7 | 2.04% |  |  |  |  |  |  |  |  |  |  |
| Cao XY, 2006[[128](#_ENREF_128)] | 2004/12-2005/01 | Sichuan | Southwest | Cross-sectional | Entertainment venues | 5 | Chinese | 203 | 7 | 3.45% |  |  |  |  |  |  |  |  |  |  |
| Dai XQ, 2007[[76](#_ENREF_76)] | 2005/06-2005/09 | Chongqing | Southwest | Cross-sectional | Entertainment venues | 5 | Chinese | 341 | 65 | 21.50% |  |  |  |  |  |  |  |  |  |  |
| Ding XB, 2006[[78](#_ENREF_78)] | 2005/08-2005/09 | Chongqing | Southwest | Cross-sectional | Entertainment venues | 4 | Chinese | 519 | 16 | 3.08% |  |  |  |  |  |  |  |  |  |  |
| Tan XJ, 2007[[77](#_ENREF_77)] | 2005/08-2005/09 | Chongqing | Southwest | Cross-sectional | Entertainment venues | 5 | Chinese | 266 | 5 | 1.88% |  |  |  |  |  |  |  |  |  |  |
| Wang HB, 2010a[[79](#_ENREF_79)] | 2006/03 | Yunnan | Southwest | Cross-sectional | Entertainment venues | 7 | English | 737 | 61 | 8.30% |  |  |  |  |  |  |  |  |  |  |
| Xu J, 2011[[80](#_ENREF_80)] | 2006/03 - 2007/04 | Yunnan | Southwest | Cohort | Others | 3 | English | 1,642 | 170 | 10.35% |  |  |  |  |  |  |  |  |  |  |
| Wang H, 2012[[81](#_ENREF_81)] | 2006/03 - 2009/11 | Yunnan | Southwest | Cohort | Entertainment venues | 6 | English | 851 | 58 | 6.80% |  |  |  |  |  |  |  |  |  |  |
| Li QH, 2010a[[82](#_ENREF_82)] | 2006/03-2006/04 | Yunnan | Southwest | Cross-sectional | Entertainment venues | 6 | Chinese | 716 | 59 | 8.20% |  |  |  |  |  |  |  |  |  |  |
| Xu JJ, 2008[[83](#_ENREF_83)] | 2006/03-2006/04 | Yunnan | Southwest | Cross-sectional | Entertainment venues | 6 | English | 95 | 35 | 36.80% |  |  |  |  |  |  |  |  |  |  |
| Wang H, 2009[[84](#_ENREF_84)] | 2006/03-2006/05 | Yunnan | Southwest | Cross-sectional | Entertainment venues | 4 | English | 737 | 61 | 8.28% |  |  |  |  |  |  |  |  |  |  |
| Wang H, 2009e[[84](#_ENREF_84)] | 2006/03-2006/05 | Yunnan | Southwest | Cross-sectional | Entertainment venues | 4 | English | 458 | 33 | 7.20% |  |  |  |  |  |  |  |  |  |  |
| Wang H, 2009f[[84](#_ENREF_84)] | 2006/03-2006/05 | Yunnan | Southwest | Cross-sectional | Entertainment venues | 4 | English | 279 | 28 | 10.00% |  |  |  |  |  |  |  |  |  |  |
| Wang HB, 2010a[[79](#_ENREF_79)] | 2006/03-2006/12 | Yunnan | Southwest | Cross-sectional | Entertainment venues | 6 | English | 705 | 39 | 5.53% |  |  |  |  |  |  |  |  |  |  |
| Wang H, 2011[[85](#_ENREF_85)] | 2006/03-2009/11 | Yunnan | Southwest | Cohort | Entertainment venues | 6 | English | 741 | 54 | 7.30% |  |  |  |  |  |  |  |  |  |  |
| Wang H, 2011[[86](#_ENREF_86)] | 2006/03-2009/11 | Yunnan | Southwest | Cohort | Entertainment venues | 4 | English | 1,832 | 154 | 8.41% |  |  |  |  |  |  |  |  |  |  |
| Luo XR, 2008a[[87](#_ENREF_87)] | 2006/05-2006/06 | Sichuan | Southwest | Cross-sectional | Entertainment venues | 5 | Chinese | 194 | 16 | 8.25% |  |  |  |  |  |  |  |  |  |  |
| Li QH, 2009a[[88](#_ENREF_88)] | 2006/09-2006/10 | Yunnan | Southwest | Cross-sectional | Entertainment venues | 7 | Chinese | 335 | 10 | 2.99% |  |  |  |  |  |  |  |  |  |  |
| Li QH, 2009b[[88](#_ENREF_88)] | 2006/09-2006/10 | Yunnan | Southwest | Cross-sectional | Entertainment venues | 7 | Chinese | 312 | 20 | 6.11% |  |  |  |  |  |  |  |  |  |  |
| Li QH, 2009c[[88](#_ENREF_88)] | 2006/09-2006/10 | Yunnan | Southwest | Cross-sectional | Entertainment venues | 7 | Chinese | 87 | 13 | 14.94% |  |  |  |  |  |  |  |  |  |  |
| Wang GX, 2008a[[89](#_ENREF_89)] | 2006/09-2006/10 | Yunnan | Southwest | Cross-sectional | Entertainment venues | 5 | Chinese | 734 | 43 | 5.86% |  |  |  |  |  |  |  |  |  |  |
| Wang GX, 2008b[[89](#_ENREF_89)] | 2006/09-2006/10 | Yunnan | Southwest | Cross-sectional | Entertainment venues | 5 | Chinese | 266 | 14 | 5.26% |  |  |  |  |  |  |  |  |  |  |
| Wang GX, 2008c[[89](#_ENREF_89)] | 2006/09-2006/10 | Yunnan | Southwest | Cross-sectional | Entertainment venues | 5 | Chinese | 351 | 31 | 8.83% |  |  |  |  |  |  |  |  |  |  |
| Wang GX, 2008d[[89](#_ENREF_89)] | 2006/09-2006/10 | Yunnan | Southwest | Cross-sectional | Entertainment venues | 5 | Chinese | 120 | 16 | 13.33% |  |  |  |  |  |  |  |  |  |  |
| Wang HB, 2010b[[79](#_ENREF_79)] | 2006/10 | Yunnan | Southwest | Cross-sectional | Entertainment venues | 3 | English | 747 | 43 | 5.80% |  |  |  |  |  |  |  |  |  |  |
| Gao LL, 2008a[[90](#_ENREF_90)] | 2007 | Yunnan | Southwest | Cross-sectional | Detaining education center | 4 | Chinese | 270 | 1 | 0.37% |  |  |  |  |  |  |  |  |  |  |
| Yang X, 2009a[[129](#_ENREF_129)] | 2007 | Yunnan | Southwest | Cross-sectional | Entertainment venues | 1 | Chinese | 371 | 13 | 3.50% |  |  |  |  |  |  |  |  |  |  |
| Yang X, 2009f[[129](#_ENREF_129)] | 2007 | Yunnan | Southwest | Cross-sectional | Entertainment venues | 1 | Chinese | 211 | 7 | 3.30% |  |  |  |  |  |  |  |  |  |  |
| Yang X, 2009k[[129](#_ENREF_129)] | 2007 | Yunnan | Southwest | Cross-sectional | Entertainment venues | 1 | Chinese | 109 | 1 | 0.90% |  |  |  |  |  |  |  |  |  |  |
| Yang X, 2009p[[129](#_ENREF_129)] | 2007 | Yunnan | Southwest | Cross-sectional | Entertainment venues | 1 | Chinese | 51 | 5 | 9.80% |  |  |  |  |  |  |  |  |  |  |
| Xu JJ, 2012[[91](#_ENREF_91)] | 2007/03-2007-07 | Yunnan | Southwest | Cross-sectional | Entertainment venues | 6 | English | 705 | 40 | 5.60% |  |  |  |  |  |  |  |  |  |  |
| Wang HB, 2010c[[79](#_ENREF_79)] | 2007/05 | Yunnan | Southwest | Cross-sectional | Entertainment venues | 4 | English | 705 | 39 | 5.50% |  |  |  |  |  |  |  |  |  |  |
| Wang HB, 2010b[[79](#_ENREF_79)] | 2007/05 | Yunnan | Southwest |  | Others | 6 | English | 705 | 39 | 5.53% |  |  |  |  |  |  |  |  |  |  |
| Yao Y, 2012[[92](#_ENREF_92)] | 2007/09-2007/10 | Yunnan | Southwest | Cross-sectional | Entertainment venues | 7 | English | 373 | 25 | 6.70% |  |  |  |  |  |  |  |  |  |  |
| Pan HJ, 2011[[130](#_ENREF_130)] | 2008/01-2009/07 | Yunnan | Southwest | Cross-sectional | Entertainment venues | 3 | Chinese | 152 | 3 | 1.97% |  |  |  |  |  |  |  |  |  |  |
| Jin X, 2011[[93](#_ENREF_93)] | 2008/04 - 2008/05 | Yunnan | Southwest | Cross-sectional | Entertainment venues | 7 | English | 568 | 47 | 8.30% |  |  |  |  |  |  |  |  |  |  |
| Jin X, 2011[[93](#_ENREF_93)] | 2008/04-2008/05 | Yunnan | Southwest | Cross-sectional | Entertainment venues | 7 | English | 568 | 47 | 8.27% |  |  |  |  |  |  |  |  |  |  |
| Zhang XD, 2012[[95](#_ENREF_95)] | 2010/07-2011/02 | Yunnan | Southwest | Cross-sectional | Entertainment venues | 6 | English | 191 | 25 | 13.10% |  |  |  |  |  |  |  |  |  |  |
| Shao ZP, 2012[[96](#_ENREF_96)] | 2011/05-2011/06 | Chongqing | Southwest | Cross-sectional | Entertainment venues | 4 | Chinese | 318 | 11 | 3.46% |  |  |  |  |  |  |  |  |  |  |
| c) Syphilis |  |  |  |  |  |  |  |  |  |  |  |  |  |  |  |  |  |  |  |  |
| Wei CQ, 2007[[1](#_ENREF_1)] | 2006 |  |  | Cross-sectional | Entertainment venues | 3 | Chinese | 280 | 48 | 17.14% |  |  |  |  |  |  |  |  |  |  |
| Zhang YH, 2011[[131](#_ENREF_131)] | 2008/04-2008/07 |  |  | Cross-sectional | Others | 2 | Chinese | 8,717 | 242 | 2.80% |  |  |  |  |  |  |  |  |  |  |
| Chen XS, 2012 (a)[[132](#_ENREF_132)] | 2009/06-2009/09 |  |  | Cross-sectional | Entertainment venues | 7 | English | 5,322 | 291 | 5.47% |  |  |  |  |  |  |  |  |  |  |
| Zhang H, 2011[[133](#_ENREF_133)] | 2010 | Fujian | East | Cross-sectional | Entertainment venues | 4 | Chinese | 479 | 1 | 0.21% |  |  |  |  |  |  |  |  |  |  |
| Liao CQ, 2011[[97](#_ENREF_97)] |  | Shanghai | East | Cross-sectional | Entertainment venues | 3 | Chinese | 401 | 22 | 5.50% |  |  |  |  |  |  |  |  |  |  |
| Qin QR, 2011[[134](#_ENREF_134)] |  | Anhui | East | Cross-sectional | Entertainment venues | 4 | Chinese | 182 | 32 | 17.60% |  |  |  |  |  |  |  |  |  |  |
| Zhang XC, 2011[[135](#_ENREF_135)] |  | Zhejiang | East | Cross-sectional | Entertainment venues | 2 | Chinese | 823 | 17 | 2.07% |  |  |  |  |  |  |  |  |  |  |
| Li HX, 2011[[136](#_ENREF_136)] |  | Jiangsu | East | Cross-sectional | Entertainment venues | 3 | Chinese | 58 | 11 | 18.90% |  |  |  |  |  |  |  |  |  |  |
| Zheng BZ, 2003[[4](#_ENREF_4)] | 2000/06 | Zhejiang | East | Cross-sectional | Entertainment venues | 5 | Chinese | 176 | 2 | 1.14% |  |  |  |  |  |  |  |  |  |  |
| Xie HY, 2004[[137](#_ENREF_137)] | 2000-2003 | Fujian | East | National Sentinel Site | Detaining education center | 7 | Chinese | 1,996 | 353 | 17.70% |  |  |  |  |  |  |  |  |  |  |
| Hua YZ, 2006a[[138](#_ENREF_138)] | 2001 | Jiangsu | East | Cross-sectional | Detaining education center | 3 | Chinese | 38 | 1 | 2.63% |  |  |  |  |  |  |  |  |  |  |
| Guo Y, 2010a[[98](#_ENREF_98)] | 2001 | Zhejiang | East | Cross-sectional | Detaining education center | 5 | Chinese | 331 | 42 | 12.70% |  |  |  |  |  |  |  |  |  |  |
| Chen YL, 2009b[[139](#_ENREF_139)] | 2001 | Fujian | East | Cross-sectional | Detaining education center | 5 | Chinese | 267 | 16 | 6.00% |  |  |  |  |  |  |  |  |  |  |
| Chen YL, 2009a[[139](#_ENREF_139)] | 2001-2007 | Fujian | East | Cross-sectional | Detaining education center | 5 | Chinese | 1,859 | 193 | 10.38% |  |  |  |  |  |  |  |  |  |  |
| Wang BF, 2004[[140](#_ENREF_140)] | 2002 | Fujian | East | Cross-sectional | Detaining education center | 5 | Chinese | 267 | 55 | 20.60% |  |  |  |  |  |  |  |  |  |  |
| Chen YL, 2009c[[139](#_ENREF_139)] | 2002 | Fujian | East | Cross-sectional | Detaining education center | 5 | Chinese | 267 | 16 | 6.00% |  |  |  |  |  |  |  |  |  |  |
| Yan HJ, 2004[[6](#_ENREF_6)] | 2002/04 | Jiangsu | East | Cross-sectional | Detaining education center | 5 | Chinese | 90 | 28 | 31.10% |  |  |  |  |  |  |  |  |  |  |
| Xie Y, 2006[[141](#_ENREF_141)] | 2002-2004 | Fujian | East | Cross-sectional | Detaining education center | 4 | Chinese | 532 | 120 | 22.60% |  |  |  |  |  |  |  |  |  |  |
| Chen YL, 2009d[[139](#_ENREF_139)] | 2003 | Fujian | East | Cross-sectional | Entertainment venues | 5 | Chinese | 252 | 41 | 16.27% |  |  |  |  |  |  |  |  |  |  |
| Xu LL, 2006a[[8](#_ENREF_8)] | 2003/04-2004/10 | Shanghai | East | Cross-sectional | Entertainment venues | 4 | Chinese | 191 | 6 | 2.70% |  |  |  |  |  |  |  |  |  |  |
| Li XF, 2006a[[142](#_ENREF_142)] | 2003/08-2003/10 & 2004/05-2004/07 | Shandong | East | Cross-sectional | Entertainment venues | 7 | Chinese | 428 | 15 | 3.50% |  |  |  |  |  |  |  |  |  |  |
| Lin YQ, 2005[[9](#_ENREF_9)] | 2003/09 | Zhejiang | East | Cross-sectional | Detaining education center | 6 | Chinese | 181 | 20 | 11.05% |  |  |  |  |  |  |  |  |  |  |
| Wang FJ, 2008[[10](#_ENREF_10)] | 2003-2006 | Zhejiang | East | Cross-sectional | Detaining education center | 4 | Chinese | 608 | 38 | 6.25% |  |  |  |  |  |  |  |  |  |  |
| Chen YL, 2009e[[139](#_ENREF_139)] | 2004 | Fujian | East | Cross-sectional | Detaining education center | 5 | Chinese | 254 | 43 | 16.93% |  |  |  |  |  |  |  |  |  |  |
| Wang HY, 2005[[143](#_ENREF_143)] | 2004/03-2004/12 | Shandong | East | Cross-sectional | Others | 4 | Chinese | 456 | 48 | 10.53% |  |  |  |  |  |  |  |  |  |  |
| Zheng H, 2012[[144](#_ENREF_144)] | 2004/07-2004/08 | Jiangsu | East | Cross-sectional | Entertainment venues | 3 | Chinese | 105 | 0 | 0.00% |  |  |  |  |  |  |  |  |  |  |
| NI CM, 2011[[12](#_ENREF_12)] | 2004-2008 | Zhejiang | East | Cross-sectional | Entertainment venues | 6 | Chinese | 405 | 12 | 3.00% |  |  |  |  |  |  |  |  |  |  |
| Yang MX, 2009[[145](#_ENREF_145)] | 2005 | Shanghai | East | Cross-sectional | Entertainment venues | 4 | Chinese | 79 | 5 | 6.30% |  |  |  |  |  |  |  |  |  |  |
| Zeng YB, 2009a[[13](#_ENREF_13)] | 2005 | Shanghai | East | National Sentinel Site | Detaining education center | 4 | Chinese | 448 | 16 | 3.57% |  |  |  |  |  |  |  |  |  |  |
| Chen YL, 2009f[[139](#_ENREF_139)] | 2005 | Fujian | East | Cross-sectional | Entertainment venues | 5 | Chinese | 345 | 24 | 7.83% |  |  |  |  |  |  |  |  |  |  |
| Qi HZ, 2007[[100](#_ENREF_100)] | 2005/01-2005/12 | Jiangsu | East | Cross-sectional | Entertainment venues | 4 | Chinese | 207 | 11 | 5.31% |  |  |  |  |  |  |  |  |  |  |
| Yu WX, 2006[[146](#_ENREF_146)] | 2005/07-2005/09 | Jiangsu | East | Cross-sectional | Entertainment venues | 4 | Chinese | 258 | 20 | 7.80% |  |  |  |  |  |  |  |  |  |  |
| Wang Y, 2007[[147](#_ENREF_147)] | 2005/08-2005/10 | Anhui | East | Cross-sectional | Entertainment venues | 5 | Chinese | 377 | 13 | 3.40% |  |  |  |  |  |  |  |  |  |  |
| Hong H, 2010a[[148](#_ENREF_148)] | 2006 | Zhejiang | East | Cross-sectional | Others | 2 | English | 403 | 54 | 13.40% |  |  |  |  |  |  |  |  |  |  |
| Huang Y, 2012[[149](#_ENREF_149)] | 2006 | Anhui | East | Cross-sectional | Entertainment venues | 4 | Chinese | 384 | 12 | 3.13% |  |  |  |  |  |  |  |  |  |  |
| Zeng YB, 2009b[[13](#_ENREF_13)] | 2006 | Shanghai | East | National Sentinel Site | Detaining education center | 4 | Chinese | 448 | 26 | 5.80% |  |  |  |  |  |  |  |  |  |  |
| Chen YL, 2009g[[139](#_ENREF_139)] | 2006 | Fujian | East | Cross-sectional | Entertainment venues | 5 | Chinese | 251 | 27 | 9.56% |  |  |  |  |  |  |  |  |  |  |
| Song SJ, 2008[[101](#_ENREF_101)] | 2006/01-2006/12 | Zhejiang | East | Cross-sectional | Entertainment venues | 3 | Chinese | 272 | 69 | 25.62% |  |  |  |  |  |  |  |  |  |  |
| Sun ZX, 2008[[150](#_ENREF_150)] | 2006/06 | Zhejiang | East | Cross-sectional | Entertainment venues | 5 | Chinese | 141 | 10 | 7.09% |  |  |  |  |  |  |  |  |  |  |
| Liao M, 2011[[151](#_ENREF_151)] | 2006-2008 | Shandong | East | Cross-sectional | Entertainment venues | 4 | English | 1,211 | 36 | 2.97% |  |  |  |  |  |  |  |  |  |  |
| Kang D, 2011 (a)[[152](#_ENREF_152)] | 2006-2009 | Shandong | East | Cross-sectional | Entertainment venues | 6 | English | 120 | 3 | 2.50% |  |  |  |  |  |  |  |  |  |  |
| Kang D, 2011 (c)[[152](#_ENREF_152)] | 2006-2009 | Shandong | East | Cross-sectional | Entertainment venues | 6 | English | 823 | 22 | 2.70% |  |  |  |  |  |  |  |  |  |  |
| Kang D, 2011 (e)[[152](#_ENREF_152)] | 2006-2009 | Shandong | East | Cross-sectional | Entertainment venues | 4 | English | 244 | 11 | 4.50% |  |  |  |  |  |  |  |  |  |  |
| Hong H, 2010b[[148](#_ENREF_148)] | 2007 | Zhejiang | East | Cross-sectional | Others | 4 | English | 527 | 4 | 0.76% |  |  |  |  |  |  |  |  |  |  |
| Huang Y, 2012[[149](#_ENREF_149)] | 2007 | Anhui | East | Cross-sectional | Entertainment venues | 4 | Chinese | 444 | 3 | 0.68% |  |  |  |  |  |  |  |  |  |  |
| Zeng YB, 2009c[[13](#_ENREF_13)] | 2007 | Shanghai | East | National Sentinel Site | Detaining education center | 4 | Chinese | 448 | 13 | 2.90% |  |  |  |  |  |  |  |  |  |  |
| Chen YL, 2009h[[139](#_ENREF_139)] | 2007 | Fujian | East | Cross-sectional | Entertainment venues | 5 | Chinese | 223 | 26 | 11.66% |  |  |  |  |  |  |  |  |  |  |
| Luo Y, 2008a[[153](#_ENREF_153)] | 2007/02-2007/07 | Zhejiang | East | Cross-sectional | Entertainment venues | 5 | Chinese | 267 | 7 | 2.60% |  |  |  |  |  |  |  |  |  |  |
| Yang JY, 2011[[21](#_ENREF_21)] | 2007/10-2007/11 | Jiangsu | East | Cross-sectional | Entertainment venues | 4 | Chinese | 247 | 13 | 5.26% |  |  |  |  |  |  |  |  |  |  |
| Wang FH, 2009a[[154](#_ENREF_154)] | 2008 | Anhui | East | National Sentinel Site | Entertainment venues | 2 | Chinese | 4,584 | 105 | 2.29% |  |  |  |  |  |  |  |  |  |  |
| Hong H, 2010c[[148](#_ENREF_148)] | 2008 | Zhejiang | East | Cross-sectional | Others | 2 | English | 400 | 0 | 0.00% |  |  |  |  |  |  |  |  |  |  |
| Tang X, 2012[[155](#_ENREF_155)] | 2008 | Shanghai | East | Cross-sectional | Entertainment venues | 4 | Chinese | 244 | 10 | 4.10% |  |  |  |  |  |  |  |  |  |  |
| Tang X, 2012[[155](#_ENREF_155)] | 2008 | Shanghai | East | Cross-sectional | Entertainment venues | 4 | Chinese | 131 | 6 | 4.58% |  |  |  |  |  |  |  |  |  |  |
| Tang X, 2012[[155](#_ENREF_155)] | 2008 | Shanghai | East | Cross-sectional | Entertainment venues | 4 | Chinese | 113 | 4 | 3.54% |  |  |  |  |  |  |  |  |  |  |
| Huang Y, 2012[[149](#_ENREF_149)] | 2008 | Anhui | East | Cross-sectional | Entertainment venues | 4 | Chinese | 402 | 6 | 1.49% |  |  |  |  |  |  |  |  |  |  |
| Liao M, 2012a[[156](#_ENREF_156)] | 2008/02-2008/08 | Shandong | East | Cross-sectional | Entertainment venues | 6 | English | 363 | 10 | 2.80% |  |  |  |  |  |  |  |  |  |  |
| Zhao YQ, 2010a[[23](#_ENREF_23)] | 2008/04-2008/05 | Jiangsu | East | Cross-sectional | Entertainment venues | 4 | Chinese | 396 | 8 | 2.00% |  |  |  |  |  |  |  |  |  |  |
| Xue FH, 2009a[[157](#_ENREF_157)] | 2008/04-2008/06 | Zhejiang | East | Cross-sectional | Entertainment venues | 4 | Chinese | 403 | 6 | 1.49% |  |  |  |  |  |  |  |  |  |  |
| Zhang YH, 2011[[131](#_ENREF_131)] | 2008/04-2008/07 | Zhejiang | East | Cross-sectional | Others | 2 | Chinese | 460 | 20 | 4.30% |  |  |  |  |  |  |  |  |  |  |
| Zhang YH, 2011[[131](#_ENREF_131)] | 2008/04-2008/07 | Shanghai | East | Cross-sectional | Others | 2 | Chinese | 416 | 10 | 2.40% |  |  |  |  |  |  |  |  |  |  |
| Zhang YH, 2011[[131](#_ENREF_131)] | 2008/04-2008/07 | Jiangsu | East | Cross-sectional | Others | 2 | Chinese | 400 | 11 | 2.80% |  |  |  |  |  |  |  |  |  |  |
| Zhang YH, 2011[[131](#_ENREF_131)] | 2008/04-2008/07 | Shandong | East | Cross-sectional | Others | 2 | Chinese | 400 | 5 | 1.30% |  |  |  |  |  |  |  |  |  |  |
| Tang X, 2010[[158](#_ENREF_158)] | 2008/10-2008/11 | Shanghai | East | Cross-sectional | Entertainment venues | 6 | Chinese | 285 | 12 | 4.20% |  |  |  |  |  |  |  |  |  |  |
| Chen JQ, 2009[[104](#_ENREF_104)] | 2008/10-2008/11 | Zhejiang | East | Cross-sectional | Entertainment venues | 3 | Chinese | 111 | 4 | 3.60% |  |  |  |  |  |  |  |  |  |  |
| Wu RH, 2012[[105](#_ENREF_105)] | 2008-2011 | Shanghai | East | Cross-sectional | Detaining education center | 4 | Chinese | 422 | 57 | 13.51% |  |  |  |  |  |  |  |  |  |  |
| Luo XY, 2010a[[159](#_ENREF_159)] | 2009 | Zhejiang | East | Cross-sectional | Entertainment venues | 5 | Chinese | 530 | 13 | 2.45% |  |  |  |  |  |  |  |  |  |  |
| Hong H, 2010d[[148](#_ENREF_148)] | 2009 | Zhejiang | East | Cross-sectional | Others | 2 | English | 407 | 2 | 0.49% |  |  |  |  |  |  |  |  |  |  |
| Luo XY, 2010b[[159](#_ENREF_159)] | 2009 | Zhejiang | East | Cross-sectional | Entertainment venues | 5 | Chinese | 350 | 3 | 0.86% |  |  |  |  |  |  |  |  |  |  |
| Luo XY, 2010c[[159](#_ENREF_159)] | 2009 | Zhejiang | East | Cross-sectional | Entertainment venues | 5 | Chinese | 180 | 10 | 5.56% |  |  |  |  |  |  |  |  |  |  |
| Yang LQ, 2012[[160](#_ENREF_160)] | 2009 | Zhejiang | East | Cross-sectional | Entertainment venues | 3 | Chinese | 403 | 14 | 3.47% |  |  |  |  |  |  |  |  |  |  |
| Chen ZH, 2012[[161](#_ENREF_161)] | 2009 | Zhejiang | East | Cross-sectional | Entertainment venues | 3 | Chinese | 409 | 19 | 4.65% |  |  |  |  |  |  |  |  |  |  |
| Huang Y, 2012[[149](#_ENREF_149)] | 2009 | Anhui | East | Cross-sectional | Entertainment venues | 4 | Chinese | 412 | 3 | 0.73% |  |  |  |  |  |  |  |  |  |  |
| Xi SJ, 2010[[162](#_ENREF_162)] | 2009 | Zhejiang | East | Cross-sectional | Entertainment venues | 5 | Chinese | 210 | 8 | 3.81% |  |  |  |  |  |  |  |  |  |  |
| Li XY, 2011[[25](#_ENREF_25)] | 2009/01-2010/05 | Zhejiang | East | Cross-sectional | Entertainment venues | 3 | Chinese | 98 | 25 | 25.51% |  |  |  |  |  |  |  |  |  |  |
| Wang JH, 2010[[163](#_ENREF_163)] | 2009/04 | Shandong | East | Cross-sectional | Entertainment venues | 5 | Chinese | 348 | 8 | 2.30% |  |  |  |  |  |  |  |  |  |  |
| Shao MC, 2010[[106](#_ENREF_106)] | 2009/04 | Jiangsu | East | Cross-sectional | Entertainment venues | 1 | Chinese | 402 | 15 | 3.70% |  |  |  |  |  |  |  |  |  |  |
| Wang JH, 2010b[[163](#_ENREF_163)] | 2009/04 | Shandong | East | Cross-sectional | Entertainment venues | 5 | Chinese | 298 | 3 | 1.01% |  |  |  |  |  |  |  |  |  |  |
| Wang JH, 2010c[[163](#_ENREF_163)] | 2009/04 | Shandong | East | Cross-sectional | Entertainment venues | 5 | Chinese | 50 | 5 | 10.00% |  |  |  |  |  |  |  |  |  |  |
| Wang B, 2010a[[164](#_ENREF_164)] | 2009/04-2009/06 | Jiangsu | East | Cross-sectional | Entertainment venues | 1 | Chinese | 400 | 16 | 4.00% |  |  |  |  |  |  |  |  |  |  |
| Wang B, 2010b[[164](#_ENREF_164)] | 2009/04-2009/06 | Jiangsu | East | Cross-sectional | Entertainment venues | 1 | Chinese | 133 | 5 | 3.76% |  |  |  |  |  |  |  |  |  |  |
| Wang B, 2010c[[164](#_ENREF_164)] | 2009/04-2009/06 | Jiangsu | East | Cross-sectional | Entertainment venues | 1 | Chinese | 179 | 4 | 2.23% |  |  |  |  |  |  |  |  |  |  |
| Wang B, 2010d[[164](#_ENREF_164)] | 2009/04-2009/06 | Jiangsu | East | Cross-sectional | Entertainment venues | 1 | Chinese | 34 | 5 | 14.71% |  |  |  |  |  |  |  |  |  |  |
| Wang B, 2010e[[164](#_ENREF_164)] | 2009/04-2009/06 | Jiangsu | East | Cross-sectional | Entertainment venues | 1 | Chinese | 54 | 2 | 3.70% |  |  |  |  |  |  |  |  |  |  |
| Chen GS, 2010[[165](#_ENREF_165)] | 2009/04-2009/06 | Jiangxi | East | Cross-sectional | Entertainment venues | 6 | Chinese | 401 | 8 | 2.00% |  |  |  |  |  |  |  |  |  |  |
| Jin HJ, 2010a[[166](#_ENREF_166)] | 2009/04-2009/07 | Zhejiang | East | National Sentinel Site | Entertainment venues | 4 | Chinese | 400 | 12 | 3.00% |  |  |  |  |  |  |  |  |  |  |
| Liao M, 2012b[[156](#_ENREF_156)] | 2009/05-2009/10 | Shandong | East | Cross-sectional | Entertainment venues | 6 | English | 432 | 10 | 2.20% |  |  |  |  |  |  |  |  |  |  |
| Chen XS, 2012 (b)[[167](#_ENREF_167)] | 2009/06-2009/09 | Jiangsu | East | Cross-sectional | Entertainment venues | 4 | English | 1,791 | 65 | 3.60% |  |  |  |  |  |  |  |  |  |  |
| Ma JH, 2012[[168](#_ENREF_168)] | 2009/07 | Jiangsu | East | Cross-sectional | Others | 4 | Chinese | 150 | 17 | 11.30% |  |  |  |  |  |  |  |  |  |  |
| Wei ZY, 2010a[[27](#_ENREF_27)] | 2009/07-2009/09 | Jiangsu | East | Cross-sectional | Entertainment venues | 2 | Chinese | 940 | 79 | 8.40% |  |  |  |  |  |  |  |  |  |  |
| Chen SX, 2011[[169](#_ENREF_169)] | 2009/07-2009/09 | Shandong | East | Cross-sectional | Entertainment venues | 4 | Chinese | 236 | 13 | 5.51% |  |  |  |  |  |  |  |  |  |  |
| Zhang QQ, 2012[[26](#_ENREF_26)] | 2009/07-2009/09 | Jiangsu | East | Cross-sectional | Entertainment venues | 4 | Chinese | 1,806 | 152 | 8.40% |  |  |  |  |  |  |  |  |  |  |
| Zhang QQ, 2012[[26](#_ENREF_26)] | 2009/10-2009/12 | Jiangsu | East | Cross-sectional | Entertainment venues | 4 | Chinese | 737 | 34 | 4.60% |  |  |  |  |  |  |  |  |  |  |
| Ma JH, 2012[[168](#_ENREF_168)] | 2009/11 | Jiangsu | East | Cross-sectional | Others | 2 | Chinese | 150 | 13 | 8.67% |  |  |  |  |  |  |  |  |  |  |
| Chen CG, 2011[[170](#_ENREF_170)] | 2009/11-2010/01 | Fujian | East | Cross-sectional | Entertainment venues | 2 | Chinese | 225 | 0 | 0.00% |  |  |  |  |  |  |  |  |  |  |
| Chen MF, 2011[[171](#_ENREF_171)] | 2009-2010 | Zhejiang | East | Cross-sectional | Entertainment venues | 3 | Chinese | 739 | 7 | 0.95% |  |  |  |  |  |  |  |  |  |  |
| Qian ZH, 2012[[172](#_ENREF_172)] | 2009-2010 | Jiangsu | East | Cross-sectional | Detaining education center | 4 | Chinese | 578 | 29 | 5.02% |  |  |  |  |  |  |  |  |  |  |
| Gan WH, 2012[[173](#_ENREF_173)] | 2009-2011 | Shanghai | East | Cross-sectional | National sentinel site | 3 | Chinese | 1,240 | 25 | 2.02% |  |  |  |  |  |  |  |  |  |  |
| Chen ZH, 2012[[161](#_ENREF_161)] | 2009-2011 | Zhejiang | East | Cross-sectional | Entertainment venues | 3 | Chinese | 1,163 | 70 | 6.02% |  |  |  |  |  |  |  |  |  |  |
| Ma JH, 2012[[168](#_ENREF_168)] | 2009-2011 | Jiangsu | East | Cross-sectional | Others | 2 | Chinese | 1,203 | 167 | 13.88% |  |  |  |  |  |  |  |  |  |  |
| Miao XL, 2011[[174](#_ENREF_174)] | 2010 | Jiangsu | East | Cross-sectional | Entertainment venues | 4 | Chinese | 800 | 37 | 4.60% |  |  |  |  |  |  |  |  |  |  |
| Ma P, 2011[[175](#_ENREF_175)] | 2010 | Jiangsu | East | Cross-sectional | Detaining education center | 3 | Chinese | 798 | 48 | 6.02% |  |  |  |  |  |  |  |  |  |  |
| Zhang HF, 2012[[176](#_ENREF_176)] | 2010 | Zhejiang | East | Cross-sectional | Entertainment venues | 4 | Chinese | 229 | 8 | 3.49% |  |  |  |  |  |  |  |  |  |  |
| Zhang HF, 2012[[176](#_ENREF_176)] | 2010 | Zhejiang | East | Cross-sectional | Entertainment venues | 4 | Chinese | 432 | 45 | 10.42% |  |  |  |  |  |  |  |  |  |  |
| Yang LQ, 2012[[160](#_ENREF_160)] | 2010 | Zhejiang | East | Cross-sectional | Entertainment venues | 3 | Chinese | 426 | 27 | 6.34% |  |  |  |  |  |  |  |  |  |  |
| Tang Y, 2012[[177](#_ENREF_177)] | 2010 | Shanghai | East | Cross-sectional | Others | 2 | Chinese | 2,011 | 135 | 6.71% |  |  |  |  |  |  |  |  |  |  |
| Chen ZH, 2012[[161](#_ENREF_161)] | 2010 | Zhejiang | East | Cross-sectional | Entertainment venues | 3 | Chinese | 400 | 22 | 5.50% |  |  |  |  |  |  |  |  |  |  |
| Ma P, 2012[[178](#_ENREF_178)] | 2010 | Jiangsu | East | Cross-sectional | Entertainment venues | 3 | Chinese | 798 | 48 | 6.02% |  |  |  |  |  |  |  |  |  |  |
| Tang ZL, 2011[[28](#_ENREF_28)] | 2010/03-2010/07 | Shandong | East | Cross-sectional | Entertainment venues | 4 | Chinese | 106 | 12 | 11.30% |  |  |  |  |  |  |  |  |  |  |
| Tang ZL, 2011[[28](#_ENREF_28)] | 2010/03-2010/07 | Shandong | East | Cross-sectional | Entertainment venues | 4 | Chinese | 216 | 10 | 4.60% |  |  |  |  |  |  |  |  |  |  |
| Liu LL, 2011[[179](#_ENREF_179)] | 2010/04-2010/06 | Jiangsu | East | Cross-sectional | Entertainment venues | 6 | Chinese | 407 | 16 | 3.90% |  |  |  |  |  |  |  |  |  |  |
| Ma JH, 2012[[168](#_ENREF_168)] | 2010/04-2010/06 | Jiangsu | East | Cross-sectional | Others | 2 | Chinese | 400 | 78 | 19.50% |  |  |  |  |  |  |  |  |  |  |
| Jiang J, 2012[[180](#_ENREF_180)] | 2010/07 | Zhejiang | East | Cross-sectional | Detaining education center | 4 | Chinese | 439 | 96 | 21.87% |  |  |  |  |  |  |  |  |  |  |
| Ye YD, 2011[[181](#_ENREF_181)] | 2010/11-2010/12 | Jiangsu | East | Cross-sectional | Entertainment venues | 4 | Chinese | 418 | 34 | 8.13% |  |  |  |  |  |  |  |  |  |  |
| Ye YD, 2011[[182](#_ENREF_182)] | 2010/11-2010/12 | Jiangsu | East | Cross-sectional | Entertainment venues | 3 | Chinese | 1,619 | 96 | 5.93% |  |  |  |  |  |  |  |  |  |  |
| Ma JH, 2012[[168](#_ENREF_168)] | 2010/12 | Jiangsu | East | Cross-sectional | Others | 2 | Chinese | 103 | 5 | 4.85% |  |  |  |  |  |  |  |  |  |  |
| Qin CZ, 2012[[183](#_ENREF_183)] | 2010/6-2011/04 | Jiangsu | East | Cross-sectional | Entertainment venues | 4 | Chinese | 817 | 24 | 2.90% |  |  |  |  |  |  |  |  |  |  |
| Qin CZ, 2012[[183](#_ENREF_183)] | 2010/6-2011/04 | Jiangsu | East | Cross-sectional | Entertainment venues | 4 | Chinese | 215 | 5 | 2.30% |  |  |  |  |  |  |  |  |  |  |
| Qin CZ, 2012[[183](#_ENREF_183)] | 2010/6-2011/04 | Jiangsu | East | Cross-sectional | Entertainment venues | 4 | Chinese | 444 | 12 | 2.70% |  |  |  |  |  |  |  |  |  |  |
| Qin CZ, 2012[[183](#_ENREF_183)] | 2010/6-2011/04 | Jiangsu | East | Cross-sectional | Entertainment venues | 4 | Chinese | 158 | 7 | 4.40% |  |  |  |  |  |  |  |  |  |  |
| Zhang HF, 2012[[176](#_ENREF_176)] | 2010-2011 | Zhejiang | East | Cross-sectional | Entertainment venues | 4 | Chinese | 1,295 | 100 | 7.20% |  |  |  |  |  |  |  |  |  |  |
| Wu J, 2012[[184](#_ENREF_184)] | 2011 | Jiangsu | East | Cross-sectional | Entertainment venues | 4 | Chinese | 405 | 39 | 9.63% |  |  |  |  |  |  |  |  |  |  |
| Wu J, 2012[[184](#_ENREF_184)] | 2011 | Jiangsu | East | Cross-sectional | Entertainment venues | 4 | Chinese | 308 | 20 | 6.49% |  |  |  |  |  |  |  |  |  |  |
| Wu J, 2012[[184](#_ENREF_184)] | 2011 | Jiangsu | East | Cross-sectional | Entertainment venues | 4 | Chinese | 97 | 19 | 19.59% |  |  |  |  |  |  |  |  |  |  |
| Sun BJ, 2012[[185](#_ENREF_185)] | 2011 | Shandong | East | Cross-sectional | Entertainment venues | 4 | Chinese | 372 | 13 | 3.49% |  |  |  |  |  |  |  |  |  |  |
| Zhang HF, 2012[[176](#_ENREF_176)] | 2011 | Zhejiang | East | Cross-sectional | Entertainment venues | 4 | Chinese | 157 | 1 | 0.64% |  |  |  |  |  |  |  |  |  |  |
| Zhang HF, 2012[[176](#_ENREF_176)] | 2011 | Zhejiang | East | Cross-sectional | Entertainment venues | 4 | Chinese | 477 | 46 | 9.64% |  |  |  |  |  |  |  |  |  |  |
| Yang LQ, 2012[[160](#_ENREF_160)] | 2011 | Zhejiang | East | Cross-sectional | Entertainment venues | 3 | Chinese | 440 | 20 | 4.55% |  |  |  |  |  |  |  |  |  |  |
| Yang YH, 2012[[186](#_ENREF_186)] | 2011 | Fujian | East | Cross-sectional | National sentinel site | 4 | Chinese | 868 | 8 | 1.00% |  |  |  |  |  |  |  |  |  |  |
| Tang Y, 2012[[177](#_ENREF_177)] | 2011 | Shanghai | East | Cross-sectional | Others | 2 | Chinese | 2,972 | 89 | 2.99% |  |  |  |  |  |  |  |  |  |  |
| Chen ZH, 2012[[161](#_ENREF_161)] | 2011 | Zhejiang | East | Cross-sectional | Entertainment venues | 3 | Chinese | 354 | 29 | 8.19% |  |  |  |  |  |  |  |  |  |  |
| Chen YH, 2012[[187](#_ENREF_187)] | 2011/04-2011/06 | Jiangsu | East | Cross-sectional | Entertainment venues | 4 | Chinese | 400 | 24 | 6.00% |  |  |  |  |  |  |  |  |  |  |
| Ma JH, 2012[[168](#_ENREF_168)] | 2011/04-2011/06 | Jiangsu | East | Cross-sectional | Others | 2 | Chinese | 400 | 54 | 13.50% |  |  |  |  |  |  |  |  |  |  |
| Xu JS, 2012[[188](#_ENREF_188)] | 2011/04-2011/07 | Jiangsu | East | Cross-sectional | Others | 4 | Chinese | 10,916 | 581 | 5.32% |  |  |  |  |  |  |  |  |  |  |
| Xu JS, 2012[[188](#_ENREF_188)] | 2011/04-2011/07 | Jiangsu | East | Cross-sectional | Others | 4 | Chinese | 1,591 | 34 | 2.14% |  |  |  |  |  |  |  |  |  |  |
| Xu JS, 2012[[188](#_ENREF_188)] | 2011/04-2011/07 | Jiangsu | East | Cross-sectional | Others | 4 | Chinese | 5,502 | 198 | 3.60% |  |  |  |  |  |  |  |  |  |  |
| Xu JS, 2012[[188](#_ENREF_188)] | 2011/04-2011/07 | Jiangsu | East | Cross-sectional | Others | 4 | Chinese | 3,748 | 344 | 8.91% |  |  |  |  |  |  |  |  |  |  |
| Pan GL, 2012[[189](#_ENREF_189)] | 2011/04-2011/07 | Zhejiang | East | Cross-sectional | Entertainment venues | 4 | Chinese | 617 | 82 | 13.29% |  |  |  |  |  |  |  |  |  |  |
| Pan GL, 2012[[189](#_ENREF_189)] | 2011/04-2011/07 | Zhejiang | East | Cross-sectional | Entertainment venues | 4 | Chinese | 88 | 5 | 5.68% |  |  |  |  |  |  |  |  |  |  |
| Pan GL, 2012[[189](#_ENREF_189)] | 2011/04-2011/07 | Zhejiang | East | Cross-sectional | Entertainment venues | 4 | Chinese | 255 | 34 | 13.33% |  |  |  |  |  |  |  |  |  |  |
| Pan GL, 2012[[189](#_ENREF_189)] | 2011/04-2011/07 | Zhejiang | East | Cross-sectional | Entertainment venues | 4 | Chinese | 274 | 43 | 15.69% |  |  |  |  |  |  |  |  |  |  |
| Tao SF, 2012[[190](#_ENREF_190)] | 2011/06-2011/12 | Anhui | East | Cross-sectional | Entertainment venues | 2 | Chinese | 40 | 5 | 12.50% |  |  |  |  |  |  |  |  |  |  |
| Wu J, 2010a[[191](#_ENREF_191)] | 2005/11 | N/A | N/A | Cross-sectional | Entertainment venues | 3 | Chinese | 257 | 2 | 0.78% |  |  |  |  |  |  |  |  |  |  |
| Li DL, 2006a[[29](#_ENREF_29)] | 2001 | Beijing | North | Cross-sectional | National sentinel site | 4 | Chinese | 354 | 17 | 5.00% |  |  |  |  |  |  |  |  |  |  |
| Li DL, 2006b[[29](#_ENREF_29)] | 2002 | Beijing | North | Cross-sectional | National sentinel site | 4 | Chinese | 571 | 24 | 4.70% |  |  |  |  |  |  |  |  |  |  |
| Li DL, 2006c[[29](#_ENREF_29)] | 2003 | Beijing | North | Cross-sectional | National sentinel site | 4 | Chinese | 174 | 3 | 2.19% |  |  |  |  |  |  |  |  |  |  |
| Li DL, 2006d[[29](#_ENREF_29)] | 2004 | Beijing | North | Cross-sectional | National sentinel site | 4 | Chinese | 413 | 29 | 7.02% |  |  |  |  |  |  |  |  |  |  |
| Liu YQ, 2007[[192](#_ENREF_192)] | 2005 | Beijing | North | Cross-sectional | Entertainment venues | 7 | Chinese | 203 | 9 | 4.43% |  |  |  |  |  |  |  |  |  |  |
| Liu YJ, 2006[[193](#_ENREF_193)] | 2005/01-2005/12 | Beijing | North | Cross-sectional | Entertainment venues | 5 | Chinese | 403 | 47 | 11.66% |  |  |  |  |  |  |  |  |  |  |
| Bai JM, 2006[[194](#_ENREF_194)] | 2005/03-2005/05 | Beijing | North | Cross-sectional | Entertainment venues | 4 | Chinese | 109 | 4 | 3.67% |  |  |  |  |  |  |  |  |  |  |
| Xia JH, 2010a[[195](#_ENREF_195)] | 2006 | Tianjin | North | National sentinel site | Entertainment venues | 5 | Chinese | 449 | 25 | 5.57% |  |  |  |  |  |  |  |  |  |  |
| Gao JM, 2008[[196](#_ENREF_196)] | 2006 | Beijing | North | National sentinel site | Entertainment venues | 6 | Chinese | 132 | 7 | 5.30% |  |  |  |  |  |  |  |  |  |  |
| Liu LR, 2007a[[197](#_ENREF_197)] | 2006/05-2006/10 | Beijing | North | Cross-sectional | Entertainment venues | 7 | Chinese | 341 | 2 | 0.59% |  |  |  |  |  |  |  |  |  |  |
| Ao X, 2008[[198](#_ENREF_198)] | 2006/08-2006/10 | Beijing | North | Cross-sectional | Entertainment venues | 7 | Chinese | 105 | 3 | 2.86% |  |  |  |  |  |  |  |  |  |  |
| Dong XY, 2009a[[199](#_ENREF_199)] | 2007/08-2007/10 | Tianjin | North | National Sentinel Site | Entertainment venues | 5 | Chinese | 178 | 20 | 11.24% |  |  |  |  |  |  |  |  |  |  |
| Xia JH, 2010c[[195](#_ENREF_195)] | 2008 | Tianjin | North | National sentinel site | Detaining education center | 5 | Chinese | 148 | 10 | 6.76% |  |  |  |  |  |  |  |  |  |  |
| Zhang YH, 2011[[131](#_ENREF_131)] | 2008/04-2008/07 | Beijing | North | Cross-sectional | Others | 2 | Chinese | 2,688 | 38 | 1.40% |  |  |  |  |  |  |  |  |  |  |
| Zhang YH, 2011[[131](#_ENREF_131)] | 2008/04-2008/07 | Tianjin | North | Cross-sectional | Others | 2 | Chinese | 534 | 31 | 5.90% |  |  |  |  |  |  |  |  |  |  |
| Jiang DK, 2010a[[200](#_ENREF_200)] | 2009 | Shanxi | North | National sentinel sites | Detaining education center | 6 | Chinese | 380 | 1 | 0.26% |  |  |  |  |  |  |  |  |  |  |
| Xu YJ, 2011[[201](#_ENREF_201)] | 2009 | Shanxi | North | Cross-sectional | Entertainment venues | 3 | Chinese | 5,513 | 40 | 0.73% |  |  |  |  |  |  |  |  |  |  |
| Xu YJ, 2011[[201](#_ENREF_201)] | 2009 | Shanxi | North | Cross-sectional | Entertainment venues | 3 | Chinese | 4,885 | 36 | 0.74% |  |  |  |  |  |  |  |  |  |  |
| Xu YJ, 2011[[201](#_ENREF_201)] | 2009 | Shanxi | North | Cross-sectional | Entertainment venues | 3 | Chinese | 628 | 4 | 0.64% |  |  |  |  |  |  |  |  |  |  |
| Li M, 2012[[202](#_ENREF_202)] | 2009 | Beijing | North | Cross-sectional | Entertainment venues | 4 | Chinese | 200 | 3 | 1.50% |  |  |  |  |  |  |  |  |  |  |
| Li M, 2012[[202](#_ENREF_202)] | 2010 | Beijing | North | Cross-sectional | Entertainment venues | 4 | Chinese | 200 | 2 | 1.00% |  |  |  |  |  |  |  |  |  |  |
| Feng N, 2011[[203](#_ENREF_203)] | 2010/04-2010/06 | Shanxi | North | Cross-sectional | National sentinel site | 4 | Chinese | 400 | 0 | 0.00% |  |  |  |  |  |  |  |  |  |  |
| Li BY, 2012[[204](#_ENREF_204)] | 2010/04-2010/07 | Tianjin | North | Cross-sectional | Others | 4 | Chinese | 407 | 13 | 3.19% |  |  |  |  |  |  |  |  |  |  |
| Li M, 2012[[202](#_ENREF_202)] | 2011 | Beijing | North | Cross-sectional | Entertainment venues | 4 | Chinese | 200 | 2 | 1.00% |  |  |  |  |  |  |  |  |  |  |
| Li F, 2012[[205](#_ENREF_205)] | 2011 | Beijing | North | Cross-sectional | Detaining education center | 4 | Chinese | 290 | 5 | 1.70% |  |  |  |  |  |  |  |  |  |  |
| Liu ZJ, 2012[[206](#_ENREF_206)] | 2011/04-2011/06 | Hebei | North | Cross-sectional | Entertainment venues | 4 | Chinese | 400 | 2 | 0.50% |  |  |  |  |  |  |  |  |  |  |
| Zhang P, 2012[[207](#_ENREF_207)] |  | Jilin | Northeast | Cross-sectional | Others | 4 | Chinese | 400 | 13 | 3.30% |  |  |  |  |  |  |  |  |  |  |
| Cui YZ, 2011[[208](#_ENREF_208)] | 2006 | Heilongjiang | Northeast | Cross-sectional | Entertainment venues | 4 | Chinese | 417 | 5 | 1.20% |  |  |  |  |  |  |  |  |  |  |
| Qi GH, 2008[[209](#_ENREF_209)] | 2006/07 | Jilin | Northeast | Cross-sectional | Entertainment venues | 4 | Chinese | 485 | 86 | 17.70% |  |  |  |  |  |  |  |  |  |  |
| Qi GH, 2008b[[209](#_ENREF_209)] | 2006/07 | Jilin | Northeast | Cross-sectional | Entertainment venues | 4 | Chinese | 124 | 18 | 14.52% |  |  |  |  |  |  |  |  |  |  |
| Qi GH, 2008c[[209](#_ENREF_209)] | 2006/07 | Jilin | Northeast | Cross-sectional | Entertainment venues | 4 | Chinese | 239 | 44 | 18.41% |  |  |  |  |  |  |  |  |  |  |
| Qi GH, 2008e[[209](#_ENREF_209)] | 2006/07 | Jilin | Northeast | Cross-sectional | Entertainment venues | 4 | Chinese | 122 | 24 | 19.67% |  |  |  |  |  |  |  |  |  |  |
| Cui YZ, 2011[[208](#_ENREF_208)] | 2007 | Heilongjiang | Northeast | Cross-sectional | Entertainment venues | 4 | Chinese | 447 | 15 | 3.40% |  |  |  |  |  |  |  |  |  |  |
| Cui YZ, 2011[[208](#_ENREF_208)] | 2008 | Heilongjiang | Northeast | Cross-sectional | Entertainment venues | 4 | Chinese | 410 | 10 | 2.40% |  |  |  |  |  |  |  |  |  |  |
| Zhang YH, 2011[[131](#_ENREF_131)] | 2008/04-2008/07 | Liaoning | Northeast | Cross-sectional | Others | 2 | Chinese | 601 | 5 | 0.80% |  |  |  |  |  |  |  |  |  |  |
| Zhang YH, 2011[[131](#_ENREF_131)] | 2008/04-2008/07 | Heilongjiang | Northeast | Cross-sectional | Others | 2 | Chinese | 447 | 15 | 3.40% |  |  |  |  |  |  |  |  |  |  |
| Cui YZ, 2011[[208](#_ENREF_208)] | 2009 | Heilongjiang | Northeast | Cross-sectional | Entertainment venues | 4 | Chinese | 447 | 11 | 2.50% |  |  |  |  |  |  |  |  |  |  |
| Zheng J, 2012[[210](#_ENREF_210)] | 2009 | Liaoning | Northeast | Cross-sectional | Entertainment venues | 4 | Chinese | 400 | 0 | 0.00% |  |  |  |  |  |  |  |  |  |  |
| Zhou D, 2012[[211](#_ENREF_211)] | 2011 | Liaoning | Northeast | Cross-sectional | Entertainment venues | 4 | Chinese | 6,838 | 83 | 1.20% |  |  |  |  |  |  |  |  |  |  |
| Yu QL, 2007[[212](#_ENREF_212)] |  | Gansu | Northwest | Cross-sectional | Others | 4 | Chinese | 297 | 8 | 2.70% |  |  |  |  |  |  |  |  |  |  |
| Zhang M, 2006c[[213](#_ENREF_213)] | 2000 | Xinjiang | Northwest | National Sentinel Site | Detaining education center | 3 | Chinese | 124 | 9 | 7.26% |  |  |  |  |  |  |  |  |  |  |
| Zhang M, 2006d[[213](#_ENREF_213)] | 2001 | Xinjiang | Northwest | National Sentinel Site | Detaining education center | 3 | Chinese | 335 | 25 | 7.46% |  |  |  |  |  |  |  |  |  |  |
| Wei XW, 2004a[[214](#_ENREF_214)] | 2001/06-2001/08 | Xinjiang | Northwest | Cross-sectional | Detaining education center | 5 | Chinese | 181 | 6 | 3.31% |  |  |  |  |  |  |  |  |  |  |
| Zhang M, 2006e[[213](#_ENREF_213)] | 2002 | Xinjiang | Northwest | National Sentinel Site | Detaining education center | 3 | Chinese | 184 | 8 | 4.35% |  |  |  |  |  |  |  |  |  |  |
| Xi SH, 2004[[33](#_ENREF_33)] | 2002/05-2002/07 | Gansu | Northwest | Cross-sectional | Entertainment venues | 7 | Chinese | 88 | 12 | 13.60% |  |  |  |  |  |  |  |  |  |  |
| Zhang M, 2006f[[213](#_ENREF_213)] | 2003 | Xinjiang | Northwest | National Sentinel Site | Detaining education center | 3 | Chinese | 134 | 11 | 8.21% |  |  |  |  |  |  |  |  |  |  |
| Ji CH, 2009a[[215](#_ENREF_215)] | 2004 | Shaanxi | Northwest | National Sentinel Site | Entertainment venues | 4 | Chinese | 296 | 2 | 0.68% |  |  |  |  |  |  |  |  |  |  |
| Lin L, 2009b[[216](#_ENREF_216)] | 2004 | Xinjiang | Northwest | Cross-sectional | National sentinel site | 4 | Chinese | 332 | 8 | 2.41% |  |  |  |  |  |  |  |  |  |  |
| Zhang M, 2006g[[213](#_ENREF_213)] | 2004 | Xinjiang | Northwest | National Sentinel Site | Detaining education center | 3 | Chinese | 81 | 3 | 3.70% |  |  |  |  |  |  |  |  |  |  |
| Song YR, 2005a[[217](#_ENREF_217)] | 2004/08 | Xinjiang | Northwest | Cross-sectional | Entertainment venues | 4 | Chinese | 258 | 22 | 8.53% |  |  |  |  |  |  |  |  |  |  |
| Ji CH, 2009b[[215](#_ENREF_215)] | 2005 | Shaanxi | Northwest | National Sentinel Site | Entertainment venues | 4 | Chinese | 305 | 0 | 0.00% |  |  |  |  |  |  |  |  |  |  |
| Lin L, 2009c[[216](#_ENREF_216)] | 2005 | Xinjiang | Northwest | Cross-sectional | National sentinel site | 4 | Chinese | 201 | 2 | 1.00% |  |  |  |  |  |  |  |  |  |  |
| Zhang L, 2007a[[218](#_ENREF_218)] | 2005/11-2006/05 | Gansu | Northwest | National Sentinel Site | Entertainment venues | 1 | Chinese | 376 | 0 | 0.00% |  |  |  |  |  |  |  |  |  |  |
| Ji CH, 2009c[[215](#_ENREF_215)] | 2006 | Shaanxi | Northwest | National Sentinel Site | Entertainment venues | 4 | Chinese | 303 | 0 | 0.00% |  |  |  |  |  |  |  |  |  |  |
| Lin L, 2009e[[216](#_ENREF_216)] | 2006 | Xinjiang | Northwest | Cross-sectional | National sentinel site | 4 | Chinese | 755 | 4 | 0.53% |  |  |  |  |  |  |  |  |  |  |
| Ma JX, 2012[[219](#_ENREF_219)] | 2006 | Gansu | Northwest | Cross-sectional | Entertainment venues | 4 | Chinese | 403 | 5 | 1.24% |  |  |  |  |  |  |  |  |  |  |
| Ma JX, 2012[[219](#_ENREF_219)] | 2006-2010 | Gansu | Northwest | Cross-sectional | Entertainment venues | 4 | Chinese | 2,023 | 38 | 1.88% |  |  |  |  |  |  |  |  |  |  |
| Lin L, 2009g[[216](#_ENREF_216)] | 2007 | Xinjiang | Northwest | Cross-sectional | Entertainment venues | 4 | Chinese | 268 | 8 | 2.99% |  |  |  |  |  |  |  |  |  |  |
| Ma JX, 2012[[219](#_ENREF_219)] | 2007 | Gansu | Northwest | Cross-sectional | Entertainment venues | 4 | Chinese | 418 | 10 | 2.39% |  |  |  |  |  |  |  |  |  |  |
| Lin B, 2009a[[220](#_ENREF_220)] | 2007/01 | Xinjiang | Northwest | Cross-sectional | Entertainment venues | 1 | Chinese | 268 | 8 | 2.99% |  |  |  |  |  |  |  |  |  |  |
| Yang SP, 2008a[[38](#_ENREF_38)] | 2007/08-2007/09 | Xinjiang | Northwest | Cross-sectional | Entertainment venues | 3 | Chinese | 251 | 48 | 19.12% |  |  |  |  |  |  |  |  |  |  |
| Lin L, 2009i[[216](#_ENREF_216)] | 2008 | Xinjiang | Northwest | Cross-sectional | National sentinel site | 4 | Chinese | 300 | 2 | 0.67% |  |  |  |  |  |  |  |  |  |  |
| Ma JX, 2012[[219](#_ENREF_219)] | 2008 | Gansu | Northwest | Cross-sectional | Entertainment venues | 4 | Chinese | 402 | 15 | 3.73% |  |  |  |  |  |  |  |  |  |  |
| Zhang YH, 2011[[131](#_ENREF_131)] | 2008/04-2008/07 | Shaanxi | Northwest | Cross-sectional | Others | 2 | Chinese | 400 | 6 | 1.50% |  |  |  |  |  |  |  |  |  |  |
| Liang SP, 2010a[[221](#_ENREF_221)] | 2008/05 | Ningxia | Northwest | Cross-sectional | Others | 4 | Chinese | 307 | 24 | 7.82% |  |  |  |  |  |  |  |  |  |  |
| Gao W, 2012 (a)[[40](#_ENREF_40)] | 2008/11-2009/01 | Gansu | Northwest | Cross-sectional | Entertainment venues | 7 | English | 350 | 20 | 5.71% |  |  |  |  |  |  |  |  |  |  |
| Zhao AL, 2011[[222](#_ENREF_222)] | 2009 | Gansu | Northwest | Cross-sectional | Entertainment venues | 0 | Chinese | 350 | 35 | 10.00% |  |  |  |  |  |  |  |  |  |  |
| Ma L, 2011[[223](#_ENREF_223)] | 2009 | Xinjiang | Northwest | Cross-sectional | Entertainment venues | 4 | Chinese | 150 | 7 | 4.67% |  |  |  |  |  |  |  |  |  |  |
| Ma JX, 2012[[219](#_ENREF_219)] | 2009 | Gansu | Northwest | Cross-sectional | Entertainment venues | 4 | Chinese | 400 | 6 | 1.50% |  |  |  |  |  |  |  |  |  |  |
| Xu YJ, 2011a[[224](#_ENREF_224)] | 2009 | Shaanxi | Northwest | Cross-sectional | Others | 4 | Chinese | 5,513 | 40 | 0.07% |  |  |  |  |  |  |  |  |  |  |
| Hu XQ, 2011[[225](#_ENREF_225)] | 2010 | Gansu | Northwest | Cross-sectional | Others | 4 | Chinese | 400 | 11 | 2.75% |  |  |  |  |  |  |  |  |  |  |
| Wu R, 2012[[226](#_ENREF_226)] | 2010 | Xinjiang | Northwest | Cross-sectional | Entertainment venues | 4 | Chinese | 412 | 0 | 0.00% |  |  |  |  |  |  |  |  |  |  |
| Ma JX, 2012[[219](#_ENREF_219)] | 2010 | Gansu | Northwest | Cross-sectional | Entertainment venues | 4 | Chinese | 400 | 2 | 0.50% |  |  |  |  |  |  |  |  |  |  |
| Tao LD, 2011[[227](#_ENREF_227)] | 2010/04-2010/10 | Gansu | Northwest | Cross-sectional | Entertainment venues | 4 | Chinese | 1,200 | 108 | 9.00% |  |  |  |  |  |  |  |  |  |  |
| Guo H, 2012[[228](#_ENREF_228)] | 2010-2011 | Gansu | Northwest | Cross-sectional | Entertainment venues | 3 | Chinese | 820 | 2 | 0.20% |  |  |  |  |  |  |  |  |  |  |
| Xu G, 2012[[229](#_ENREF_229)] | 2000-2005 | Guangdong | South Central | Cross-sectional | Detaining education center | 4 | Chinese | 9,566 | 804 | 8.40% |  |  |  |  |  |  |  |  |  |  |
| Wang LR, 2004[[230](#_ENREF_230)] | 2000/01-2003/09 | Guangdong | South Central | Cross-sectional | Detaining education center | 3 | Chinese | 387 | 133 | 34.37% |  |  |  |  |  |  |  |  |  |  |
| Zhong N, 2002i[[231](#_ENREF_231)] | 2000 | Hainan | South Central | National Sentinel Site | Entertainment venues | 3 | Chinese | 330 | 46 | 13.94% |  |  |  |  |  |  |  |  |  |  |
| Zhang CJ, 2001[[41](#_ENREF_41)] | 2000/03-2000/08 | Henan | South Central | Cross-sectional | Detaining education center | 3 | Chinese | 65 | 2 | 3.08% |  |  |  |  |  |  |  |  |  |  |
| Pei DN, 2002[[232](#_ENREF_232)] | 2000/09 - 2001/08 | Hainan | South Central | Cross-sectional | Detaining education center | 5 | Chinese | 317 | 56 | 17.67% |  |  |  |  |  |  |  |  |  |  |
| Sun J, 2003[[113](#_ENREF_113)] | 2001/09-2001/12 | Henan | South Central | Cross-sectional | Entertainment venues | 2 | Chinese | 220 | 2 | 0.91% |  |  |  |  |  |  |  |  |  |  |
| Zhnagdan C, 2008[[42](#_ENREF_42)] | 2001/10 | Hubei | South Central | Cross-sectional | Entertainment venues | 3 | English | 170 | 11 | 6.47% |  |  |  |  |  |  |  |  |  |  |
| Liang QX, 2004[[233](#_ENREF_233)] | 2002/01-2002/12 | Guangxi | South Central | Cross-sectional | Detaining education center | 3 | Chinese | 393 | 69 | 17.56% |  |  |  |  |  |  |  |  |  |  |
| Zheng K, 2003[[234](#_ENREF_234)] | 2002/06-2008/12 | Hainan | South Central | National Sentinel Site | Detaining education center | 3 | Chinese | 1,022 | 299 | 29.26% |  |  |  |  |  |  |  |  |  |  |
| Liang BH, 2008[[115](#_ENREF_115)] | 2002-2005 | Guangdong | South Central | Cross-sectional | Detaining education center | 5 | Chinese | 1,508 | 145 | 9.62% |  |  |  |  |  |  |  |  |  |  |
| Wang XX, 2005[[235](#_ENREF_235)] | 2003/08 | Guangdong | South Central | Cross-sectional | Detaining education center | 4 | Chinese | 103 | 12 | 11.65% |  |  |  |  |  |  |  |  |  |  |
| Tang GB, 2007a[[48](#_ENREF_48)] | 2004 | Hunan | South Central | Cross-sectional | Entertainment venues | 4 | Chinese | 397 | 9 | 2.30% |  |  |  |  |  |  |  |  |  |  |
| Wu YQ, 2005[[236](#_ENREF_236)] | 2004 | Hainan | South Central | Cross-sectional | Detaining education center | 3 | Chinese | 281 | 15 | 5.30% |  |  |  |  |  |  |  |  |  |  |
| Wang H, 2005[[51](#_ENREF_51)] | 2004/03-2004/05 | Guangxi | South Central | Cross-sectional | Entertainment venues | 4 | English | 410 | 35 | 8.50% |  |  |  |  |  |  |  |  |  |  |
| Wu XT, 2005[[237](#_ENREF_237)] | 2004/04-2004/10 | Henan | South Central | Cross-sectional | Entertainment venues | 3 | Chinese | 465 | 4 | 0.86% |  |  |  |  |  |  |  |  |  |  |
| Yang BF, 2006a[[238](#_ENREF_238)] | 2004/08-2004/11 | Hubei | South Central | Cross-sectional | Entertainment venues | 2 | Chinese | 937 | 25 | 2.67% |  |  |  |  |  |  |  |  |  |  |
| Zhang WS, 2006a[[55](#_ENREF_55)] | 2004/10-2005/04 | Hubei | South Central | Cross-sectional | Entertainment venues | 4 | Chinese | 331 | 36 | 10.88% |  |  |  |  |  |  |  |  |  |  |
| Li WJ, 2007c[[239](#_ENREF_239)] | 2005/09 | Guangdong | South Central | Cross-sectional | Entertainment venues | 6 | Chinese | 240 | 2 | 0.83% |  |  |  |  |  |  |  |  |  |  |
| Li WJ, 2007a[[240](#_ENREF_240)] | 2005/11 | Guangdong | South Central | Cross-sectional | Entertainment venues | 6 | Chinese | 285 | 22 | 7.72% |  |  |  |  |  |  |  |  |  |  |
| Wang XX, 2008[[241](#_ENREF_241)] | 2006 - 2007 | Guangdong | South Central | Cross-sectional | Detaining education center | 6 | Chinese | 228 | 35 | 15.35% |  |  |  |  |  |  |  |  |  |  |
| Li Y, 2012[[59](#_ENREF_59)] | 2006/08 - 2007/01 | Guangdong | South Central | Cross-sectional | Entertainment venues | 6 | English | 318 | 18 | 5.70% |  |  |  |  |  |  |  |  |  |  |
| Li Y, 2009[[60](#_ENREF_60)] | 2006/08-2007/01 | Guangdong | South Central | Cross-sectional | Entertainment venues | 6 | Chinese | 320 | 26 | 8.13% |  |  |  |  |  |  |  |  |  |  |
| Wu ZZ, 2008[[61](#_ENREF_61)] | 2006/11-2007/01 | Guangdong | South Central | Cross-sectional | Entertainment venues | 1 | Chinese | 213 | 14 | 6.57% |  |  |  |  |  |  |  |  |  |  |
| Xu YF, 2009a[[242](#_ENREF_242)] | 2007 | Guangxi | South Central | National Sentinel Site | Entertainment venues | 7 | Chinese | 379 | 14 | 3.69% |  |  |  |  |  |  |  |  |  |  |
| Qin WW, 2011[[243](#_ENREF_243)] | 2007 | Guangxi | South Central | Cross-sectional | Entertainment venues | 4 | Chinese | 379 | 12 | 3.20% |  |  |  |  |  |  |  |  |  |  |
| Wang QQ, 2009a[[244](#_ENREF_244)] | 2007/01-2007/12 | Guangdong and Hainan | South Central | Cross-sectional | Entertainment venues | 3 | Chinese | 816 | 211 | 25.90% |  |  |  |  |  |  |  |  |  |  |
| Wang QQ, 2009c[[244](#_ENREF_244)] | 2007/01-2007/12 | Guangdong and Hainan | South Central | Cross-sectional | Entertainment venues | 3 | Chinese | 356 | 145 | 40.70% |  |  |  |  |  |  |  |  |  |  |
| Wang QQ, 2009c[[244](#_ENREF_244)] | 2007/01-2007/12 | Guangdong and Hainan | South Central | Cross-sectional | Entertainment venues | 3 | Chinese | 138 | 30 | 21.70% |  |  |  |  |  |  |  |  |  |  |
| Wang QQ, 2009e[[244](#_ENREF_244)] | 2007/01-2007/12 | Guangdong and Hainan | South Central | Cross-sectional | Entertainment venues | 3 | Chinese | 322 | 36 | 11.20% |  |  |  |  |  |  |  |  |  |  |
| Tan WW, 2011b[[245](#_ENREF_245)] | 2007/04 - 2007/06 | Guangxi | South Central | Cross-sectional | Entertainment venues | 4 | Chinese | 400 | 25 | 6.25% |  |  |  |  |  |  |  |  |  |  |
| Tan WW, 2011c[[245](#_ENREF_245)] | 2007/04 - 2007/06 | Guangxi | South Central | Cross-sectional | Entertainment venues | 4 | Chinese | 400 | 39 | 0.00% |  |  |  |  |  |  |  |  |  |  |
| Qin YM, 2010[[246](#_ENREF_246)] | 2007/04 - 2007/06 | Guangdong | South Central | Cross-sectional | Detaining education center | 4 | Chinese | 262 | 20 | 7.63% |  |  |  |  |  |  |  |  |  |  |
| Peng H, 2008[[247](#_ENREF_247)] | 2007/04-2007/10 | Guangdong | South Central | Cross-sectional | Entertainment venues | 3 | Chinese | 190 | 60 | 31.60% |  |  |  |  |  |  |  |  |  |  |
| Zhu BY, 2012[[62](#_ENREF_62)] | 2007/05-2007/10 | Guangxi | South Central | Cross-sectional | Entertainment venues | 6 | English | 488 | 35 | 7.17% |  |  |  |  |  |  |  |  |  |  |
| Yang P, 2009b[[248](#_ENREF_248)] | 2007/05-2007/12 | Guangdong | South Central | Cross-sectional | Entertainment venues | 1 | Chinese | 414 | 84 | 20.29% |  |  |  |  |  |  |  |  |  |  |
| Yang P, 2009c[[248](#_ENREF_248)] | 2007/05-2007/12 | Hainan | South Central | Cross-sectional | Entertainment venues | 1 | Chinese | 328 | 66 | 20.12% |  |  |  |  |  |  |  |  |  |  |
| Yang P, 2009d[[248](#_ENREF_248)] | 2007/05-2007/12 | Guangdong | South Central | Cross-sectional | Entertainment venues | 1 | Chinese | 127 | 48 | 37.80% |  |  |  |  |  |  |  |  |  |  |
| Yang P, 2009e[[248](#_ENREF_248)] | 2007/05-2007/12 | Guangdong | South Central | Cross-sectional | Entertainment venues | 1 | Chinese | 97 | 21 | 21.60% |  |  |  |  |  |  |  |  |  |  |
| Yang P, 2009f[[248](#_ENREF_248)] | 2007/05-2007/12 | Guangdong | South Central | Cross-sectional | Entertainment venues | 1 | Chinese | 190 | 15 | 7.90% |  |  |  |  |  |  |  |  |  |  |
| Yang P, 2009g[[248](#_ENREF_248)] | 2007/05-2007/12 | Hainan | South Central | Cross-sectional | Entertainment venues | 1 | Chinese | 102 | 39 | 38.20% |  |  |  |  |  |  |  |  |  |  |
| Yang P, 2009h[[248](#_ENREF_248)] | 2007/05-2007/12 | Hainan | South Central | Cross-sectional | Entertainment venues | 1 | Chinese | 44 | 9 | 20.50% |  |  |  |  |  |  |  |  |  |  |
| Yang P, 2009i[[248](#_ENREF_248)] | 2007/05-2007/12 | Hainan | South Central | Cross-sectional | Entertainment venues | 1 | Chinese | 182 | 18 | 9.90% |  |  |  |  |  |  |  |  |  |  |
| Tan WW, 2008[[249](#_ENREF_249)] | 2007/07-2007/09 | Guangxi | South Central | Cross-sectional | Entertainment venues | 4 | Chinese | 379 | 12 | 3.20% |  |  |  |  |  |  |  |  |  |  |
| Lin MH, 2008[[250](#_ENREF_250)] | 2007/08-2008/05 | Guangxi | South Central | Cross-sectional | Entertainment venues | 5 | Chinese | 236 | 36 | 15.25% |  |  |  |  |  |  |  |  |  |  |
| Qin WW, 2011[[243](#_ENREF_243)] | 2007-2009 | Guangxi | South Central | Cross-sectional | Entertainment venues | 4 | Chinese | 1,179 | 76 | 6.40% |  |  |  |  |  |  |  |  |  |  |
| Xu YF, 2009b[[242](#_ENREF_242)] | 2008 | Guangxi | South Central | National Sentinel Site | Entertainment venues | 7 | Chinese | 400 | 25 | 6.25% |  |  |  |  |  |  |  |  |  |  |
| Qin WW, 2011[[243](#_ENREF_243)] | 2008 | Guangxi | South Central | Cross-sectional | Entertainment venues | 4 | Chinese | 400 | 25 | 6.30% |  |  |  |  |  |  |  |  |  |  |
| Qin YM, 2010[[246](#_ENREF_246)] | 2008/04 - 2008/06 | Guangdong | South Central | Cross-sectional | Detaining education center | 4 | Chinese | 262 | 16 | 5.14% |  |  |  |  |  |  |  |  |  |  |
| Bai Y, 2009a[[251](#_ENREF_251)] | 2008/04-2008/07 | Guangxi | South Central | Cross-sectional | Entertainment venues | 6 | Chinese | 1,047 | 28 | 2.67% |  |  |  |  |  |  |  |  |  |  |
| Bai Y, 2009c[[251](#_ENREF_251)] | 2008/04-2008/07 | Guangxi | South Central | Cross-sectional | Entertainment venues | 6 | Chinese | 431 | 6 | 1.39% |  |  |  |  |  |  |  |  |  |  |
| Bai Y, 2009d[[251](#_ENREF_251)] | 2008/04-2008/07 | Guangxi | South Central | Cross-sectional | Entertainment venues | 6 | Chinese | 449 | 6 | 1.34% |  |  |  |  |  |  |  |  |  |  |
| Bai Y, 2009e[[251](#_ENREF_251)] | 2008/04-2008/07 | Guangxi | South Central | Cross-sectional | Entertainment venues | 6 | Chinese | 167 | 16 | 9.58% |  |  |  |  |  |  |  |  |  |  |
| Zhang YH, 2011[[131](#_ENREF_131)] | 2008/04-2008/07 | Guangdong | South Central | Cross-sectional | Others | 2 | Chinese | 289 | 10 | 3.50% |  |  |  |  |  |  |  |  |  |  |
| Zhang YH, 2011[[131](#_ENREF_131)] | 2008/04-2008/07 | Hubei | South Central | Cross-sectional | Others | 2 | Chinese | 444 | 16 | 3.60% |  |  |  |  |  |  |  |  |  |  |
| Zhang YH, 2011[[131](#_ENREF_131)] | 2008/04-2008/07 | Hainan | South Central | Cross-sectional | Others | 2 | Chinese | 400 | 28 | 7.00% |  |  |  |  |  |  |  |  |  |  |
| Zhang YH, 2011[[131](#_ENREF_131)] | 2008/04-2008/07 | Hainan | South Central | Cross-sectional | Others | 2 | Chinese | 404 | 36 | 8.90% |  |  |  |  |  |  |  |  |  |  |
| Zhao YY, 2010[[65](#_ENREF_65)] | 2008/10-2008/12 | Guangdong | South Central | Cross-sectional | Entertainment venues | 4 | Chinese | 622 | 28 | 4.50% |  |  |  |  |  |  |  |  |  |  |
| Qin WW, 2011[[243](#_ENREF_243)] | 2009 | Guangxi | South Central | Cross-sectional | Entertainment venues | 4 | Chinese | 400 | 39 | 9.80% |  |  |  |  |  |  |  |  |  |  |
| Lin RL, 2012[[252](#_ENREF_252)] | 2009 | Guangxi | South Central | Cross-sectional | Entertainment venues | 4 | Chinese | 400 | 16 | 4.00% |  |  |  |  |  |  |  |  |  |  |
| Ren XQ, 2011[[253](#_ENREF_253)] | 2009/01-2009/12 | Guangdong | South Central | Cross-sectional | Entertainment venues | 4 | Chinese | 7,137 | 247 | 3.46% |  |  |  |  |  |  |  |  |  |  |
| Ren XQ, 2011[[253](#_ENREF_253)] | 2009/01-2009/12 | Guangdong | South Central | Cross-sectional | Entertainment venues | 4 | Chinese | 6,050 | 157 | 2.60% |  |  |  |  |  |  |  |  |  |  |
| Ren XQ, 2011[[253](#_ENREF_253)] | 2009/01-2009/12 | Guangdong | South Central | Cross-sectional | Entertainment venues | 4 | Chinese | 774 | 57 | 7.36% |  |  |  |  |  |  |  |  |  |  |
| Ren XQ, 2011[[253](#_ENREF_253)] | 2009/01-2009/12 | Guangdong | South Central | Cross-sectional | Entertainment venues | 4 | Chinese | 313 | 33 | 10.54% |  |  |  |  |  |  |  |  |  |  |
| Xiang H, 2011[[254](#_ENREF_254)] | 2009/04-2009/06 | Hubei | South Central | Cross-sectional | Entertainment venues | 4 | Chinese | 400 | 14 | 3.50% |  |  |  |  |  |  |  |  |  |  |
| Xiang H, 2011[[254](#_ENREF_254)] | 2009/04-2009/06 | Hubei | South Central | Cross-sectional | Entertainment venues | 4 | Chinese | 146 | 1 | 0.68% |  |  |  |  |  |  |  |  |  |  |
| Xiang H, 2011[[254](#_ENREF_254)] | 2009/04-2009/06 | Hubei | South Central | Cross-sectional | Entertainment venues | 4 | Chinese | 135 | 4 | 2.96% |  |  |  |  |  |  |  |  |  |  |
| Xiang H, 2011[[254](#_ENREF_254)] | 2009/04-2009/06 | Hubei | South Central | Cross-sectional | Entertainment venues | 4 | Chinese | 129 | 9 | 6.98% |  |  |  |  |  |  |  |  |  |  |
| Zhong J, 2010a[[68](#_ENREF_68)] | 2009/06-2009/08 | Guangxi | South Central | Cross-sectional | Entertainment venues | 5 | Chinese | 406 | 19 | 4.68% |  |  |  |  |  |  |  |  |  |  |
| Zhong J, 2010c[[68](#_ENREF_68)] | 2009/06-2009/08 | Guangxi | South Central | Cross-sectional | Entertainment venues | 5 | Chinese | 80 | 4 | 5.00% |  |  |  |  |  |  |  |  |  |  |
| Zhong J, 2010d[[68](#_ENREF_68)] | 2009/06-2009/08 | Guangxi | South Central | Cross-sectional | Entertainment venues | 5 | Chinese | 165 | 4 | 2.42% |  |  |  |  |  |  |  |  |  |  |
| Zhong J, 2010e[[68](#_ENREF_68)] | 2009/06-2009/08 | Guangxi | South Central | Cross-sectional | Entertainment venues | 5 | Chinese | 161 | 11 | 6.83% |  |  |  |  |  |  |  |  |  |  |
| Zhong J, 2011[[67](#_ENREF_67)] | 2009/06-2009/08 | Guangxi | South Central | Cross-sectional | Entertainment venues | 4 | Chinese | 1,166 | 115 | 9.86% |  |  |  |  |  |  |  |  |  |  |
| Chen XS, 2012 (c)[[167](#_ENREF_167)] | 2009/06-2009/09 | Guangdong | South Central | Cross-sectional | Entertainment venues | 3 | English | 1,361 | 54 | 4.00% |  |  |  |  |  |  |  |  |  |  |
| Chen XS, 2012 (d)[[167](#_ENREF_167)] | 2009/06-2009/09 | Guangxi | South Central | Cross-sectional | Entertainment venues | 2 | English | 2,515 | 179 | 7.10% |  |  |  |  |  |  |  |  |  |  |
| Chen XS, 2012 (e)[[167](#_ENREF_167)] | 2009/06-2009/09 | Hainan | South Central | Cross-sectional | Entertainment venues | 3 | English | 1,451 | 56 | 3.90% |  |  |  |  |  |  |  |  |  |  |
| Zhao JZ, 2011[[69](#_ENREF_69)] | 2009/07-2009/12 | Hubei | South Central | Cross-sectional | Entertainment venues | 4 | Chinese | 165 | 14 | 8.50% |  |  |  |  |  |  |  |  |  |  |
| Wu ZZ, 2011[[255](#_ENREF_255)] | 2009/07-2010/01 | Guangdong | South Central | Cross-sectional | Entertainment venues | 6 | Chinese | 702 | 27 | 3.85% |  |  |  |  |  |  |  |  |  |  |
| Wu ZZ, 2011[[255](#_ENREF_255)] | 2009/07-2010/01 | Guangdong | South Central | Cross-sectional | Entertainment venues | 6 | Chinese | 702 | 27 | 3.85% |  |  |  |  |  |  |  |  |  |  |
| Wu ZZ, 2011[[255](#_ENREF_255)] | 2009/07-2010/01 | Guangdong | South Central | Cross-sectional | Entertainment venues | 6 | Chinese | 542 | 17 | 3.14% |  |  |  |  |  |  |  |  |  |  |
| Wu ZZ, 2011[[255](#_ENREF_255)] | 2009/07-2010/01 | Guangdong | South Central | Cross-sectional | Entertainment venues | 6 | Chinese | 67 | 4 | 5.97% |  |  |  |  |  |  |  |  |  |  |
| Wu ZZ, 2011[[255](#_ENREF_255)] | 2009/07-2010/01 | Guangdong | South Central | Cross-sectional | Entertainment venues | 6 | Chinese | 93 | 9 | 9.68% |  |  |  |  |  |  |  |  |  |  |
| Shi JX, 2012[[256](#_ENREF_256)] | 2009/09 | Guangdong | South Central | Cross-sectional | Entertainment venues | 4 | Chinese | 240 | 33 | 13.80% |  |  |  |  |  |  |  |  |  |  |
| Jing L, 2012a[[257](#_ENREF_257)] | 2009/10-2010/01 | Guangxi | South Central | Cross-sectional | Entertainment venues | 5 | English | 583 | 24 | 4.12% |  |  |  |  |  |  |  |  |  |  |
| Jing L, 2012b[[257](#_ENREF_257)] | 2009/10-2010/01 | Guangxi | South Central | Cross-sectional | Entertainment venues | 5 | English | 287 | 10 | 3.50% |  |  |  |  |  |  |  |  |  |  |
| Jing L, 2012c[[257](#_ENREF_257)] | 2009/10-2010/01 | Guangxi | South Central | Cross-sectional | Entertainment venues | 5 | English | 251 | 4 | 1.40% |  |  |  |  |  |  |  |  |  |  |
| Jing L, 2012d[[257](#_ENREF_257)] | 2009/10-2010/01 | Guangxi | South Central | Cross-sectional | Entertainment venues | 5 | English | 45 | 11 | 25.40% |  |  |  |  |  |  |  |  |  |  |
| Weir SS, 2012 (a)[[258](#_ENREF_258)] | 2009/10-2010/01 | Guangxi | South Central | Cross-sectional | Others | 6 | English | 538 | 46 | 8.55% |  |  |  |  |  |  |  |  |  |  |
| Weir SS, 2012 (b)[[258](#_ENREF_258)] | 2009/10-2010/01 | Guangxi | South Central | Cross-sectional | Others | 1 | English | 161 | 39 | 24.22% |  |  |  |  |  |  |  |  |  |  |
| Li J, 2012a[[259](#_ENREF_259)] | 2009/10-2010/01 | Guangxi | South Central | Cross-sectional | Entertainment venues | 6 | English | 287 | 10 | 3.50% |  |  |  |  |  |  |  |  |  |  |
| Li J, 2012b[[259](#_ENREF_259)] | 2009/10-2010/01 | Guangxi | South Central | Cross-sectional | Entertainment venues | 5 | English | 251 | 4 | 1.40% |  |  |  |  |  |  |  |  |  |  |
| Li J, 2012c[[259](#_ENREF_259)] | 2009/10-2010/01 | Guangxi | South Central | Cross-sectional | Entertainment venues | 6 | English | 45 | 11 | 25.40% |  |  |  |  |  |  |  |  |  |  |
| Zhou Y, 2012[[260](#_ENREF_260)] | 2009/12 | Guangxi | South Central | Cross-sectional | Entertainment venues | 4 | Chinese | 300 | 5 | 1.70% |  |  |  |  |  |  |  |  |  |  |
| Jiang N, 2012[[261](#_ENREF_261)] | 2009-2010 | Henan | South Central | Cross-sectional | Others | 3 | Chinese | 475 | 4 | 0.84% |  |  |  |  |  |  |  |  |  |  |
| Lin RL, 2012[[252](#_ENREF_252)] | 2009-2011 | Guangxi | South Central | Cross-sectional | Entertainment venues | 4 | Chinese | 1,200 | 64 | 5.33% |  |  |  |  |  |  |  |  |  |  |
| Huang KZ, 2010a[[262](#_ENREF_262)] | 2010 | Guangdong | South Central | Cross-sectional | Entertainment venues | 4 | Chinese | 391 | 11 | 1.81% |  |  |  |  |  |  |  |  |  |  |
| Chen FC, 2011[[263](#_ENREF_263)] | 2010 | Hubei | South Central | Cross-sectional | Entertainment venues | 3 | Chinese | 400 | 10 | 2.50% |  |  |  |  |  |  |  |  |  |  |
| Chen FC, 2011[[263](#_ENREF_263)] | 2010 | Hubei | South Central | Cross-sectional | Entertainment venues | 3 | Chinese | 36 | 0 | 0.00% |  |  |  |  |  |  |  |  |  |  |
| Chen FC, 2011[[263](#_ENREF_263)] | 2010 | Hubei | South Central | Cross-sectional | Entertainment venues | 3 | Chinese | 287 | 2 | 0.70% |  |  |  |  |  |  |  |  |  |  |
| Chen FC, 2011[[263](#_ENREF_263)] | 2010 | Hubei | South Central | Cross-sectional | Entertainment venues | 3 | Chinese | 77 | 8 | 10.39% |  |  |  |  |  |  |  |  |  |  |
| Liu XF, 2012[[264](#_ENREF_264)] | 2010 | Guangdong | South Central | Cross-sectional | Entertainment venues | 3 | Chinese | 613 | 4 | 0.65% |  |  |  |  |  |  |  |  |  |  |
| Lin RL, 2012[[252](#_ENREF_252)] | 2010 | Guangxi | South Central | Cross-sectional | Entertainment venues | 4 | Chinese | 400 | 13 | 3.25% |  |  |  |  |  |  |  |  |  |  |
| Xiong CS, 2012[[265](#_ENREF_265)] | 2010 | Hubei | South Central | Cross-sectional | Entertainment venues | 4 | Chinese | 400 | 1 | 0.25% |  |  |  |  |  |  |  |  |  |  |
| Zhu L, 2011[[70](#_ENREF_70)] | 2010/04-2010/06 | Hubei | South Central | Cross-sectional | National sentinel site | 4 | Chinese | 402 | 7 | 1.70% |  |  |  |  |  |  |  |  |  |  |
| Chen ZB, 2011[[266](#_ENREF_266)] | 2010/04-2010/07 | Guangdong | South Central | Cross-sectional | Entertainment venues | 3 | Chinese | 200 | 8 | 4.00% |  |  |  |  |  |  |  |  |  |  |
| Li Y, 2012[[267](#_ENREF_267)] | 2010-2011 | Hubei | South Central | Cross-sectional | Entertainment venues | 4 | Chinese | 400 | 12 | 3.00% |  |  |  |  |  |  |  |  |  |  |
| Tang MJ, 2012[[268](#_ENREF_268)] | 2011 | Guangxi | South Central | Cross-sectional | Entertainment venues | 4 | Chinese | 1,213 | 91 | 7.60% |  |  |  |  |  |  |  |  |  |  |
| Lin RL, 2012[[252](#_ENREF_252)] | 2011 | Guangxi | South Central | Cross-sectional | Entertainment venues | 4 | Chinese | 400 | 35 | 8.75% |  |  |  |  |  |  |  |  |  |  |
| Tang J, 2012[[269](#_ENREF_269)] | 2011 | Guangxi | South Central | Cross-sectional | Entertainment venues | 4 | Chinese | 400 | 7 | 1.75% |  |  |  |  |  |  |  |  |  |  |
| Ke XZ, 2012[[270](#_ENREF_270)] | 2011/04-2011/07 | Hubei | South Central | Cross-sectional | Others | 3 | Chinese | 40 | 17 | 4.25% |  |  |  |  |  |  |  |  |  |  |
| Quan XB, 2012[[271](#_ENREF_271)] | 2011/05-2011/07 | Guangxi | South Central | Cross-sectional | Entertainment venues | 4 | Chinese | 400 | 35 | 8.75% |  |  |  |  |  |  |  |  |  |  |
| Guo HJ, 2012[[272](#_ENREF_272)] |  | Guizhou | Southwest | Cross-sectional | Others | 4 | Chinese | 235 | 20 | 8.50% |  |  |  |  |  |  |  |  |  |  |
| Wu JW, 2002[[125](#_ENREF_125)] | 2000/10-2000/11 | Chongqing | Southwest | National Sentinel Site | Entertainment venues | 4 | Chinese | 103 | 5 | 4.85% |  |  |  |  |  |  |  |  |  |  |
| Wu Y, 2002a[[73](#_ENREF_73)] | 2001/09 | Chongqing | Southwest | Cohort | Entertainment venues | 6 | Chinese | 184 | 9 | 4.90% |  |  |  |  |  |  |  |  |  |  |
| Wu Y, 2002b[[73](#_ENREF_73)] | 2001/09 | Chongqing | Southwest | Cohort | Detaining education center | 6 | Chinese | 195 | 51 | 26.10% |  |  |  |  |  |  |  |  |  |  |
| Lei ZQ, 2005[[273](#_ENREF_273)] | 2004/04 | Sichuan | Southwest | Cross-sectional | Entertainment venues | 4 | Chinese | 259 | 15 | 5.79% |  |  |  |  |  |  |  |  |  |  |
| Xu SM, 2006[[274](#_ENREF_274)] | 2004/04-2004/11 | Chongqing | Southwest | National sentinel site | Detaining education center | 8 | Chinese | 1,113 | 61 | 5.50% |  |  |  |  |  |  |  |  |  |  |
| Wu Y, 2007a[[75](#_ENREF_75)] | 2004/09 | Chongqing | Southwest | Cohort | Entertainment venues | 4 | Chinese | 157 | 2 | 1.30% |  |  |  |  |  |  |  |  |  |  |
| Wu Y, 2007b[[75](#_ENREF_75)] | 2004/09 | Chongqing | Southwest | Cohort | Entertainment venues | 4 | Chinese | 169 | 25 | 14.80% |  |  |  |  |  |  |  |  |  |  |
| Ruan Y, 2006[[127](#_ENREF_127)] | 2004/12-2005/01 | Sichuan | Southwest | Cross-sectional | Entertainment venues | 5 | English | 343 | 54 | 15.70% |  |  |  |  |  |  |  |  |  |  |
| Choi SY, 2011[[275](#_ENREF_275)] | 2005 | NA | Southwest | Cross-sectional | Entertainment venues | 3 | English | 343 | 38 | 11.08% |  |  |  |  |  |  |  |  |  |  |
| Dai XQ, 2007[[76](#_ENREF_76)] | 2005/06-2005/09 | Chongqing | Southwest | Cross-sectional | Entertainment venues | 5 | Chinese | 341 | 36 | 11.90% |  |  |  |  |  |  |  |  |  |  |
| Tan XJ, 2007[[77](#_ENREF_77)] | 2005/08-2005/09 | Chongqing | Southwest | Community-based cross-sectional | Entertainment venues | 5 | Chinese | 266 | 7 | 2.60% |  |  |  |  |  |  |  |  |  |  |
| Ding XB, 2006[[78](#_ENREF_78)] | 2005/08-2005/09 | Chongqing | Southwest | Cross-sectional | Entertainment venues | 4 | Chinese | 519 | 11 | 2.10% |  |  |  |  |  |  |  |  |  |  |
| Li DM, 2007a[[276](#_ENREF_276)] | 2006 | Guizhou | Southwest | Cross-sectional | Entertainment venues | 7 | Chinese | 432 | 56 | 12.96% |  |  |  |  |  |  |  |  |  |  |
| Li DM, 2007b[[276](#_ENREF_276)] | 2006 | Guizhou | Southwest | Cross-sectional | Entertainment venues | 7 | Chinese | 220 | 7 | 3.18% |  |  |  |  |  |  |  |  |  |  |
| Li DM, 2007d[[276](#_ENREF_276)] | 2006 | Guizhou | Southwest | Cross-sectional | Entertainment venues | 7 | Chinese | 212 | 49 | 23.11% |  |  |  |  |  |  |  |  |  |  |
| Zheng QC, 2012[[277](#_ENREF_277)] | 2006 | Chongqing | Southwest | Cross-sectional | Entertainment venues | 4 | Chinese | 217 | 8 | 3.69% |  |  |  |  |  |  |  |  |  |  |
| Wang HB, 2010a[[79](#_ENREF_79)] | 2006/03 | Yunnan | Southwest | Cross-sectional | Entertainment venues | 7 | English | 737 | 55 | 8.40% |  |  |  |  |  |  |  |  |  |  |
| Xu J, 2011[[80](#_ENREF_80)] | 2006/03 - 2007/04 | Yunnan | Southwest | Cohort | Others | 3 | English | 1,642 | 136 | 8.28% |  |  |  |  |  |  |  |  |  |  |
| Wang H, 2012[[81](#_ENREF_81)] | 2006/03 - 2009/11 | Yunnan | Southwest | Cohort | Entertainment venues | 6 | English | 851 | 67 | 7.90% |  |  |  |  |  |  |  |  |  |  |
| Xu JJ, 2008[[83](#_ENREF_83)] | 2006/03-2006/04 | Yunnan | Southwest | Cross-sectional | Entertainment venues | 6 | English | 96 | 12 | 12.50% |  |  |  |  |  |  |  |  |  |  |
| Wang H, 2009[[84](#_ENREF_84)] | 2006/03-2006/05 | Yunnan | Southwest | Cross-sectional | Entertainment venues | 4 | English | 737 | 55 | 7.46% |  |  |  |  |  |  |  |  |  |  |
| Wang H, 2009e[[84](#_ENREF_84)] | 2006/03-2006/05 | Yunnan | Southwest | Cross-sectional | Entertainment venues | 4 | English | 458 | 28 | 6.10% |  |  |  |  |  |  |  |  |  |  |
| Wang H, 2009f[[84](#_ENREF_84)] | 2006/03-2006/05 | Yunnan | Southwest | Cross-sectional | Entertainment venues | 4 | English | 279 | 27 | 9.70% |  |  |  |  |  |  |  |  |  |  |
| Wang HB, 2010a[[79](#_ENREF_79)] | 2006/03-2006/12 | Yunnan | Southwest | Cross-sectional | Entertainment venues | 6 | English | 1,484 | 118 | 7.95% |  |  |  |  |  |  |  |  |  |  |
| Wang H, 2011[[85](#_ENREF_85)] | 2006/03-2009/11 | Yunnan | Southwest | Cohort | Entertainment venues | 6 | English | 751 | 56 | 7.50% |  |  |  |  |  |  |  |  |  |  |
| Wang H, 2011[[86](#_ENREF_86)] | 2006/03-2009/11 | Yunnan | Southwest | Cohort | Entertainment venues | 4 | English | 1,866 | 101 | 5.41% |  |  |  |  |  |  |  |  |  |  |
| Luo XR, 2008a[[87](#_ENREF_87)] | 2006/05-2006/06 | Sichuan | Southwest | Cross-sectional | Entertainment venues | 5 | Chinese | 216 | 15 | 6.94% |  |  |  |  |  |  |  |  |  |  |
| Li QH, 2009a[[88](#_ENREF_88)] | 2006/09-2006/10 | Yunnan | Southwest | Cross-sectional | Entertainment venues | 7 | Chinese | 335 | 19 | 5.67% |  |  |  |  |  |  |  |  |  |  |
| Li QH, 2009b[[88](#_ENREF_88)] | 2006/09-2006/10 | Yunnan | Southwest | Cross-sectional | Entertainment venues | 7 | Chinese | 312 | 23 | 7.37% |  |  |  |  |  |  |  |  |  |  |
| Li QH, 2009c[[88](#_ENREF_88)] | 2006/09-2006/10 | Yunnan | Southwest | Cross-sectional | Entertainment venues | 7 | Chinese | 87 | 20 | 22.99% |  |  |  |  |  |  |  |  |  |  |
| Wang GX, 2008a[[89](#_ENREF_89)] | 2006/09-2006/10 | Yunnan | Southwest | Cross-sectional | Entertainment venues | 5 | Chinese | 747 | 63 | 8.43% |  |  |  |  |  |  |  |  |  |  |
| Wang GX, 2008b[[89](#_ENREF_89)] | 2006/09-2006/10 | Yunnan | Southwest | Cross-sectional | Entertainment venues | 5 | Chinese | 266 | 7 | 2.63% |  |  |  |  |  |  |  |  |  |  |
| Wang GX, 2008c[[89](#_ENREF_89)] | 2006/09-2006/10 | Yunnan | Southwest | Cross-sectional | Entertainment venues | 5 | Chinese | 351 | 27 | 7.69% |  |  |  |  |  |  |  |  |  |  |
| Wang GX, 2008d[[89](#_ENREF_89)] | 2006/09-2006/10 | Yunnan | Southwest | Cross-sectional | Entertainment venues | 5 | Chinese | 120 | 21 | 17.50% |  |  |  |  |  |  |  |  |  |  |
| Wang HB, 2010b[[79](#_ENREF_79)] | 2006/10 | Yunnan | Southwest | Cross-sectional | Entertainment venues | 4 | English | 747 | 63 | 8.40% |  |  |  |  |  |  |  |  |  |  |
| Zheng QC, 2012[[277](#_ENREF_277)] | 2006-2009 | Chongqing | Southwest | Cross-sectional | Entertainment venues | 4 | Chinese | 420 | 19 | 4.52% |  |  |  |  |  |  |  |  |  |  |
| Gao LL, 2008a[[90](#_ENREF_90)] | 2007 | Yunnan | Southwest | Cross-sectional | Detaining education center | 4 | Chinese | 270 | 32 | 11.90% |  |  |  |  |  |  |  |  |  |  |
| De QZG, 2009[[278](#_ENREF_278)] | 2007 - 2008 | Tibet | Southwest | Cross-sectional | Others | 5 | Chinese | 204 | 3 | 1.47% |  |  |  |  |  |  |  |  |  |  |
| Xu JJ, 2012[[91](#_ENREF_91)] | 2007/03-2007-07 | Yunnan | Southwest | Cross-sectional | Entertainment venues | 6 | English | 705 | 62 | 8.80% |  |  |  |  |  |  |  |  |  |  |
| Wang HB, 2010c[[79](#_ENREF_79)] | 2007/05 | Yunnan | Southwest | Cross-sectional | Entertainment venues | 3 | English | 705 | 62 | 8.80% |  |  |  |  |  |  |  |  |  |  |
| Wang HB, 2010b[[79](#_ENREF_79)] | 2007/05 | Yunnan | Southwest |  | Others | 6 | English | 705 | 62 | 8.79% |  |  |  |  |  |  |  |  |  |  |
| Yao Y, 2012[[92](#_ENREF_92)] | 2007/09-2007/10 | Yunnan | Southwest | Cross-sectional | Entertainment venues | 7 | English | 397 | 27 | 6.80% |  |  |  |  |  |  |  |  |  |  |
| Li WZ, 2009a[[279](#_ENREF_279)] | 2008 | Yunnan | Southwest | Cross-sectional | Entertainment venues | 6 | Chinese | 405 | 6 | 1.48% |  |  |  |  |  |  |  |  |  |  |
| Zhang YH, 2011[[131](#_ENREF_131)] | 2008/04-2008/07 | Chongqing | Southwest | Cross-sectional | Others | 2 | Chinese | 429 | 5 | 1.20% |  |  |  |  |  |  |  |  |  |  |
| Zhang YH, 2011[[131](#_ENREF_131)] | 2008/04-2008/07 | Yunnan | Southwest | Cross-sectional | Others | 2 | Chinese | 405 | 6 | 1.50% |  |  |  |  |  |  |  |  |  |  |
| Chen C, 2010[[280](#_ENREF_280)] | 2009-2010 | Sichuan | Southwest | Cross-sectional | Entertainment venues | 4 | Chinese | 403 | 28 | 6.95% |  |  |  |  |  |  |  |  |  |  |
| Zhang XD, 2012[[95](#_ENREF_95)] | 2010/07-2011/02 | Yunnan | Southwest | Cross-sectional | Entertainment venues | 6 | English | 201 | 3 | 1.50% |  |  |  |  |  |  |  |  |  |  |
| Zhang H, 2012[[281](#_ENREF_281)] | 2011 | Yunnan | Southwest | Cross-sectional | Others | 2 | Chinese | 1,301 | 2 | 0.15% |  |  |  |  |  |  |  |  |  |  |
| Shao ZP, 2012[[96](#_ENREF_96)] | 2011/05-2011/06 | Chongqing | Southwest | Cross-sectional | Entertainment venues | 4 | Chinese | 318 | 20 | 6.29% |  |  |  |  |  |  |  |  |  |  |
| d)Trichomonas vaginitis |  |  |  |  |  |  |  |  |  |  |  |  |  |  |  |  |  |  |  |  |
| Wei CQ, 2007[[1](#_ENREF_1)] | 2006 |  |  | Cross-sectional | Entertainment venues | 3 | Chinese | 280 | 13 | 4.64% |  |  |  |  |  |  |  |  |  |  |
| Zheng BZ, 2003[[4](#_ENREF_4)] | 2000/06 | Zhejiang | East | Cross-sectional | Entertainment venues | 5 | Chinese | 176 | 15 | 8.52% |  |  |  |  |  |  |  |  |  |  |
| Xu LL, 2006a[[8](#_ENREF_8)] | 2003/04-2004/10 | Shanghai | East | Cross-sectional | Entertainment venues | 4 | Chinese | 191 | 3 | 1.60% |  |  |  |  |  |  |  |  |  |  |
| Lin YQ, 2005[[9](#_ENREF_9)] | 2003/09 | Zhejiang | East | Cross-sectional | Detaining education center | 2 | Chinese | 181 | 22 | 12.15% |  |  |  |  |  |  |  |  |  |  |
| Wang HY, 2005[[143](#_ENREF_143)] | 2004/03-2004/12 | Shandong | East | Cross-sectional | Others | 4 | Chinese | 366 | 27 | 7.40% |  |  |  |  |  |  |  |  |  |  |
| Song SJ, 2008[[101](#_ENREF_101)] | 2006/01-2006/12 | Zhejiang | East | Cross-sectional | Entertainment venues | 3 | Chinese | 272 | 59 | 21.93% |  |  |  |  |  |  |  |  |  |  |
| Wang WM, 2008[[20](#_ENREF_20)] | 2007/01-2007/12 | Jiangsu | East | Cross-sectional | Detaining education center | 4 | Chinese | 297 | 20 | 6.73% |  |  |  |  |  |  |  |  |  |  |
| Zhang Y, 2005[[11](#_ENREF_11)] | 2004/04-2004/05 | Shandong | East | Cross-sectional | Entertainment venues | 3 | Chinese | 180 | 6 | 3.33% |  |  |  |  |  |  |  |  |  |  |
| Xu lq, 2008[[282](#_ENREF_282)] | 2007/06-2007/07 | Jiangsu | East | Cross-sectional | Entertainment venues | 2 | Chinese | 115 | 3 | 2.60% |  |  |  |  |  |  |  |  |  |  |
| Yang JY, 2011[[21](#_ENREF_21)] | 2007/10-2007/11 | Jiangsu | East | Cross-sectional | Entertainment venues | 4 | Chinese | 247 | 4 | 1.62% |  |  |  |  |  |  |  |  |  |  |
| Zhao YQ, 2010a[[23](#_ENREF_23)] | 2008/04-2008/05 | Jiangsu | East | Cross-sectional | Entertainment venues | 4 | Chinese | 396 | 14 | 3.50% |  |  |  |  |  |  |  |  |  |  |
| Shao MC, 2010[[106](#_ENREF_106)] | 2009/04 | Jiangsu | East | Cross-sectional | Entertainment venues | 1 | Chinese | 402 | 2 | 0.50% |  |  |  |  |  |  |  |  |  |  |
| Wei XW, 2002[[111](#_ENREF_111)] | 2001/07-2001/08 | Xinjiang | Northwest | Cross-sectional | Entertainment venues | 3 | Chinese | 181 | 3 | 1.66% |  |  |  |  |  |  |  |  |  |  |
| Wei XW, 2005[[34](#_ENREF_34)] | 2003/03-2003/05 | Xinjiang | Northwest | Cross-sectional | Entertainment venues | 2 | Chinese | 459 | 31 | 6.75% |  |  |  |  |  |  |  |  |  |  |
| Li F, 2006[[35](#_ENREF_35)] | 2004/04-2004/06 | Xinjiang | Northwest | Cross-sectional | Entertainment venues | 6 | Chinese | 611 | 37 | 6.06% |  |  |  |  |  |  |  |  |  |  |
| Song Y, 2006[[37](#_ENREF_37)] | 2006 | Xinjiang | Northwest | Cross-sectional | Entertainment venues | 4 | Chinese | 103 | 14 | 13.59% |  |  |  |  |  |  |  |  |  |  |
| Zhang CJ, 2001[[41](#_ENREF_41)] | 2000/03-2000/08 | Henan | South Central | Cross-sectional | Detaining education center | 3 | Chinese | 65 | 16 | 24.66% |  |  |  |  |  |  |  |  |  |  |
| Sun J, 2003[[113](#_ENREF_113)] | 2001/09-2001/12 | Henan | South Central | Cross-sectional | Entertainment venues | 2 | Chinese | 220 | 7 | 3.18% |  |  |  |  |  |  |  |  |  |  |
| Luo J, 2005c[[46](#_ENREF_46)] | 2003/06 | Guangxi | South Central | Cross-sectional | Entertainment venues | 4 | Chinese | 126 | 2 | 1.59% |  |  |  |  |  |  |  |  |  |  |
| Luo J, 2005a[[46](#_ENREF_46)] | 2003/06 | Guangxi | South Central | Cross-sectional | Entertainment venues | 4 | Chinese | 278 | 11 | 3.96 |  |  |  |  |  |  |  |  |  |  |
| Luo J, 2005e[[46](#_ENREF_46)] | 2003/06 | Guangxi | South Central | Cross-sectional | Entertainment venues | 4 | Chinese | 152 | 9 | 5.92 |  |  |  |  |  |  |  |  |  |  |
| Hong Y, 2009[[50](#_ENREF_50)] | 2004 | Guangxi | South Central | Cross-sectional | Entertainment venues | 4 | English | 411 | 30 | 7.40% |  |  |  |  |  |  |  |  |  |  |
| Wang H, 2005[[51](#_ENREF_51)] | 2004/03-2004/05 | Guangxi | South Central | Cross-sectional | Entertainment venues | 4 | English | 410 | 29 | 7.10% |  |  |  |  |  |  |  |  |  |  |
| Zhou YJ, 2007[[52](#_ENREF_52)] | 2004/05 | Guangxi | South Central | Cross-sectional | Entertainment venues | 6 | Chinese | 80 | 5 | 6.30% |  |  |  |  |  |  |  |  |  |  |
| Li CG, 2007a[[58](#_ENREF_58)] | 2006/05 | Guangdong | South Central | Cross-sectional | Entertainment venues | 6 | Chinese | 522 | 36 | 6.90% |  |  |  |  |  |  |  |  |  |  |
| Li XF, 2008a[[121](#_ENREF_121)] | 2007/01-2007/03 | Hunan | South Central | Cross-sectional | Entertainment venues | 4 | Chinese | 142 | 53 | 23.60% |  |  |  |  |  |  |  |  |  |  |
| Chen ZH, 2007a[[71](#_ENREF_71)] | N/A | Guangdong | South Central | Cross-sectional | Entertainment venues | 3 | Chinese | 1,200 | 20 | 1.67% |  |  |  |  |  |  |  |  |  |  |
| Yang P, 2006[[72](#_ENREF_72)] |  | Sichuan | Southwest | Cross-sectional | Entertainment venues | 2 | Chinese | 407 | 25 | 6.20% |  |  |  |  |  |  |  |  |  |  |
| Wu Y, 2001b[[283](#_ENREF_283)] | 2000/03-2001/03 | Chongqing | Southwest | Cohort | Entertainment venues | 3 | Chinese | 636 | 0 | 0.00% |  |  |  |  |  |  |  |  |  |  |
| Wu Y, 2001d[[283](#_ENREF_283)] | 2000/03-2001/03 | Chongqing | Southwest | Cohort | Detaining education center | 3 | Chinese | 415 | 99 | 23.86% |  |  |  |  |  |  |  |  |  |  |
| Wu Y, 2001f[[283](#_ENREF_283)] | 2000/03-2001/03 | Chongqing | Southwest | Cohort | Detaining education center | 3 | Chinese | 636 | 0 | 0.00% |  |  |  |  |  |  |  |  |  |  |
| Wu Y, 2002a[[73](#_ENREF_73)] | 2001/09 | Chongqing | Southwest | Cohort | Entertainment venues | 6 | Chinese | 184 | 0 | 0.00% |  |  |  |  |  |  |  |  |  |  |
| Wu Y, 2002b[[73](#_ENREF_73)] | 2001/09 | Chongqing | Southwest | Cohort | Detaining education center | 6 | Chinese | 195 | 103 | 52.80% |  |  |  |  |  |  |  |  |  |  |
| Yang HW, 2003a[[284](#_ENREF_284)] | 2002/04-2002/05 | Sichuan | Southwest | Cross-sectional | Entertainment venues | 2 | Chinese | 126 | 10 | 12.35% |  |  |  |  |  |  |  |  |  |  |
| Wang QQ, 2005[[74](#_ENREF_74)] | 2004/02 | Sichuan | Southwest | Cross-sectional | Entertainment venues | 3 | Chinese | 206 | 21 | 10.20% |  |  |  |  |  |  |  |  |  |  |
| Dai XQ, 2007[[76](#_ENREF_76)] | 2005/06-2005/09 | Chongqing | Southwest | Cross-sectional | Entertainment venues | 5 | Chinese | 341 | 82 | 27.20% |  |  |  |  |  |  |  |  |  |  |
| Wang HB, 2010a[[79](#_ENREF_79)] | 2006/03 | Yunnan | Southwest | Cross-sectional | Entertainment venues | 7 | English | 737 | 78 | 10.60% |  |  |  |  |  |  |  |  |  |  |
| Xu J, 2011[[80](#_ENREF_80)] | 2006/03 - 2007/04 | Yunnan | Southwest | Cohort | Others | 3 | English | 1,642 | 179 | 10.90% |  |  |  |  |  |  |  |  |  |  |
| Li QH, 2010a[[82](#_ENREF_82)] | 2006/03-2006/04 | Yunnan | Southwest | Cross-sectional | Entertainment venues | 6 | Chinese | 716 | 78 | 10.90% |  |  |  |  |  |  |  |  |  |  |
| Xu JJ, 2008[[83](#_ENREF_83)] | 2006/03-2006/04 | Yunnan | Southwest | Cross-sectional | Entertainment venues | 6 | English | 96 | 21 | 22.10% |  |  |  |  |  |  |  |  |  |  |
| Wang H, 2009[[84](#_ENREF_84)] | 2006/03-2006/05 | Yunnan | Southwest | Cross-sectional | Entertainment venues | 4 | English | 737 | 78 | 10.58% |  |  |  |  |  |  |  |  |  |  |
| Wang H, 2009e[[84](#_ENREF_84)] | 2006/03-2006/05 | Yunnan | Southwest | Cross-sectional | Entertainment venues | 4 | English | 458 | 47 | 10.30% |  |  |  |  |  |  |  |  |  |  |
| Wang H, 2009f[[84](#_ENREF_84)] | 2006/03-2006/05 | Yunnan | Southwest | Cross-sectional | Entertainment venues | 4 | English | 279 | 31 | 11.10% |  |  |  |  |  |  |  |  |  |  |
| Wang HB, 2010a[[79](#_ENREF_79)] | 2006/03-2006/12 | Yunnan | Southwest | Cross-sectional | Entertainment venues | 6 | English | 1,484 | 57 | 9.70% |  |  |  |  |  |  |  |  |  |  |
| Wang H, 2011[[85](#_ENREF_85)] | 2006/03-2009/11 | Yunnan | Southwest | Cohort | Entertainment venues | 6 | English | 742 | 54 | 7.30% |  |  |  |  |  |  |  |  |  |  |
| Luo XR, 2008a[[87](#_ENREF_87)] | 2006/05-2006/06 | Sichuan | Southwest | Cross-sectional | Entertainment venues | 5 | Chinese | 194 | 18 | 9.28% |  |  |  |  |  |  |  |  |  |  |
| Li QH, 2009a[[88](#_ENREF_88)] | 2006/09-2006/10 | Yunnan | Southwest | Cross-sectional | Entertainment venues | 7 | Chinese | 335 | 28 | 8.36% |  |  |  |  |  |  |  |  |  |  |
| Li QH, 2009b[[88](#_ENREF_88)] | 2006/09-2006/10 | Yunnan | Southwest | Cross-sectional | Entertainment venues | 7 | Chinese | 312 | 28 | 8.97% |  |  |  |  |  |  |  |  |  |  |
| Li QH, 2009c[[88](#_ENREF_88)] | 2006/09-2006/10 | Yunnan | Southwest | Cross-sectional | Entertainment venues | 7 | Chinese | 87 | 10 | 11.49% |  |  |  |  |  |  |  |  |  |  |
| Wang GX, 2008b[[89](#_ENREF_89)] | 2006/09-2006/10 | Yunnan | Southwest | Cross-sectional | Entertainment venues | 5 | Chinese | 266 | 24 | 9.02% |  |  |  |  |  |  |  |  |  |  |
| Wang GX, 2008c[[89](#_ENREF_89)] | 2006/09-2006/10 | Yunnan | Southwest | Cross-sectional | Entertainment venues | 5 | Chinese | 351 | 38 | 10.83% |  |  |  |  |  |  |  |  |  |  |
| Wang GX, 2008d[[89](#_ENREF_89)] | 2006/09-2006/10 | Yunnan | Southwest | Cross-sectional | Entertainment venues | 5 | Chinese | 120 | 16 | 13.33% |  |  |  |  |  |  |  |  |  |  |
| Wang HB, 2010b[[79](#_ENREF_79)] | 2006/10 | Yunnan | Southwest | Cross-sectional | Entertainment venues | 3 | English | 747 | 66 | 8.80% |  |  |  |  |  |  |  |  |  |  |
| Yang X, 2009a[[129](#_ENREF_129)] | 2007 | Yunnan | Southwest | Cross-sectional | Entertainment venues | 1 | Chinese | 371 | 165 | 44.50% |  |  |  |  |  |  |  |  |  |  |
| Gao LL, 2008a[[90](#_ENREF_90)] | 2007 | Yunnan | Southwest | Cross-sectional | Entertainment venues | 4 | Chinese | 270 | 15 | 5.60% |  |  |  |  |  |  |  |  |  |  |
| Yang X, 2009f[[129](#_ENREF_129)] | 2007 | Yunnan | Southwest | Cross-sectional | Entertainment venues | 1 | Chinese | 211 | 93 | 44.10% |  |  |  |  |  |  |  |  |  |  |
| Yang X, 2009k[[129](#_ENREF_129)] | 2007 | Yunnan | Southwest | Cross-sectional | Entertainment venues | 1 | Chinese | 109 | 50 | 45.90% |  |  |  |  |  |  |  |  |  |  |
| Yang X, 2009p[[129](#_ENREF_129)] | 2007 | Yunnan | Southwest | Cross-sectional | Entertainment venues | 1 | Chinese | 51 | 22 | 43.10% |  |  |  |  |  |  |  |  |  |  |
| Yang X, 2009b[[129](#_ENREF_129)] | 2007 | Yunnan | Southwest | Cross-sectional | Entertainment venues | 1 | Chinese | 371 | 36 | 9.70% |  |  |  |  |  |  |  |  |  |  |
| Yang X, 2009g[[129](#_ENREF_129)] | 2007 | Yunnan | Southwest | Cross-sectional | Entertainment venues | 1 | Chinese | 211 | 19 | 9.00% |  |  |  |  |  |  |  |  |  |  |
| Yang X, 2009l[[129](#_ENREF_129)] | 2007 | Yunnan | Southwest | Cross-sectional | Entertainment venues | 1 | Chinese | 109 | 9 | 8.30% |  |  |  |  |  |  |  |  |  |  |
| Yang X, 2009q[[129](#_ENREF_129)] | 2007 | Yunnan | Southwest | Cross-sectional | Entertainment venues | 1 | Chinese | 51 | 8 | 15.70% |  |  |  |  |  |  |  |  |  |  |
| Xu JJ, 2012[[91](#_ENREF_91)] | 2007/03-2007-07 | Yunnan | Southwest | Cross-sectional | Entertainment venues | 6 | English | 705 | 58 | 8.20% |  |  |  |  |  |  |  |  |  |  |
| Wang HB, 2010c[[79](#_ENREF_79)] | 2007/05 | Yunnan | Southwest | Cross-sectional | Entertainment venues | 2 | English | 705 | 57 | 8.10% |  |  |  |  |  |  |  |  |  |  |
| Wang HB, 2010b[[79](#_ENREF_79)] | 2007/05 | Yunnan | Southwest |  | Others | 6 | English | 705 | 57 | 8.09% |  |  |  |  |  |  |  |  |  |  |
| e) Genital Warts |  |  |  |  |  |  |  |  |  |  |  |  |  |  |  |  |  |  |  |  |
| Wei CQ, 2007[[1](#_ENREF_1)] | 2006 |  |  | Cross-sectional | Entertainment venues | 3 | Chinese | 280 | 49 | 17.50% |  |  |  |  |  |  |  |  |  |  |
| Zhang ZX, 2009[[3](#_ENREF_3)] |  | Fujian | East | Cross-sectional | Others | 2 | Chinese | 128 | 5 | 3.90% |  |  |  |  |  |  |  |  |  |  |
| Liu S, 2008[[285](#_ENREF_285)] | 11/2006/11 | Shandong | East | Cross-sectional | Entertainment venues | 3 | Chinese | 309 | 4 | 0.97% |  |  |  |  |  |  |  |  |  |  |
| Zheng BZ, 2003[[4](#_ENREF_4)] | 2000/06 | Zhejiang | East | Cross-sectional | Entertainment venues | 5 | Chinese | 176 | 1 | 0.57% |  |  |  |  |  |  |  |  |  |  |
| Du YP, 2004a[[5](#_ENREF_5)] | 2001/11 | Jiangsu | East | Cross-sectional | Entertainment venues | 5 | Chinese | 303 | 7 | 2.30% |  |  |  |  |  |  |  |  |  |  |
| Yang P, 2005a[[7](#_ENREF_7)] | 2002/12 | Fujian | East | Cross-sectional | Entertainment venues | 3 | Chinese | 149 | 1 | 0.67% |  |  |  |  |  |  |  |  |  |  |
| Xu LL, 2006a[[8](#_ENREF_8)] | 2003/04-2004/10 | Shanghai | East | Cross-sectional | Entertainment venues | 4 | Chinese | 191 | 4 | 2.10% |  |  |  |  |  |  |  |  |  |  |
| Li L, 2003[[99](#_ENREF_99)] | 2001/11-2001/12 | Jiangsu | East | Cross-sectional | Entertainment venues | 4 | Chinese | 288 | 7 | 2.43% |  |  |  |  |  |  |  |  |  |  |
| Lin YQ, 2005[[9](#_ENREF_9)] | 2003/09 | Zhejiang | East | Cross-sectional | Detaining education center | 4 | Chinese | 181 | 6 | 3.31% |  |  |  |  |  |  |  |  |  |  |
| Zhang Y, 2005[[11](#_ENREF_11)] | 2004/04-2004/05 | Shandong | East | Cross-sectional | Entertainment venues | 3 | Chinese | 180 | 11 | 6.10% |  |  |  |  |  |  |  |  |  |  |
| Qi HZ, 2007[[100](#_ENREF_100)] | 2005/01-2005/12 | Jiangsu | East | Cross-sectional | Entertainment venues | 4 | Chinese | 207 | 9 | 4.35% |  |  |  |  |  |  |  |  |  |  |
| Xu HQ, 2008[[15](#_ENREF_15)] | 2006 | Zhejiang | East | Cross-sectional | Entertainment venues | 5 | Chinese | 84 | 1 | 1.20% |  |  |  |  |  |  |  |  |  |  |
| Song SJ, 2008[[101](#_ENREF_101)] | 2006/01-2006/12 | Zhejiang | East | Cross-sectional | Entertainment venues | 3 | Chinese | 272 | 2 | 0.74% |  |  |  |  |  |  |  |  |  |  |
| Zhu FG, 2009a[[19](#_ENREF_19)] | 2006/05 | Jiangsu | East | Cross-sectional | Entertainment venues | 4 | Chinese | 252 | 5 | 1.80% |  |  |  |  |  |  |  |  |  |  |
| Wang WM, 2008[[20](#_ENREF_20)] | 2007/01-2007/12 | Jiangsu | East | Cross-sectional | Detaining education center | 4 | Chinese | 297 | 40 | 13.47% |  |  |  |  |  |  |  |  |  |  |
| Xu lq, 2008[[282](#_ENREF_282)] | 2007/06-2007/07 | Jiangsu | East | Cross-sectional | Entertainment venues | 2 | Chinese | 115 | 1 | 0.90% |  |  |  |  |  |  |  |  |  |  |
| Yang JY, 2011[[21](#_ENREF_21)] | 2007/10-2007/11 | Jiangsu | East | Cross-sectional | Entertainment venues | 4 | Chinese | 247 | 2 | 0.81% |  |  |  |  |  |  |  |  |  |  |
| Zhao YQ, 2010a[[23](#_ENREF_23)] | 2008/04-2008/05 | Jiangsu | East | Cross-sectional | Entertainment venues | 4 | Chinese | 396 | 24 | 6.10% |  |  |  |  |  |  |  |  |  |  |
| Wang J, 2008a[[103](#_ENREF_103)] | 2008/06 | Jiangsu | East | Cross-sectional | Entertainment venues | 4 | Chinese | 400 | 13 | 3.25% |  |  |  |  |  |  |  |  |  |  |
| Chen JQ, 2009[[104](#_ENREF_104)] | 2008/10-2008/11 | Zhejiang | East | Cross-sectional | Entertainment venues | 3 | Chinese | 111 | 1 | 0.90% |  |  |  |  |  |  |  |  |  |  |
| Tang ZL, 2011[[28](#_ENREF_28)] | 2010/03-2010/07 | Shandong | East | Cross-sectional | Entertainment venues | 4 | Chinese | 106 | 23 | 21.70% |  |  |  |  |  |  |  |  |  |  |
| Tang ZL, 2011[[28](#_ENREF_28)] | 2010/03-2010/07 | Shandong | East | Cross-sectional | Entertainment venues | 4 | Chinese | 216 | 40 | 18.50% |  |  |  |  |  |  |  |  |  |  |
| Li JE, 2012[[108](#_ENREF_108)] | 2008/03 | Hebei | North | Cross-sectional | Entertainment venues | 2 | Chinese | 370 | 8 | 2.16% |  |  |  |  |  |  |  |  |  |  |
| Zhang XY, 2012[[109](#_ENREF_109)] | 2010/12-2011/12 | Inner Mongolia | North | Cross-sectional | Entertainment venues | 4 | Chinese | 247 | 3 | 1.20% |  |  |  |  |  |  |  |  |  |  |
| Li Y, 2011[[31](#_ENREF_31)] | 2010 | Heilongjiang | Northeast | Cross-sectional | National sentinel site | 4 | Chinese | 7,662 | 168 | 2.20% |  |  |  |  |  |  |  |  |  |  |
| Lin L, 2002b[[110](#_ENREF_110)] | 2000/08-2000/11 | Xinjiang | Northwest | Cross-sectional | Entertainment venues | 4 | Chinese | 81 | 2 | 2.47% |  |  |  |  |  |  |  |  |  |  |
| Lu H, 2003[[32](#_ENREF_32)] | 2002 | Xinjiang | Northwest | Cross-sectional | Detaining education center | 2 | Chinese | 346 | 2 | 0.58% |  |  |  |  |  |  |  |  |  |  |
| Xi SH, 2004[[33](#_ENREF_33)] | 2002/05-2002/07 | Gansu | Northwest | Cross-sectional | Entertainment venues | 7 | Chinese | 88 | 2 | 2.30% |  |  |  |  |  |  |  |  |  |  |
| Li F, 2006[[35](#_ENREF_35)] | 2004/04-2004/06 | Xinjiang | Northwest | Cross-sectional | Entertainment venues | 6 | Chinese | 611 | 10 | 1.64% |  |  |  |  |  |  |  |  |  |  |
| Song Y, 2006[[37](#_ENREF_37)] | 2006 | Xinjiang | Northwest | Cross-sectional | Entertainment venues | 4 | Chinese | 103 | 3 | 2.91% |  |  |  |  |  |  |  |  |  |  |
| Yang SP, 2008a[[38](#_ENREF_38)] | 2007/08-2007/09 | Xinjiang | Northwest | Cross-sectional | Entertainment venues | 3 | Chinese | 251 | 4 | 1.59% |  |  |  |  |  |  |  |  |  |  |
| Zhang CJ, 2001[[112](#_ENREF_112)] | 2000/03-2000/08 | Henan | South Central | Cross-sectional | Detaining education center | 3 | Chinese | 65 | 7 | 10.77% |  |  |  |  |  |  |  |  |  |  |
| Sun L, 2003[[112](#_ENREF_112)] | 2000/06 | Henan | South Central |  | Entertainment venues | 3 | Chinese | 35 | 10 | 28.57% |  |  |  |  |  |  |  |  |  |  |
| Sun J, 2003[[113](#_ENREF_113)] | 2001/09-2001/12 | Henan | South Central | Cross-sectional | Entertainment venues | 2 | Chinese | 220 | 19 | 8.64% |  |  |  |  |  |  |  |  |  |  |
| Liang BH, 2008[[115](#_ENREF_115)] | 2002-2005 | Guangdong | South Central | Cross-sectional | Detaining education center | 5 | Chinese | 1,508 | 134 | 8.89% |  |  |  |  |  |  |  |  |  |  |
| Fan XJ, 2007a[[116](#_ENREF_116)] | 2003 | Hunan | South Central | Cross-sectional | Entertainment venues | 5 | Chinese | 106 | 9 | 8.50% |  |  |  |  |  |  |  |  |  |  |
| Luo J, 2005c[[46](#_ENREF_46)] | 2003/06 | Guangxi | South Central | Cross-sectional | Entertainment venues | 4 | Chinese | 126 | 0 | 0.00% |  |  |  |  |  |  |  |  |  |  |
| Luo J, 2005a[[46](#_ENREF_46)] | 2003/06 | Guangxi | South Central | Cross-sectional | Entertainment venues | 4 | Chinese | 278 | 5 | 1.79% |  |  |  |  |  |  |  |  |  |  |
| Luo J, 2005e[[46](#_ENREF_46)] | 2003/06 | Guangxi | South Central | Cross-sectional | Entertainment venues | 4 | Chinese | 152 | 5 | 3.29% |  |  |  |  |  |  |  |  |  |  |
| Ren Y, 2007[[118](#_ENREF_118)] | 2004 | Guangdong | South Central | National sentinel site | Detaining education center | 6 | Chinese | 518 | 1 | 0.19% |  |  |  |  |  |  |  |  |  |  |
| Xiong CB, 2006a[[49](#_ENREF_49)] | 2004 | Hunan | South Central | Cross-sectional | Entertainment venues | 4 | Chinese | 136 | 2 | 1.50% |  |  |  |  |  |  |  |  |  |  |
| Hong Y, 2009[[50](#_ENREF_50)] | 2004 | Guangxi | South Central | Cross-sectional | Entertainment venues | 4 | English | 411 | 7 | 1.70% |  |  |  |  |  |  |  |  |  |  |
| Wang H, 2005[[51](#_ENREF_51)] | 2004/03-2004/05 | Guangxi | South Central | Cross-sectional | Entertainment venues | 4 | English | 410 | 7 | 1.70% |  |  |  |  |  |  |  |  |  |  |
| Zhou YJ, 2007[[52](#_ENREF_52)] | 2004/05 | Guangxi | South Central | Cross-sectional | Entertainment venues | 6 | Chinese | 80 | 0 | 0.00% |  |  |  |  |  |  |  |  |  |  |
| Sun CX, 2009a[[119](#_ENREF_119)] | 2004/07 | Hubei | South Central | Cross-sectional | Entertainment venues | 3 | Chinese | 59 | 12 | 20.34% |  |  |  |  |  |  |  |  |  |  |
| Wu MS, 2006[[56](#_ENREF_56)] | 2005/03 | Guangxi | South Central | Cross-sectional | Entertainment venues | 2 | Chinese | 187 | 2 | 1.07% |  |  |  |  |  |  |  |  |  |  |
| Zhang JL, 2008a[[57](#_ENREF_57)] | 2006/01-2007/05 | Guangxi | South Central | Cross-sectional | Entertainment venues | 4 | Chinese | 103 | 16 | 15.53% |  |  |  |  |  |  |  |  |  |  |
| Li CG, 2007a[[58](#_ENREF_58)] | 2006/05 | Guangdong | South Central | Cross-sectional | Entertainment venues | 6 | Chinese | 522 | 0 | 0.00% |  |  |  |  |  |  |  |  |  |  |
| Wu ZZ, 2008[[61](#_ENREF_61)] | 2006/11-2007/01 | Guangdong | South Central | Cross-sectional | Entertainment venues | 1 | Chinese | 213 | 1 | 0.47% |  |  |  |  |  |  |  |  |  |  |
| Li XF, 2008a[[121](#_ENREF_121)] | 2007/01-2007/03 | Hunan | South Central | Cross-sectional | Entertainment venues | 4 | Chinese | 142 | 9 | 3.80% |  |  |  |  |  |  |  |  |  |  |
| Lin MH, 2008[[250](#_ENREF_250)] | 2007/08-2008/05 | Guangxi | South Central | Cross-sectional | Entertainment venues | 5 | Chinese | 236 | 4 | 1.69% |  |  |  |  |  |  |  |  |  |  |
| Zhao YY, 2010[[65](#_ENREF_65)] | 2008/10-2008/12 | Guangdong | South Central | Cross-sectional | Entertainment venues | 4 | Chinese | 622 | 26 | 4.18% |  |  |  |  |  |  |  |  |  |  |
| Xiang SB, 2010b[[66](#_ENREF_66)] | 2009 | Hunan | South Central | Cross-sectional | Entertainment venues | 4 | Chinese | 401 | 7 | 1.75% |  |  |  |  |  |  |  |  |  |  |
| Liang HX, 2011[[122](#_ENREF_122)] | 2009-2010 | Hubei | South Central | Cross-sectional | Others | 4 | Chinese | 800 | 64 | 8.00% |  |  |  |  |  |  |  |  |  |  |
| Wang WP, 2008[[123](#_ENREF_123)] | N/A | Hunan | South Central | Cross-sectional | Entertainment venues | 3 | Chinese | 625 | 21 | 3.36% |  |  |  |  |  |  |  |  |  |  |
| Chen SH, 2005[[124](#_ENREF_124)] | N/A | Guangdong | South Central | Cross-sectional | Entertainment venues | 3 | Chinese | 271 | 5 | 1.80% |  |  |  |  |  |  |  |  |  |  |
| Yang P, 2006[[72](#_ENREF_72)] |  | Sichuan | Southwest | Cross-sectional | Entertainment venues | 2 | Chinese | 407 | 33 | 8.10% |  |  |  |  |  |  |  |  |  |  |
| Wu Y, 2002a[[73](#_ENREF_73)] | 2001/09 | Chongqing | Southwest | Cohort | Entertainment venues | 6 | Chinese | 184 | 29 | 15.80% |  |  |  |  |  |  |  |  |  |  |
| Wu Y, 2002b[[73](#_ENREF_73)] | 2001/09 | Chongqing | Southwest | Cohort | Detaining education center | 6 | Chinese | 195 | 36 | 18.50% |  |  |  |  |  |  |  |  |  |  |
| Yang HW, 2003a[[284](#_ENREF_284)] | 2002/04-2002/05 | Sichuan | Southwest | Cross-sectional | Entertainment venues | 2 | Chinese | 126 | 5 | 6.17% |  |  |  |  |  |  |  |  |  |  |
| Wang QQ, 2005[[74](#_ENREF_74)] | 2004/02 | Sichuan | Southwest | Cross-sectional | Entertainment venues | 3 | Chinese | 206 | 19 | 9.20% |  |  |  |  |  |  |  |  |  |  |
| Wu Y, 2007a[[75](#_ENREF_75)] | 2004/09 | Chongqing | Southwest | Cohort | Entertainment venues | 4 | Chinese | 157 | 5 | 3.20% |  |  |  |  |  |  |  |  |  |  |
| Wu Y, 2007b[[75](#_ENREF_75)] | 2004/09 | Chongqing | Southwest | Cohort | Entertainment venues | 4 | Chinese | 169 | 0 | 0.00% |  |  |  |  |  |  |  |  |  |  |
| Cao XY, 2006[[128](#_ENREF_128)] | 2004/12-2005/01 | Sichuan | Southwest | Cross-sectional | Entertainment venues | 5 | Chinese | 203 | 4 | 2.00% |  |  |  |  |  |  |  |  |  |  |
| Ruan Y, 2006[[127](#_ENREF_127)] | 2004/12-2005/01 | Sichuan | Southwest | Cross-sectional | Entertainment venues | 5 | English | 343 | 5 | 1.46% |  |  |  |  |  |  |  |  |  |  |
| Che ZM, 2008[[286](#_ENREF_286)] | 2005/03 - 2006/08 | Yunnan | Southwest |  | Entertainment venues | 0 | Chinese | 12,363 | 60 | 0.49% |  |  |  |  |  |  |  |  |  |  |
| Dai XQ, 2007[[76](#_ENREF_76)] | 2005/06-2005/09 | Chongqing | Southwest | Cross-sectional | Entertainment venues | 5 | Chinese | 341 | 41 | 13.60% |  |  |  |  |  |  |  |  |  |  |
| Tan XJ, 2007[[77](#_ENREF_77)] | 2005/08-2005/09 | Chongqing | Southwest | Cross-sectional | Entertainment venues | 5 | Chinese | 266 | 4 | 1.50% |  |  |  |  |  |  |  |  |  |  |
| Ding XB, 2006[[78](#_ENREF_78)] | 2005/08-2005/09 | Chongqing | Southwest | Cross-sectional | Entertainment venues | 4 | Chinese | 519 | 2 | 0.40% |  |  |  |  |  |  |  |  |  |  |
| Yang X, 2009a[[129](#_ENREF_129)] | 2007 | Yunnan | Southwest | Cross-sectional | Entertainment venues | 1 | Chinese | 371 | 15 | 4.00% |  |  |  |  |  |  |  |  |  |  |
| Gao LL, 2008a[[90](#_ENREF_90)] | 2007 | Yunnan | Southwest | Cross-sectional | Detaining education center | 4 | Chinese | 270 | 1 | 0.37% |  |  |  |  |  |  |  |  |  |  |
| Yang X, 2009f[[129](#_ENREF_129)] | 2007 | Yunnan | Southwest | Cross-sectional | Entertainment venues | 1 | Chinese | 211 | 7 | 3.30% |  |  |  |  |  |  |  |  |  |  |
| Yang X, 2009k[[129](#_ENREF_129)] | 2007 | Yunnan | Southwest | Cross-sectional | Entertainment venues | 1 | Chinese | 109 | 3 | 2.80% |  |  |  |  |  |  |  |  |  |  |
| Yang X, 2009p[[129](#_ENREF_129)] | 2007 | Yunnan | Southwest | Cross-sectional | Entertainment venues | 1 | Chinese | 51 | 5 | 9.80% |  |  |  |  |  |  |  |  |  |  |
| Yan WZ, 2011[[94](#_ENREF_94)] | 2009 | Yunnan | Southwest | Cross-sectional | Entertainment venues | 3 | Chinese | 180 | 1 | 0.56% |  |  |  |  |  |  |  |  |  |  |
| Shao ZP, 2012[[96](#_ENREF_96)] | 2011/05-2011/06 | Chongqing | Southwest | Cross-sectional | Entertainment venues | 4 | Chinese | 318 | 5 | 1.57% |  |  |  |  |  |  |  |  |  |  |
| f) HPV |  |  |  |  |  |  |  |  |  |  |  |  |  |  |  |  |  |  |  |  |
| Wang X, 2013 (b)[[287](#_ENREF_287)] | 2008/04-2009/10 | Zhejiang | East | Cross-sectional | Others | 2 | English | 288 | 188 | 66.70% |  |  |  |  |  |  |  |  |  |  |
| Wang X, 2013[[287](#_ENREF_287)] | 2008/04-2009/10 | Zhejiang | East | Cross-sectional | National sentinel site | 6 | English | 288 | 27 | 9.40% |  |  |  |  |  |  |  |  |  |  |
| Wang X, 2013[[287](#_ENREF_287)] | 2008/04-2009/10 | Zhejiang | East | Cross-sectional | National sentinel site | 6 | English | 288 | 33 | 11.50% |  |  |  |  |  |  |  |  |  |  |
| Wang X, 2013[[287](#_ENREF_287)] | 2008/04-2009/10 | Zhejiang | East | Cross-sectional | National sentinel site | 6 | English | 288 | 83 | 28.80% |  |  |  |  |  |  |  |  |  |  |
| Wang X, 2013[[287](#_ENREF_287)] | 2008/04-2009/10 | Zhejiang | East | Cross-sectional | National sentinel site | 6 | English | 288 | 24 | 8.30% |  |  |  |  |  |  |  |  |  |  |
| Wang X, 2013[[287](#_ENREF_287)] | 2008/04-2009/10 | Zhejiang | East | Cross-sectional | National sentinel site | 6 | English | 288 | 48 | 16.70% |  |  |  |  |  |  |  |  |  |  |
| Wang X, 2013[[287](#_ENREF_287)] | 2008/04-2009/10 | Zhejiang | East | Cross-sectional | National sentinel site | 6 | English | 288 | 43 | 14.90% |  |  |  |  |  |  |  |  |  |  |
| Wang X, 2013[[287](#_ENREF_287)] | 2008/04-2009/10 | Zhejiang | East | Cross-sectional | National sentinel site | 6 | English | 288 | 33 | 11.50% |  |  |  |  |  |  |  |  |  |  |
| Wang X, 2013[[287](#_ENREF_287)] | 2008/04-2009/10 | Zhejiang | East | Cross-sectional | National sentinel site | 6 | English | 288 | 3 | 1.00% |  |  |  |  |  |  |  |  |  |  |
| Wang X, 2013[[287](#_ENREF_287)] | 2008/04-2009/10 | Zhejiang | East | Cross-sectional | National sentinel site | 6 | English | 288 | 9 | 3.10% |  |  |  |  |  |  |  |  |  |  |
| Wang X, 2013[[287](#_ENREF_287)] | 2008/04-2009/10 | Zhejiang | East | Cross-sectional | National sentinel site | 6 | English | 288 | 12 | 4.20% |  |  |  |  |  |  |  |  |  |  |
| Wang X, 2013[[287](#_ENREF_287)] | 2008/04-2009/10 | Zhejiang | East | Cross-sectional | National sentinel site | 6 | English | 288 | 60 | 20.80% |  |  |  |  |  |  |  |  |  |  |
| Wang X, 2013[[287](#_ENREF_287)] | 2008/04-2009/10 | Zhejiang | East | Cross-sectional | National sentinel site | 6 | English | 288 | 42 | 14.60% |  |  |  |  |  |  |  |  |  |  |
| Wang X, 2013[[287](#_ENREF_287)] | 2008/04-2009/10 | Zhejiang | East | Cross-sectional | National sentinel site | 6 | English | 288 | 69 | 24.00% |  |  |  |  |  |  |  |  |  |  |
| Wang X, 2013[[287](#_ENREF_287)] | 2008/04-2009/10 | Zhejiang | East | Cross-sectional | National sentinel site | 6 | English | 288 | 8 | 2.80% |  |  |  |  |  |  |  |  |  |  |
| Wang X, 2013[[287](#_ENREF_287)] | 2008/04-2009/10 | Zhejiang | East | Cross-sectional | National sentinel site | 6 | English | 288 | 18 | 6.20% |  |  |  |  |  |  |  |  |  |  |
| YE Qiu-ru, 2009[[288](#_ENREF_288)] | 2009/01-2009/03 | Liaoning | Northeast | Cross-sectional | Entertainment venues | 6 | Chinese | 45 | 17 | 37.80% |  |  |  |  |  |  |  |  |  |  |
| YE Qiu-ru, 2009[[288](#_ENREF_288)] | 2009/01-2009/03 | Liaoning | Northeast | Cross-sectional | Entertainment venues | 6 | Chinese | 45 | 6 | 12.30% |  |  |  |  |  |  |  |  |  |  |
| YE Qiu-ru, 2009[[288](#_ENREF_288)] | 2009/01-2009/03 | Liaoning | Northeast | Cross-sectional | Entertainment venues | 6 | Chinese | 45 | 5 | 11.10% |  |  |  |  |  |  |  |  |  |  |
| YE Qiu-ru, 2009[[288](#_ENREF_288)] | 2009/01-2009/03 | Liaoning | Northeast | Cross-sectional | Entertainment venues | 4 | Chinese | 45 | 3 | 6.70% |  |  |  |  |  |  |  |  |  |  |
| YE Qiu-ru, 2009[[288](#_ENREF_288)] | 2009/01-2009/03 | Liaoning | Northeast | Cross-sectional | Entertainment venues | 4 | Chinese | 45 | 2 | 4.40% |  |  |  |  |  |  |  |  |  |  |
| YE Qiu-ru, 2009[[288](#_ENREF_288)] | 2009/01-2009/03 | Liaoning | Northeast | Cross-sectional | Entertainment venues | 2 | Chinese | 45 | 2 | 4.40% |  |  |  |  |  |  |  |  |  |  |
| YE Qiu-ru, 2009[[288](#_ENREF_288)] | 2009/01-2009/03 | Liaoning | Northeast | Cross-sectional | Entertainment venues | 2 | Chinese | 45 | 1 | 2.20% |  |  |  |  |  |  |  |  |  |  |
| YE Qiu-ru, 2009[[288](#_ENREF_288)] | 2009/01-2009/03 | Liaoning | Northeast | Cross-sectional | Entertainment venues | 6 | Chinese | 45 | 1 | 2.20% |  |  |  |  |  |  |  |  |  |  |
| YE Qiu-ru, 2009[[288](#_ENREF_288)] | 2009/01-2009/03 | Liaoning | Northeast | Cross-sectional | Entertainment venues | 4 | Chinese | 45 | 1 | 2.20% |  |  |  |  |  |  |  |  |  |  |
| Zhu BY, 2012[[62](#_ENREF_62)] | 2007/05-2007/10 | Guangxi | South Central | Cross-sectional | Entertainment venues | 4 | English | 488 | 11 | 2.25 |  |  |  |  |  |  |  |  |  |  |
| Li HM, 2012 (a)[[289](#_ENREF_289)] | 2009/07-2009/09 | Guangxi | South Central | Cross-sectional | Entertainment venues | 2 | English | 810 | 14 | 1.73% |  |  |  |  |  |  |  |  |  |  |
| Li HM, 2012 (a)[[289](#_ENREF_289)] | 2009/07-2009/09 | Guangxi | South Central | Cross-sectional | Entertainment venues | 6 | English | 810 | 16 | 1.98% |  |  |  |  |  |  |  |  |  |  |
| Li HM, 2012 (a)[[289](#_ENREF_289)] | 2009/07-2009/09 | Guangxi | South Central | Cross-sectional | Entertainment venues | 6 | English | 810 | 53 | 6.54% |  |  |  |  |  |  |  |  |  |  |
| Li HM, 2012 (a)[[289](#_ENREF_289)] | 2009/07-2009/09 | Guangxi | South Central | Cross-sectional | Entertainment venues | 6 | English | 810 | 23 | 2.84% |  |  |  |  |  |  |  |  |  |  |
| Li HM, 2012 (a)[[289](#_ENREF_289)] | 2009/07-2009/09 | Guangxi | South Central | Cross-sectional | Entertainment venues | 6 | English | 810 | 11 | 1.36% |  |  |  |  |  |  |  |  |  |  |
| g) HSV |  |  |  |  |  |  |  |  |  |  |  |  |  |  |  |  |  |  |  |  |
| Yang P, 2005a[[7](#_ENREF_7)] | 2002/12 | Fujian | East | Cross-sectional | Entertainment venues | 3 | Chinese | 149 | 1 | 0.67% |  |  |  |  |  |  |  |  |  |  |
| Wang HY, 2005[[143](#_ENREF_143)] | 2004/03-2004/12 | Shandong | East | Cross-sectional | Others | 4 | Chinese | 456 | 148 | 32.46% |  |  |  |  |  |  |  |  |  |  |
| Wang YF, 2008[[290](#_ENREF_290)] | 2005-2006 | Shandong | East | Cross-sectional | Entertainment venues | 4 | Chinese | 132 | 38 | 28.80% |  |  |  |  |  |  |  |  |  |  |
| Yang JY, 2011[[21](#_ENREF_21)] | 2007/10-2007/11 | Jiangsu | East | Cross-sectional | Entertainment venues | 4 | Chinese | 247 | 3 | 1.22% |  |  |  |  |  |  |  |  |  |  |
| Yang Y, 2011a[[291](#_ENREF_291)] | 2008/06-2008/10 | Shanghai | East | Cross-sectional | Entertainment venues | 5 | English | 411 | 187 | 45.50% |  |  |  |  |  |  |  |  |  |  |
| Yang Y, 2011b[[291](#_ENREF_291)] | 2009/05-2009/08 | Shanghai | East | Cross-sectional | Entertainment venues | 2 | English | 411 | 206 | 50.10% |  |  |  |  |  |  |  |  |  |  |
| Tang ZL, 2011[[28](#_ENREF_28)] | 2010/03-2010/07 | Shandong | East | Cross-sectional | Entertainment venues | 6 | Chinese | 106 | 15 | 14.20% |  |  |  |  |  |  |  |  |  |  |
| Tang ZL, 2011[[28](#_ENREF_28)] | 2010/03-2010/07 | Shandong | East | Cross-sectional | Entertainment venues | 6 | Chinese | 216 | 16 | 7.40% |  |  |  |  |  |  |  |  |  |  |
| Li Y, 2011[[31](#_ENREF_31)] | 2010 | Heilongjiang | Northeast | Cross-sectional | National sentinel site | 4 | Chinese | 7,662 | 168 | 2.20% |  |  |  |  |  |  |  |  |  |  |
| Yang SP, 2008a[[38](#_ENREF_38)] | 2007/08-2007/09 | Xinjiang | Northwest | Cross-sectional | Entertainment venues | 2 | Chinese | 251 | 0 | 0.00% |  |  |  |  |  |  |  |  |  |  |
| Luo J, 2005c[[46](#_ENREF_46)] | 2003/06 | Guangxi | South Central | Cross-sectional | Entertainment venues | 4 | Chinese | 126 | 1 | 0.79% |  |  |  |  |  |  |  |  |  |  |
| Luo J, 2005a[[46](#_ENREF_46)] | 2003/06 | Guangxi | South Central | Cross-sectional | Entertainment venues | 4 | Chinese | 278 | 4 | 1.43% |  |  |  |  |  |  |  |  |  |  |
| Luo J, 2005e[[46](#_ENREF_46)] | 2003/06 | Guangxi | South Central | Cross-sectional | Entertainment venues | 4 | Chinese | 152 | 3 | 1.97% |  |  |  |  |  |  |  |  |  |  |
| Ren Y, 2007[[118](#_ENREF_118)] | 2004 | Guangdong | South Central | National sentinel site | Detaining education center | 6 | Chinese | 518 | 24 | 4.63% |  |  |  |  |  |  |  |  |  |  |
| Zhou YJ, 2007[[52](#_ENREF_52)] | 2004/05 | Guangxi | South Central | Cross-sectional | Entertainment venues | 6 | Chinese | 80 | 0 | 0.00% |  |  |  |  |  |  |  |  |  |  |
| Sun CX, 2009a[[119](#_ENREF_119)] | 2004/07 | Hubei | South Central | Cross-sectional | Entertainment venues | 3 | Chinese | 59 | 1 | 1.69% |  |  |  |  |  |  |  |  |  |  |
| Zhang WS, 2006a[[55](#_ENREF_55)] | 2004/10-2005/04 | Hubei | South Central | Cross-sectional | Entertainment venues | 4 | Chinese | 68 | 18 | 26.47% |  |  |  |  |  |  |  |  |  |  |
| Zhang WS, 2006b[[55](#_ENREF_55)] | 2004/10-2005/04 | Hubei | South Central | Cross-sectional | Entertainment venues | 4 | Chinese | 68 | 8 | 11.76% |  |  |  |  |  |  |  |  |  |  |
| Wu MS, 2006[[56](#_ENREF_56)] | 2005/03 | Guangxi | South Central | Cross-sectional | Entertainment venues | 2 | Chinese | 187 | 2 | 1.07% |  |  |  |  |  |  |  |  |  |  |
| Dai XN, 2009[[292](#_ENREF_292)] | 2006/03-2006/11 | Guangdong | South Central | Cross-sectional | Detaining education center | 5 | Chinese | 780 | 193 | 24.74% |  |  |  |  |  |  |  |  |  |  |
| Zhu BY, 2012[[62](#_ENREF_62)] | 2007/05-2007/10 | Guangxi | South Central | Cross-sectional | Entertainment venues | 6 | English | 488 | 2 | 0.41% |  |  |  |  |  |  |  |  |  |  |
| Xiang SB, 2010b[[66](#_ENREF_66)] | 2009 | Hunan | South Central | Cross-sectional | Entertainment venues | 4 | Chinese | 401 | 7 | 1.75% |  |  |  |  |  |  |  |  |  |  |
| Liang HX, 2011[[122](#_ENREF_122)] | 2009-2010 | Hubei | South Central | Cross-sectional | Others | 4 | Chinese | 800 | 16 | 2.00% |  |  |  |  |  |  |  |  |  |  |
| Wang WP, 2008[[123](#_ENREF_123)] |  | Hunan | South Central | Cross-sectional | Entertainment venues | 3 | Chinese | 625 | 17 | 2.72% |  |  |  |  |  |  |  |  |  |  |
| Chen SH, 2005[[124](#_ENREF_124)] |  | Guangdong | South Central | Cross-sectional | Entertainment venues | 3 | Chinese | 271 | 3 | 1.10% |  |  |  |  |  |  |  |  |  |  |
| Yang P, 2006[[72](#_ENREF_72)] |  | Sichuan | Southwest | Cross-sectional | Entertainment venues | 2 | Chinese | 407 | 4 | 1.00% |  |  |  |  |  |  |  |  |  |  |
| Ngo TD, 2008[[293](#_ENREF_293)] | 2004 | Yunnan | Southwest | Cross-sectional | Detaining education center | 3 | English | 310 | 140 | 45.16% |  |  |  |  |  |  |  |  |  |  |
| Wang QQ, 2005[[74](#_ENREF_74)] | 2004/02 | Sichuan | Southwest | Cross-sectional | Entertainment venues | 3 | Chinese | 206 | 2 | 1.00% |  |  |  |  |  |  |  |  |  |  |
| Li YY, 2009a[[126](#_ENREF_126)] | 2004/10-2006/12 | Yunnan | Southwest | Cross-sectional | Entertainment venues | 4 | Chinese | 100 | 0 | 0.00% |  |  |  |  |  |  |  |  |  |  |
| Wang HB, 2010a[[79](#_ENREF_79)] | 2006/03 | Yunnan | Southwest | Cross-sectional | Entertainment venues | 7 | English | 737 | 502 | 68.10% |  |  |  |  |  |  |  |  |  |  |
| Xu J, 2011[[80](#_ENREF_80)] | 2006/03 - 2007/04 | Yunnan | Southwest | Cohort | Others | 3 | English | 1,642 | 1099 | 66.93% |  |  |  |  |  |  |  |  |  |  |
| Wang H, 2012[[81](#_ENREF_81)] | 2006/03 - 2009/11 | Yunnan | Southwest | Cohort | Entertainment venues | 6 | English | 851 | 532 | 62.50% |  |  |  |  |  |  |  |  |  |  |
| Li QH, 2010a[[82](#_ENREF_82)] | 2006/03-2006/04 | Yunnan | Southwest | Cross-sectional | Entertainment venues | 6 | Chinese | 716 | 489 | 68.29% |  |  |  |  |  |  |  |  |  |  |
| Xu JJ, 2008[[83](#_ENREF_83)] | 2006/03-2006/04 | Yunnan | Southwest | Cross-sectional | Entertainment venues | 6 | English | 96 | 68 | 70.83% |  |  |  |  |  |  |  |  |  |  |
| Wang H, 2009[[84](#_ENREF_84)] | 2006/03-2006/05 | Yunnan | Southwest | Cross-sectional | Entertainment venues | 4 | English | 737 | 502 | 68.11% |  |  |  |  |  |  |  |  |  |  |
| Wang H, 2009e[[84](#_ENREF_84)] | 2006/03-2006/05 | Yunnan | Southwest | Cross-sectional | Entertainment venues | 4 | English | 458 | 294 | 64.19% |  |  |  |  |  |  |  |  |  |  |
| Wang H, 2009f[[84](#_ENREF_84)] | 2006/03-2006/05 | Yunnan | Southwest | Cross-sectional | Entertainment venues | 4 | English | 279 | 208 | 74.50% |  |  |  |  |  |  |  |  |  |  |
| Wang HB, 2010a[[79](#_ENREF_79)] | 2006/03-2006/12 | Yunnan | Southwest | Cross-sectional | Entertainment venues | 6 | English | 705 | 502 | 71.21% |  |  |  |  |  |  |  |  |  |  |
| Wang H, 2011[[86](#_ENREF_86)] | 2006/03-2009/11 | Yunnan | Southwest | Cohort | Entertainment venues | 4 | English | 1,866 | 779 | 41.74% |  |  |  |  |  |  |  |  |  |  |
| Li QH, 2009a[[88](#_ENREF_88)] | 2006/09-2006/10 | Yunnan | Southwest | Cross-sectional | Entertainment venues | 7 | Chinese | 335 | 201 | 60.00% |  |  |  |  |  |  |  |  |  |  |
| Li QH, 2009b[[88](#_ENREF_88)] | 2006/09-2006/10 | Yunnan | Southwest | Cross-sectional | Entertainment venues | 7 | Chinese | 312 | 225 | 72.12% |  |  |  |  |  |  |  |  |  |  |
| Li QH, 2009c[[88](#_ENREF_88)] | 2006/09-2006/10 | Yunnan | Southwest | Cross-sectional | Entertainment venues | 7 | Chinese | 87 | 76 | 87.36% |  |  |  |  |  |  |  |  |  |  |
| Wang GX, 2008a[[89](#_ENREF_89)] | 2006/09-2006/10 | Yunnan | Southwest | Cross-sectional | Entertainment venues | 5 | Chinese | 747 | 512 | 68.54% |  |  |  |  |  |  |  |  |  |  |
| Wang GX, 2008b[[89](#_ENREF_89)] | 2006/09-2006/10 | Yunnan | Southwest | Cross-sectional | Entertainment venues | 5 | Chinese | 266 | 169 | 63.53% |  |  |  |  |  |  |  |  |  |  |
| Wang GX, 2008c[[89](#_ENREF_89)] | 2006/09-2006/10 | Yunnan | Southwest | Cross-sectional | Entertainment venues | 5 | Chinese | 351 | 239 | 68.09% |  |  |  |  |  |  |  |  |  |  |
| Wang GX, 2008d[[89](#_ENREF_89)] | 2006/09-2006/10 | Yunnan | Southwest | Cross-sectional | Entertainment venues | 5 | Chinese | 120 | 96 | 80.00% |  |  |  |  |  |  |  |  |  |  |
| Wang HB, 2010b[[79](#_ENREF_79)] | 2006/10 | Yunnan | Southwest | Cross-sectional | Entertainment venues | 2 | English | 747 | 513 | 68.70% |  |  |  |  |  |  |  |  |  |  |
| Yang X, 2009a[[129](#_ENREF_129)] | 2007 | Yunnan | Southwest | Cross-sectional | Entertainment venues | 6 | Chinese | 371 | 10 | 2.70% |  |  |  |  |  |  |  |  |  |  |
| Gao LL, 2008a[[90](#_ENREF_90)] | 2007 | Yunnan | Southwest | Cross-sectional | Detaining education center | 4 | Chinese | 270 | 0 | 0.00% |  |  |  |  |  |  |  |  |  |  |
| Yang X, 2009f[[129](#_ENREF_129)] | 2007 | Yunnan | Southwest | Cross-sectional | Entertainment venues | 4 | Chinese | 211 | 5 | 2.40% |  |  |  |  |  |  |  |  |  |  |
| Yang X, 2009k[[129](#_ENREF_129)] | 2007 | Yunnan | Southwest | Cross-sectional | Entertainment venues | 2 | Chinese | 109 | 4 | 3.70% |  |  |  |  |  |  |  |  |  |  |
| Yang X, 2009p[[129](#_ENREF_129)] | 2007 | Yunnan | Southwest | Cross-sectional | Entertainment venues | 1 | Chinese | 51 | 1 | 2.00% |  |  |  |  |  |  |  |  |  |  |
| Wang HB, 2010c[[79](#_ENREF_79)] | 2007/05 | Yunnan | Southwest | Cross-sectional | Entertainment venues | 2 | English | 705 | 502 | 71.20% |  |  |  |  |  |  |  |  |  |  |
| Wang HB, 2010b[[79](#_ENREF_79)] | 2007/05 | Yunnan | Southwest |  | Others | 6 | English | 705 | 502 | 71.21% |  |  |  |  |  |  |  |  |  |  |
| Yao Y, 2012[[92](#_ENREF_92)] | 2007/09-2007/10 | Yunnan | Southwest | Cross-sectional | Entertainment venues | 4 | English | 399 | 269 | 67.40% |  |  |  |  |  |  |  |  |  |  |
| Zhang XD, 2012[[95](#_ENREF_95)] | 2010/07-2011/02 | Yunnan | Southwest | Cross-sectional | Entertainment venues | 4 | English | 189 | 1 | 0.50% |  |  |  |  |  |  |  |  |  |  |
| Shao ZP, 2012[[96](#_ENREF_96)] | 2011/05-2011/06 | Chongqing | Southwest | Cross-sectional | Entertainment venues | 2 | Chinese | 318 | 2 | 0.63% |  |  |  |  |  |  |  |  |  |  |
| h)HBV |  |  |  |  |  |  |  |  |  |  |  |  |  |  |  |  |  |  |  |  |
| Liu HB, 2012[[294](#_ENREF_294)] |  | Jiangxi | East | Cross-sectional | Others | 2 | Chinese | 200 | 18 | 9.00% |  |  |  |  |  |  |  |  |  |  |
| Zhao LH, 2006[[295](#_ENREF_295)] | 2004/0 -2004/12 | Shandong | East | Cross-sectional | Detaining education center | 4 | Chinese | 361 | 34 | 9.42% |  |  |  |  |  |  |  |  |  |  |
| Zhao LH, 2006[[295](#_ENREF_295)] | 2004/0 -2004/12 | Shandong | East | Cross-sectional | Detaining education center | 4 | Chinese | 361 | 34 | 9.42% |  |  |  |  |  |  |  |  |  |  |
| Wang HY, 2005[[143](#_ENREF_143)] | 2004/03-2004/12 | Shandong | East | Cross-sectional | Others | 4 | Chinese | 456 | 33 | 7.24% |  |  |  |  |  |  |  |  |  |  |
| Luo Y, 2008a[[153](#_ENREF_153)] | 2007/02-2007/07 | Zhejiang | East | Cross-sectional | Entertainment venues | 5 | Chinese | 267 | 15 | 5.62% |  |  |  |  |  |  |  |  |  |  |
| Xue FH, 2009a[[157](#_ENREF_157)] | 2008/04-2008/06 | Zhejiang | East | Cross-sectional | Entertainment venues | 7 | Chinese | 403 | 26 | 6.45% |  |  |  |  |  |  |  |  |  |  |
| Yin FL, 2009a[[296](#_ENREF_296)] | 2008/05-2008/06 | Shanghai | East | Cross-sectional | Entertainment venues | 3 | Chinese | 452 | 32 | 7.10% |  |  |  |  |  |  |  |  |  |  |
| Yin FL, 2009c[[296](#_ENREF_296)] | | | | | | | | | | | 2008/05-2008/06 | Shanghai | East | Cross-sectional | Entertainment venues | 3 | Chinese | 98 | 4 | 4.10% |
| Yin FL, 2009d[[296](#_ENREF_296)] | 2008/05-2008/06 | Shanghai | East | Cross-sectional | Entertainment venues | 3 | Chinese | 85 | 4 | 4.70% |  |  |  |  |  |  |  |  |  |  |
| Yin FL, 2009e[[296](#_ENREF_296)] | 2008/05-2008/06 | Shanghai | East | Cross-sectional | Entertainment venues | 3 | Chinese | 269 | 24 | 8.90% |  |  |  |  |  |  |  |  |  |  |
| Wang JH, 2010[[163](#_ENREF_163)] | 2009/04 | Shandong | East | Cross-sectional | Entertainment venues | 5 | Chinese | 348 | 5 | 1.44% |  |  |  |  |  |  |  |  |  |  |
| Wang JH, 2010b[[163](#_ENREF_163)] | 2009/04 | Shandong | East | Cross-sectional | Entertainment venues | 5 | Chinese | 298 | 5 | 1.68% |  |  |  |  |  |  |  |  |  |  |
| Wang JH, 2010c[[163](#_ENREF_163)] | 2009/04 | Shandong | East | Cross-sectional | Entertainment venues | 5 | Chinese | 50 | 0 | 0.00% |  |  |  |  |  |  |  |  |  |  |
| Chen GS, 2010[[165](#_ENREF_165)] | 2009/04-2009/06 | Jiangxi | East | Cross-sectional | Entertainment venues | 6 | Chinese | 401 | 63 | 15.71% |  |  |  |  |  |  |  |  |  |  |
| Yu QL, 2007[[212](#_ENREF_212)] |  | Gansu | Northwest | Cross-sectional | Others | 4 | Chinese | 298 | 32 | 10.80% |  |  |  |  |  |  |  |  |  |  |
| Fu ZW, 2012[[297](#_ENREF_297)] | 2009 | Hainan | South Central | Cross-sectional | Entertainment venues | 4 | Chinese | 200 | 19 | 9.50% |  |  |  |  |  |  |  |  |  |  |
| Fu ZW, 2012[[297](#_ENREF_297)] | 2009 | Hainan | South Central | Cross-sectional | Entertainment venues | 2 | Chinese | 200 | 19 | 9.50% |  |  |  |  |  |  |  |  |  |  |
| Qiu YQ, 2004[[298](#_ENREF_298)] |  | Guangdong | South Central | Cross-sectional | Others | 5 | Chinese | 189 | 120 | 63.49% |  |  |  |  |  |  |  |  |  |  |
| Zheng K, 2003[[234](#_ENREF_234)] | 2002/06-2008/12 | Hainan | South Central | National Sentinel Site | Detaining education center | 3 | Chinese | 1,144 | 180 | 15.73% |  |  |  |  |  |  |  |  |  |  |
| Guo P, 2005[[299](#_ENREF_299)] | 2003/10 - 2003/11 | Guangdong | South Central | Cross-sectional | Detaining education center | 3 | Chinese | 633 | 95 | 15.00% |  |  |  |  |  |  |  |  |  |  |
| Yan LM, 2007[[300](#_ENREF_300)] | 2005/03-2005/07 | Hubei | South Central | Cross-sectional | Entertainment venues | 2 | Chinese | 115 | 9 | 7.83% |  |  |  |  |  |  |  |  |  |  |
| Jiang N, 2012[[261](#_ENREF_261)] | 2009-2010 | Henan | South Central | Cross-sectional | Others | 3 | Chinese | 475 | 38 | 14.95% |  |  |  |  |  |  |  |  |  |  |
| Chen RC, 2004[[301](#_ENREF_301)] |  | Guangdong | South Central | Cross-sectional | Entertainment venues | 4 | Chinese | 52 | 10 | 19.20% |  |  |  |  |  |  |  |  |  |  |
| Lei ZQ, 2005[[273](#_ENREF_273)] | 2004/04 | Sichuan | Southwest | Cross-sectional | Entertainment venues | 4 | Chinese | 259 | 29 | 11.20% |  |  |  |  |  |  |  |  |  |  |
| i) HCV |  |  |  |  |  |  |  |  |  |  |  |  |  |  |  |  |  |  |  |  |
| Liao CQ, 2011[[97](#_ENREF_97)] |  | Shanghai | East | Cross-sectional | Entertainment venues | 3 | Chinese | 401 | 1 | 0.20% |  |  |  |  |  |  |  |  |  |  |
| Liu HB, 2012[[294](#_ENREF_294)] |  | Jiangxi | East | Cross-sectional | Others | 2 | Chinese | 200 | 26 | 13.00% |  |  |  |  |  |  |  |  |  |  |
| Qin QR, 2011[[134](#_ENREF_134)] |  | Anhui | East | Cross-sectional | Entertainment venues | 4 | Chinese | 182 | 1 | 0.55% |  |  |  |  |  |  |  |  |  |  |
| Zhang H, 2011[[133](#_ENREF_133)] |  | Fujian | East | Cross-sectional | Entertainment venues | 4 | Chinese | 479 | 3 | 0.63% |  |  |  |  |  |  |  |  |  |  |
| Zhang Q, 2008a[[302](#_ENREF_302)] |  | Anhui | East | National Sentinel Site | Entertainment venues | 3 | Chinese | 394 | 6 | 1.52% |  |  |  |  |  |  |  |  |  |  |
| Zhao LH, 2006[[295](#_ENREF_295)] | 2004/0 -2004/12 | Shandong | East | Cross-sectional | Detaining education center | 4 | Chinese | 361 | 13 | 3.60% |  |  |  |  |  |  |  |  |  |  |
| Wang HY, 2005[[143](#_ENREF_143)] | 2004/03-2004/12 | Shandong | East | Cross-sectional | Others | 4 | Chinese | 456 | 4 | 0.88% |  |  |  |  |  |  |  |  |  |  |
| Zheng H, 2012[[144](#_ENREF_144)] | 2004/07-2004/08 | Jiangsu | East | Cross-sectional | Entertainment venues | 3 | Chinese | 105 | 0 | 0.00% |  |  |  |  |  |  |  |  |  |  |
| Yu WX, 2006[[303](#_ENREF_303)] | 2005/07-2005/09 | Jiangsu | East | Cross-sectional | Entertainment venues | 4 | Chinese | 258 | 8 | 3.10% |  |  |  |  |  |  |  |  |  |  |
| Jin TL, 2005a[[304](#_ENREF_304)] | 2004/05 | Zhejiang | East | Cross-sectional | Entertainment venues | 5 | Chinese | 137 | 4 | 2.92% |  |  |  |  |  |  |  |  |  |  |
| Luo Y, 2008a[[153](#_ENREF_153)] | 2007/02-2007/07 | Zhejiang | East | Cross-sectional | Entertainment venues | 5 | Chinese | 267 | 2 | 0.80% |  |  |  |  |  |  |  |  |  |  |
| Jin YL, 2009a[[305](#_ENREF_305)] | 2008/05-2008/06 | Anhui | East | Cross-sectional | Entertainment venues | 4 | Chinese | 500 | 5 | 1.00% |  |  |  |  |  |  |  |  |  |  |
| Yin FL, 2009a[[296](#_ENREF_296)] | 2008/05-2008/06 | Shanghai | East | Cross-sectional | Entertainment venues | 3 | Chinese | 452 | 5 | 1.10% |  |  |  |  |  |  |  |  |  |  |
| Yin FL, 2009c[[296](#_ENREF_296)] | 2008/05-2008/06 | Shanghai | East | Cross-sectional | Entertainment venues | 3 | Chinese | 98 | 0 | 0.00% |  |  |  |  |  |  |  |  |  |  |
| Yin FL, 2009d[[296](#_ENREF_296)] | 2008/05-2008/06 | Shanghai | East | Cross-sectional | Entertainment venues | 3 | Chinese | 85 | 1 | 1.20% |  |  |  |  |  |  |  |  |  |  |
| Yin FL, 2009e[[296](#_ENREF_296)] | 2008/05-2008/06 | Shanghai | East | Cross-sectional | Entertainment venues | 3 | Chinese | 269 | 4 | 1.50% |  |  |  |  |  |  |  |  |  |  |
| Wang J, 2008a[[103](#_ENREF_103)] | 2008/06 | Jiangsu | East | Cross-sectional | Entertainment venues | 4 | Chinese | 400 | 26 | 6.50% |  |  |  |  |  |  |  |  |  |  |
| Wang J, 2008b[[103](#_ENREF_103)] | 2008/06 | Jiangsu | East | Cross-sectional | Entertainment venues | 4 | Chinese | 39 | 8 | 20.50% |  |  |  |  |  |  |  |  |  |  |
| Wang J, 2008c[[103](#_ENREF_103)] | 2008/06 | Jiangsu | East | Cross-sectional | Entertainment venues | 4 | Chinese | 361 | 18 | 4.99% |  |  |  |  |  |  |  |  |  |  |
| Chen ZH, 2012[[161](#_ENREF_161)] | 2009 | Zhejiang | East | Cross-sectional | Entertainment venues | 3 | Chinese | 409 | 1 | 0.24% |  |  |  |  |  |  |  |  |  |  |
| Liao MZ, 2010a[[306](#_ENREF_306)] | 2009 | Shandong | East | National sentinel site | VCT | 4 | Chinese | 1,561 | 0 | 0.00% |  |  |  |  |  |  |  |  |  |  |
| Wang F, 2010a[[24](#_ENREF_24)] | 2009 | Anhui | East | Cross-sectional | Entertainment venues | 4 | Chinese | 75 | 0 | 0.00% |  |  |  |  |  |  |  |  |  |  |
| Shao MC, 2010[[106](#_ENREF_106)] | 2009/04 | Jiangsu | East | Cross-sectional | Entertainment venues | 1 | Chinese | 402 | 2 | 0.50% |  |  |  |  |  |  |  |  |  |  |
| Wang JH, 2010[[163](#_ENREF_163)] | 2009/04 | Shandong | East | Cross-sectional | Entertainment venues | 5 | Chinese | 348 | 4 | 1.15% |  |  |  |  |  |  |  |  |  |  |
| Wang JH, 2010b[[163](#_ENREF_163)] | 2009/04 | Shandong | East | Cross-sectional | Entertainment venues | 5 | Chinese | 298 | 3 | 1.01% |  |  |  |  |  |  |  |  |  |  |
| Wang JH, 2010c[[163](#_ENREF_163)] | 2009/04 | Shandong | East | Cross-sectional | Entertainment venues | 5 | Chinese | 50 | 1 | 2.00% |  |  |  |  |  |  |  |  |  |  |
| Chen GS, 2010[[165](#_ENREF_165)] | 2009/04-2009/06 | Jiangxi | East | Cross-sectional | Entertainment venues | 6 | Chinese | 401 | 1 | 0.25% |  |  |  |  |  |  |  |  |  |  |
| Qian ZH, 2012[[172](#_ENREF_172)] | 2009-2010 | Jiangsu | East | Cross-sectional | Detaining education center | 4 | Chinese | 578 | 13 | 2.25% |  |  |  |  |  |  |  |  |  |  |
| Chen ZH, 2012[[161](#_ENREF_161)] | 2009-2011 | Zhejiang | East | Cross-sectional | Entertainment venues | 3 | Chinese | 1,163 | 6 | 0.52% |  |  |  |  |  |  |  |  |  |  |
| Chen ZH, 2012[[161](#_ENREF_161)] | 2010 | Zhejiang | East | Cross-sectional | Entertainment venues | 3 | Chinese | 400 | 3 | 0.75% |  |  |  |  |  |  |  |  |  |  |
| Miao XL, 2011[[174](#_ENREF_174)] | 2010 | Jiangsu | East | Cross-sectional | Entertainment venues | 4 | Chinese | 800 | 3 | 0.40% |  |  |  |  |  |  |  |  |  |  |
| Sun XQ, 2011[[307](#_ENREF_307)] | 2010 | Anhui | East | Cross-sectional | Entertainment venues | 4 | Chinese | 354 | 4 | 1.10% |  |  |  |  |  |  |  |  |  |  |
| Liu LL, 2011[[179](#_ENREF_179)] | 2010/04-2010/06 | Jiangsu | East | Cross-sectional | Entertainment venues | 6 | Chinese | 407 | 1 | 0.25% |  |  |  |  |  |  |  |  |  |  |
| Jiang J, 2012[[180](#_ENREF_180)] | 2010/07 | Zhejiang | East | Cross-sectional | Detaining education center | 4 | Chinese | 439 | 6 | 1.37% |  |  |  |  |  |  |  |  |  |  |
| Qin CZ, 2012[[183](#_ENREF_183)] | 2010/6-2011/04 | Jiangsu | East | Cross-sectional | Entertainment venues | 4 | Chinese | 817 | 1 | 0.10% |  |  |  |  |  |  |  |  |  |  |
| Qin CZ, 2012[[183](#_ENREF_183)] | 2010/6-2011/04 | Jiangsu | East | Cross-sectional | Entertainment venues | 4 | Chinese | 215 | 1 | 0.50% |  |  |  |  |  |  |  |  |  |  |
| Qin CZ, 2012[[183](#_ENREF_183)] | 2010/6-2011/04 | Jiangsu | East | Cross-sectional | Entertainment venues | 4 | Chinese | 444 | 0 | 0.00% |  |  |  |  |  |  |  |  |  |  |
| Qin CZ, 2012[[183](#_ENREF_183)] | 2010/6-2011/04 | Jiangsu | East | Cross-sectional | Entertainment venues | 4 | Chinese | 158 | 0 | 0.00% |  |  |  |  |  |  |  |  |  |  |
| Chen ZH, 2012[[161](#_ENREF_161)] | 2011 | Zhejiang | East | Cross-sectional | Entertainment venues | 3 | Chinese | 354 | 2 | 0.56% |  |  |  |  |  |  |  |  |  |  |
| Hao XG, 2012[[308](#_ENREF_308)] | 2011 | Zhejiang | East | Cross-sectional | National sentinel site | 4 | Chinese | 400 | 3 | 0.75% |  |  |  |  |  |  |  |  |  |  |
| Hao XG, 2012[[308](#_ENREF_308)] | 2011 | Zhejiang | East | Cross-sectional | National sentinel site | 4 | Chinese | 160 | 2 | 1.30% |  |  |  |  |  |  |  |  |  |  |
| Hao XG, 2012[[308](#_ENREF_308)] | 2011 | Zhejiang | East | Cross-sectional | National sentinel site | 4 | Chinese | 83 | 0 | 0.00% |  |  |  |  |  |  |  |  |  |  |
| Hao XG, 2012[[308](#_ENREF_308)] | 2011 | Zhejiang | East | Cross-sectional | National sentinel site | 4 | Chinese | 157 | 1 | 0.60% |  |  |  |  |  |  |  |  |  |  |
| Wang DL, 2012[[309](#_ENREF_309)] | 2011 | Jiangsu | East | Cross-sectional | National sentinel site | 4 | Chinese | 400 | 6 | 1.50% |  |  |  |  |  |  |  |  |  |  |
| Wang DL, 2012[[309](#_ENREF_309)] | 2011 | Jiangsu | East | Cross-sectional | National sentinel site | 4 | Chinese | 44 | 0 | 0.00% |  |  |  |  |  |  |  |  |  |  |
| Wang DL, 2012[[309](#_ENREF_309)] | 2011 | Jiangsu | East | Cross-sectional | National sentinel site | 4 | Chinese | 258 | 1 | 0.40% |  |  |  |  |  |  |  |  |  |  |
| Wang DL, 2012[[309](#_ENREF_309)] | 2011 | Jiangsu | East | Cross-sectional | National sentinel site | 4 | Chinese | 98 | 5 | 5.10% |  |  |  |  |  |  |  |  |  |  |
| Wang YP, 2012[[310](#_ENREF_310)] | 2011 | Jiangsu | East | Cross-sectional | National sentinel site | 4 | Chinese | 404 | 7 | 1.75% |  |  |  |  |  |  |  |  |  |  |
| Wu J, 2012[[184](#_ENREF_184)] | 2011 | Jiangsu | East | Cross-sectional | Entertainment venues | 4 | Chinese | 405 | 3 | 0.74% |  |  |  |  |  |  |  |  |  |  |
| Yang YH, 2012[[186](#_ENREF_186)] | 2011 | Fujian | East | Cross-sectional | National sentinel site | 4 | Chinese | 868 | 1 | 0.10% |  |  |  |  |  |  |  |  |  |  |
| Pan GL, 2012[[189](#_ENREF_189)] | 2011/04-2011/07 | Zhejiang | East | Cross-sectional | Entertainment venues | 4 | Chinese | 617 | 3 | 0.49% |  |  |  |  |  |  |  |  |  |  |
| Pan GL, 2012[[189](#_ENREF_189)] | 2011/04-2011/07 | Zhejiang | East | Cross-sectional | Entertainment venues | 4 | Chinese | 88 | 0 | 0.00% |  |  |  |  |  |  |  |  |  |  |
| Pan GL, 2012[[189](#_ENREF_189)] | 2011/04-2011/07 | Zhejiang | East | Cross-sectional | Entertainment venues | 4 | Chinese | 255 | 1 | 0.39% |  |  |  |  |  |  |  |  |  |  |
| Pan GL, 2012[[189](#_ENREF_189)] | 2011/04-2011/07 | Zhejiang | East | Cross-sectional | Entertainment venues | 4 | Chinese | 274 | 2 | 0.92% |  |  |  |  |  |  |  |  |  |  |
| Qiu ZH, 2012[[311](#_ENREF_311)] | 2011/04-2011/07 | Zhejiang | East | Cross-sectional | Entertainment venues | 4 | Chinese | 400 | 0 | 0.00% |  |  |  |  |  |  |  |  |  |  |
| Xu JS, 2012[[188](#_ENREF_188)] | 2011/04-2011/07 | Jiangsu | East | Cross-sectional | Others | 4 | Chinese | 10,916 | 68 | 0.62% |  |  |  |  |  |  |  |  |  |  |
| Xu JS, 2012[[188](#_ENREF_188)] | 2011/04-2011/07 | Jiangsu | East | Cross-sectional | Others | 4 | Chinese | 1,591 | 7 | 0.44% |  |  |  |  |  |  |  |  |  |  |
| Xu JS, 2012[[188](#_ENREF_188)] | 2011/04-2011/07 | Jiangsu | East | Cross-sectional | Others | 4 | Chinese | 5,500 | 25 | 0.45% |  |  |  |  |  |  |  |  |  |  |
| Xu JS, 2012[[188](#_ENREF_188)] | 2011/04-2011/07 | Jiangsu | East | Cross-sectional | Others | 4 | Chinese | 3,746 | 36 | 0.96% |  |  |  |  |  |  |  |  |  |  |
| Xu JS, 2012[[188](#_ENREF_188)] | 2011/04-2011/07 | Jiangsu | East | Cross-sectional | Others | 4 | Chinese | 3,746 | 36 | 0.96% |  |  |  |  |  |  |  |  |  |  |
| Ye ZM, 2012[[312](#_ENREF_312)] | 2011/04-2011/07 | Zhejiang | East | Cross-sectional | Entertainment venues | 3 | Chinese | 830 | 4 | 0.48% |  |  |  |  |  |  |  |  |  |  |
| Ye ZM, 2012[[312](#_ENREF_312)] | 2011/04-2011/07 | Zhejiang | East | Cross-sectional | Entertainment venues | 3 | Chinese | 114 | 1 | 0.88% |  |  |  |  |  |  |  |  |  |  |
| Ye ZM, 2012[[312](#_ENREF_312)] | 2011/04-2011/07 | Zhejiang | East | Cross-sectional | Entertainment venues | 3 | Chinese | 545 | 2 | 0.37% |  |  |  |  |  |  |  |  |  |  |
| Ye ZM, 2012[[312](#_ENREF_312)] | 2011/04-2011/07 | Zhejiang | East | Cross-sectional | Entertainment venues | 3 | Chinese | 171 | 1 | 0.58% |  |  |  |  |  |  |  |  |  |  |
| Zhu HW, 2012[[313](#_ENREF_313)] | 2011/04-2011/07 | Jiangxi | East | Cross-sectional | Entertainment venues | 4 | Chinese | 400 | 1 | 0.25% |  |  |  |  |  |  |  |  |  |  |
| Jiang DK, 2010a[[200](#_ENREF_200)] | 2009 | Shanxi | North | National sentinel sites | Detaining education center | 6 | Chinese | 380 | 0 | 0.00% |  |  |  |  |  |  |  |  |  |  |
| Kang YF, 2011[[314](#_ENREF_314)] | 2009 | Shanxi | North | Cross-sectional | National sentinel site | 2 | Chinese | 400 | 2 | 0.50% |  |  |  |  |  |  |  |  |  |  |
| Xu YJ, 2011[[201](#_ENREF_201)] | 2009 | Shanxi | North | Cross-sectional | Entertainment venues | 3 | Chinese | 5,513 | 36 | 0.65% |  |  |  |  |  |  |  |  |  |  |
| Xu YJ, 2011[[201](#_ENREF_201)] | 2009 | Shanxi | North | Cross-sectional | Entertainment venues | 3 | Chinese | 4,885 | 34 | 0.70% |  |  |  |  |  |  |  |  |  |  |
| Xu YJ, 2011[[201](#_ENREF_201)] | 2009 | Shanxi | North | Cross-sectional | Entertainment venues | 3 | Chinese | 628 | 2 | 0.36% |  |  |  |  |  |  |  |  |  |  |
| Kang YF, 2011[[314](#_ENREF_314)] | 2009-2010 | Shanxi | North | Cross-sectional | National sentinel site | 2 | Chinese | 722 | 5 | 0.69% |  |  |  |  |  |  |  |  |  |  |
| Kang YF, 2011[[314](#_ENREF_314)] | 2010 | Shanxi | North | Cross-sectional | National sentinel site | 2 | Chinese | 322 | 3 | 0.93% |  |  |  |  |  |  |  |  |  |  |
| Feng N, 2011[[203](#_ENREF_203)] | 2010/04-2010/06 | Shanxi | North | Cross-sectional | National sentinel site | 4 | Chinese | 400 | 1 | 0.25% |  |  |  |  |  |  |  |  |  |  |
| Li BY, 2012[[204](#_ENREF_204)] | 2010/04-2010/07 | Tianjin | North | Cross-sectional | Others | 4 | Chinese | 407 | 2 | 0.49% |  |  |  |  |  |  |  |  |  |  |
| Liu XS, 2012[[315](#_ENREF_315)] | 2010/06-2010/09 | Hebei | North | Cross-sectional | Entertainment venues | 4 | Chinese | 784 | 9 | 1.14% |  |  |  |  |  |  |  |  |  |  |
| Liu ZJ, 2012[[206](#_ENREF_206)] | 2011/04-2011/06 | Hebei | North | Cross-sectional | Entertainment venues | 4 | Chinese | 400 | 9 | 2.25% |  |  |  |  |  |  |  |  |  |  |
| Zhang P, 2012[[207](#_ENREF_207)] |  | Jilin | Northeast | Cross-sectional | Others | 4 | Chinese | 400 | 1 | 0.30% |  |  |  |  |  |  |  |  |  |  |
| Zhang P, 2012[[207](#_ENREF_207)] |  | Jilin | Northeast | Cross-sectional | Others | 4 | Chinese | 400 | 1 | 0.30% |  |  |  |  |  |  |  |  |  |  |
| Xian XJ, 2011[[316](#_ENREF_316)] | 2011 | Jilin | Northeast | Cross-sectional | National sentinel site | 3 | Chinese | 400 | 4 | 1.00% |  |  |  |  |  |  |  |  |  |  |
| Zhou D, 2012[[211](#_ENREF_211)] | 2011 | Liaoning | Northeast | Cross-sectional | National sentinel site | 4 | Chinese | 6,838 | 43 | 0.60% |  |  |  |  |  |  |  |  |  |  |
| Xi SH, 2004[[33](#_ENREF_33)] | 2002/05-2002/07 | Gansu | Northwest | Cross-sectional | Entertainment venues | 7 | Chinese | 88 | 2 | 2.30% |  |  |  |  |  |  |  |  |  |  |
| Ma JX, 2012[[219](#_ENREF_219)] | 2006-2010 | Gansu | Northwest | Cross-sectional | Entertainment venues | 4 | Chinese | 800 | 5 | 0.65% |  |  |  |  |  |  |  |  |  |  |
| Xu YJ, 2011a[[224](#_ENREF_224)] | 2009 | Shaanxi | Northwest | Cross-sectional | Others | 4 | Chinese | 5,513 | 36 | 0.65% |  |  |  |  |  |  |  |  |  |  |
| Hu XQ, 2011[[225](#_ENREF_225)] | 2010 | Gansu | Northwest | Cross-sectional | Others | 4 | Chinese | 400 | 4 | 1.00% |  |  |  |  |  |  |  |  |  |  |
| Wu R, 2012[[226](#_ENREF_226)] | 2010 | Xinjiang | Northwest | Cross-sectional | Entertainment venues | 4 | Chinese | 412 | 1 | 0.24% |  |  |  |  |  |  |  |  |  |  |
| Zhao GD, 2011[[317](#_ENREF_317)] | 2010/04-2010/06 | Shaanxi | Northwest | Cross-sectional | National sentinel site | 4 | Chinese | 412 | 1 | 0.24% |  |  |  |  |  |  |  |  |  |  |
| Zhao GD, 2011[[317](#_ENREF_317)] | 2010/04-2010/06 | Shaanxi | Northwest | Cross-sectional | National sentinel site | 4 | Chinese | 412 | 1 | 0.24% |  |  |  |  |  |  |  |  |  |  |
| Zhang MN, 2011[[318](#_ENREF_318)] | 2010/06 | Shaanxi | Northwest | Cross-sectional | Entertainment venues | 4 | Chinese | 400 | 5 | 1.30% |  |  |  |  |  |  |  |  |  |  |
| Guo H, 2012[[228](#_ENREF_228)] | 2010-2011 | Gansu | Northwest | Cross-sectional | Entertainment venues | 3 | Chinese | 820 | 0 | 0.00% |  |  |  |  |  |  |  |  |  |  |
| Qiu YQ, 2004[[298](#_ENREF_298)] |  | Guangdong | South Central | Cross-sectional | Others | 5 | Chinese | 189 | 0 | 0.00% |  |  |  |  |  |  |  |  |  |  |
| Guo P, 2005[[299](#_ENREF_299)] | 2003/10 - 2003/11 | Guangdong | South Central | Cross-sectional | Detaining education center | 3 | Chinese | 633 | 36 | 5.70% |  |  |  |  |  |  |  |  |  |  |
| Liu C, 2011[[319](#_ENREF_319)] | 2007 | Hubei | South Central | Cross-sectional | Entertainment venues | 3 | Chinese | 444 | 34 | 7.66% |  |  |  |  |  |  |  |  |  |  |
| Lin RL, 2012[[252](#_ENREF_252)] | 2009 | Guangxi | South Central | Cross-sectional | Entertainment venues | 4 | Chinese | 400 | 2 | 0.50% |  |  |  |  |  |  |  |  |  |  |
| Luo MH, 2011[[320](#_ENREF_320)] | 2009 | Hunan | South Central | Cross-sectional | Entertainment venues | 2 | Chinese | 400 | 0 | 0.00% |  |  |  |  |  |  |  |  |  |  |
| Luo MH, 2011[[320](#_ENREF_320)] | 2009 | Hunan | South Central | Cross-sectional | Entertainment venues | 2 | Chinese | 400 | 0 | 0.00% |  |  |  |  |  |  |  |  |  |  |
| Nie ZQ, 2011[[321](#_ENREF_321)] | 2009 | Guangdong | South Central | Cross-sectional | Entertainment venues | 4 | Chinese | 2,388 | 14 | 0.60% |  |  |  |  |  |  |  |  |  |  |
| Nie ZQ, 2011[[321](#_ENREF_321)] | 2009 | Guangdong | South Central | Cross-sectional | Entertainment venues | 4 | Chinese | 2,388 | 14 | 0.60% |  |  |  |  |  |  |  |  |  |  |
| Xiang SB, 2010b[[66](#_ENREF_66)] | 2009 | Hunan | South Central | Cross-sectional | Entertainment venues | 4 | Chinese | 401 | 17 | 4.24% |  |  |  |  |  |  |  |  |  |  |
| Zhang L, 2011[[322](#_ENREF_322)] | 2009 | Henan | South Central | Cross-sectional | Entertainment venues | 4 | Chinese | 395 | 1 | 0.25% |  |  |  |  |  |  |  |  |  |  |
| Zhou JL, 2010a[[323](#_ENREF_323)] | 2009 | Hunan | South Central | National sentinel site | Entertainment venues | 6 | Chinese | 402 | 4 | 1.00% |  |  |  |  |  |  |  |  |  |  |
| Wen XQ, 2010[[324](#_ENREF_324)] | 2009/04-2009/05 | Guangxi | South Central | Cross-sectional | Entertainment venues | 3 | Chinese | 400 | 3 | 0.75% |  |  |  |  |  |  |  |  |  |  |
| Xiang H, 2011[[254](#_ENREF_254)] | 2009/04-2009/06 | Hubei | South Central | Cross-sectional | Entertainment venues | 4 | Chinese | 400 | 7 | 1.75% |  |  |  |  |  |  |  |  |  |  |
| Xiang H, 2011[[254](#_ENREF_254)] | 2009/04-2009/06 | Hubei | South Central | Cross-sectional | Entertainment venues | 4 | Chinese | 146 | 0 | 0.00% |  |  |  |  |  |  |  |  |  |  |
| Xiang H, 2011[[254](#_ENREF_254)] | 2009/04-2009/06 | Hubei | South Central | Cross-sectional | Entertainment venues | 4 | Chinese | 135 | 2 | 1.48% |  |  |  |  |  |  |  |  |  |  |
| Xiang H, 2011[[254](#_ENREF_254)] | 2009/04-2009/06 | Hubei | South Central | Cross-sectional | Entertainment venues | 4 | Chinese | 129 | 5 | 3.88% |  |  |  |  |  |  |  |  |  |  |
| Nei ZQ, 2011a[[325](#_ENREF_325)] | 2009/04-2009/07 | Guangdong | South Central | National sentinel site | VCT | 5 | Chinese | 2,388 | 14 | 0.60% |  |  |  |  |  |  |  |  |  |  |
| Shi JX, 2012[[256](#_ENREF_256)] | 2009/09 | Guangdong | South Central | Cross-sectional | Entertainment venues | 4 | Chinese | 240 | 41 | 17.10% |  |  |  |  |  |  |  |  |  |  |
| Zhou Y, 2012[[260](#_ENREF_260)] | 2009/12 | Guangxi | South Central | Cross-sectional | Entertainment venues | 4 | Chinese | 300 | 2 | 0.70% |  |  |  |  |  |  |  |  |  |  |
| Jiang N, 2012[[261](#_ENREF_261)] | 2009-2010 | Henan | South Central | Cross-sectional | Others | 3 | Chinese | 475 | 40 | 8.42% |  |  |  |  |  |  |  |  |  |  |
| Lin RL, 2012[[252](#_ENREF_252)] | 2009-2011 | Guangxi | South Central | Cross-sectional | Entertainment venues | 4 | Chinese | 1,200 | 11 | 0.92% |  |  |  |  |  |  |  |  |  |  |
| Lin RL, 2012[[252](#_ENREF_252)] | 2010 | Guangxi | South Central | Cross-sectional | Entertainment venues | 4 | Chinese | 400 | 4 | 1.00% |  |  |  |  |  |  |  |  |  |  |
| Xiong CS, 2012[[265](#_ENREF_265)] | 2010 | Hubei | South Central | Cross-sectional | Entertainment venues | 4 | Chinese | 400 | 0 | 0.00% |  |  |  |  |  |  |  |  |  |  |
| Yang JY, 2011[[326](#_ENREF_326)] | 2010 | Guangdong | South Central | Cross-sectional | Others | 2 | Chinese | 299 | 0 | 0.00% |  |  |  |  |  |  |  |  |  |  |
| Zhu L, 2011[[70](#_ENREF_70)] | 2010/04-2010/06 | Hubei | South Central | Cross-sectional | National sentinel site | 4 | Chinese | 402 | 5 | 1.20% |  |  |  |  |  |  |  |  |  |  |
| Chen ZB, 2011[[266](#_ENREF_266)] | 2010/04-2010/07 | Guangdong | South Central | Cross-sectional | Entertainment venues | 3 | Chinese | 200 | 0 | 0.00% |  |  |  |  |  |  |  |  |  |  |
| Chen ZB, 2011[[266](#_ENREF_266)] | 2010/04-2010/07 | Guangdong | South Central | Cross-sectional | Entertainment venues | 3 | Chinese | 177 | 0 | 0.00% |  |  |  |  |  |  |  |  |  |  |
| Gui Q, 2012[[327](#_ENREF_327)] | 2010/04-2010/07 | Hunan | South Central | Cross-sectional | Entertainment venues | 4 | Chinese | 400 | 5 | 1.25% |  |  |  |  |  |  |  |  |  |  |
| Bai Y, 2012[[328](#_ENREF_328)] | 2010/05-2010/07 | Guangxi | South Central | Cross-sectional | Others | 4 | Chinese | 1,846 | 10 | 0.50% |  |  |  |  |  |  |  |  |  |  |
| Bai Y, 2012[[328](#_ENREF_328)] | 2010/05-2010/07 | Guangxi | South Central | Cross-sectional | Others | 4 | Chinese | 506 | 2 | 0.40% |  |  |  |  |  |  |  |  |  |  |
| Bai Y, 2012[[328](#_ENREF_328)] | 2010/05-2010/07 | Guangxi | South Central | Cross-sectional | Others | 4 | Chinese | 793 | 5 | 0.63% |  |  |  |  |  |  |  |  |  |  |
| Bai Y, 2012[[328](#_ENREF_328)] | 2010/05-2010/07 | Guangxi | South Central | Cross-sectional | Others | 4 | Chinese | 547 | 3 | 0.55% |  |  |  |  |  |  |  |  |  |  |
| Li Y, 2012[[267](#_ENREF_267)] | 2010-2011 | Hubei | South Central | Cross-sectional | Entertainment venues | 4 | Chinese | 400 | 1 | 0.25% |  |  |  |  |  |  |  |  |  |  |
| Lin RL, 2012[[252](#_ENREF_252)] | 2011 | Guangxi | South Central | Cross-sectional | Entertainment venues | 4 | Chinese | 400 | 5 | 1.25% |  |  |  |  |  |  |  |  |  |  |
| Tang J, 2012[[269](#_ENREF_269)] | 2011 | Guangxi | South Central | Cross-sectional | Entertainment venues | 4 | Chinese | 400 | 2 | 0.50% |  |  |  |  |  |  |  |  |  |  |
| Tang MJ, 2012[[268](#_ENREF_268)] | 2011 | Guangxi | South Central | Cross-sectional | Entertainment venues | 4 | Chinese | 1,213 | 13 | 1.10% |  |  |  |  |  |  |  |  |  |  |
| Ke XZ, 2012[[270](#_ENREF_270)] | 2011/04-2011/07 | Hubei | South Central | Cross-sectional | Others | 3 | Chinese | 400 | 2 | 0.50% |  |  |  |  |  |  |  |  |  |  |
| Quan XB, 2012[[271](#_ENREF_271)] | 2011/05-2011/07 | Guangxi | South Central | Cross-sectional | Entertainment venues | 4 | Chinese | 400 | 5 | 1.25% |  |  |  |  |  |  |  |  |  |  |
| Chen RC, 2004[[301](#_ENREF_301)] |  | Guangdong | South Central | Cross-sectional | Entertainment venues | 4 | Chinese | 52 | 5 | 9.60% |  |  |  |  |  |  |  |  |  |  |
| Guo HJ, 2012[[272](#_ENREF_272)] |  | Guizhou | Southwest | Cross-sectional | Others | 4 | Chinese | 235 | 16 | 16.80% |  |  |  |  |  |  |  |  |  |  |
| Wang GH, 2011[[329](#_ENREF_329)] |  | Sichuan | Southwest | Cross-sectional | Entertainment venues | 4 | Chinese | 354 | 3 | 0.85% |  |  |  |  |  |  |  |  |  |  |
| Yan WZ, 2010a[[330](#_ENREF_330)] |  | Yunnan | Southwest | Cross-sectional | Entertainment venues | 2 | Chinese | 188 | 1 | 0.53% |  |  |  |  |  |  |  |  |  |  |
| Ngo TD, 2008[[293](#_ENREF_293)] | 2004 | Yunnan | Southwest | Cross-sectional | Detaining education center | 3 | English | 310 | 236 | 76.13% |  |  |  |  |  |  |  |  |  |  |
| Lei ZQ, 2005[[273](#_ENREF_273)] | 2004/04 | Sichuan | Southwest | Cross-sectional | Entertainment venues | 4 | Chinese | 259 | 5 | 1.93% |  |  |  |  |  |  |  |  |  |  |
| Li DM, 2007a[[276](#_ENREF_276)] | 2006 | Guizhou | Southwest | Cross-sectional | Entertainment venues | 7 | Chinese | 432 | 21 | 4.86% |  |  |  |  |  |  |  |  |  |  |
| Li DM, 2007b[[276](#_ENREF_276)] | 2006 | Guizhou | Southwest | Cross-sectional | Entertainment venues | 7 | Chinese | 220 | 7 | 3.18% |  |  |  |  |  |  |  |  |  |  |
| Li DM, 2007d[[276](#_ENREF_276)] | 2006 | Guizhou | Southwest | Cross-sectional | Entertainment venues | 7 | Chinese | 212 | 14 | 6.60% |  |  |  |  |  |  |  |  |  |  |
| Li YK, 2012[[331](#_ENREF_331)] | 2009 | Sichuan | Southwest | Cross-sectional | Others | 2 | Chinese | 690 | 7 | 1.01% |  |  |  |  |  |  |  |  |  |  |
| Yan WZ, 2011[[94](#_ENREF_94)] | 2009 | Yunnan | Southwest | Cross-sectional | Entertainment venues | 3 | Chinese | 180 | 1 | 0.56% |  |  |  |  |  |  |  |  |  |  |
| Wang WW, 2010a[[332](#_ENREF_332)] | 2009/04 | Sichuan | Southwest | National sentinel site | VCT | 5 | Chinese | 1,156 | 19 | 1.64% |  |  |  |  |  |  |  |  |  |  |
| He B, 2011[[333](#_ENREF_333)] | 2009/04-2009/05 | Yunnan | Southwest | Cross-sectional | Entertainment venues | 4 | Chinese | 206 | 3 | 1.63% |  |  |  |  |  |  |  |  |  |  |
| Chen C, 2010[[280](#_ENREF_280)] | 2009-2010 | Sichuan | Southwest | Cross-sectional | Entertainment venues | 4 | Chinese | 403 | 12 | 2.98% |  |  |  |  |  |  |  |  |  |  |
| Zhang H, 2011[[334](#_ENREF_334)] | 2009-2010 | Sichuan | Southwest | Cross-sectional | Others | 2 | Chinese | 629 | 6 | 1.00% |  |  |  |  |  |  |  |  |  |  |
| Li YK, 2012[[331](#_ENREF_331)] | 2010 | Sichuan | Southwest | Cross-sectional | Others | 2 | Chinese | 1,203 | 8 | 0.67% |  |  |  |  |  |  |  |  |  |  |
| Zhou CX, 2011[[335](#_ENREF_335)] | 2010 | Guizhou | Southwest | Cross-sectional | National sentinel site | 4 | Chinese | 235 | 16 | 6.80% |  |  |  |  |  |  |  |  |  |  |
| Ci Ren WM, 2012[[336](#_ENREF_336)] | 2010/06-2010/07 | Tibet | Southwest | Cross-sectional | Entertainment venues | 3 | Chinese | 442 | 4 | 0.90% |  |  |  |  |  |  |  |  |  |  |
| Li YK, 2012[[331](#_ENREF_331)] | 2011 | Sichuan | Southwest | Cross-sectional | Others | 2 | Chinese | 1,228 | 13 | 1.06% |  |  |  |  |  |  |  |  |  |  |
| Li YK, 2012[[331](#_ENREF_331)] | 2011 | Sichuan | Southwest | Cross-sectional | Others | 2 | Chinese | 1,228 | 13 | 1.06% |  |  |  |  |  |  |  |  |  |  |
| Zhang H, 2012[[281](#_ENREF_281)] | 2011 | Yunnan | Southwest | Cross-sectional | Others | 2 | Chinese | 1,301 | 4 | 0.31% |  |  |  |  |  |  |  |  |  |  |
| Ou YH, 2012[[337](#_ENREF_337)] | 2011/04-2011/06 | Sichuan | Southwest | Cross-sectional | Entertainment venues | 4 | Chinese | 400 | 3 | 0.75% |  |  |  |  |  |  |  |  |  |  |
|  |  |  |  |  |  |  |  |  |  |  |  |  |  |  |  |  |  |  |  |  |

**Table S2. Systematic review of 19 studies reporting the co-infection prevalence of sexually transmitted infections and/or viral hepatitis infections among HIV-positive female sex workers in China.**

| Study | Year | Province | Region | Sampling method | Venue | Quality score | Language | Disease Prevalence | | |
| --- | --- | --- | --- | --- | --- | --- | --- | --- | --- | --- |
|  |  |  |  |  |  |  |  | Sample size, N | n | Prevalence (%) |
| *a) Chlamydia* | | | | | | | | | | |
| Wang H, 2009[[84](#_ENREF_84)] | 2006/03-2006/05 | Yunnan | Southwest | Cross-sectional | Others | 4 | English | 76 | 14 | 18.42% |
| Jin X, 2011[[93](#_ENREF_93)] | 2008/04-2008/05 | Yunnan | Southwest | Cross-sectional | Entertainment venues | 5 | English | 63 | 14 | 22.58% |
| b)Syphilis |  |  |  |  |  |  |  |  |  |  |
| Luo J, 2005[[338](#_ENREF_338)] | 2004/11 | Guangxi | South Central | Cross-sectional | Entertainment venues | 6 | Chinese | 11 | 5 | 45.45% |
| Chen XS, 2012 (a)[[132](#_ENREF_132)] | 2009/06-2009/09 |  | South Central | Cross-sectional | Entertainment venues | 7 | English | 28 | 5 | 17.86% |
| Chen XS, 2012 (a)[[167](#_ENREF_167)] | 2009/06-2009/09 | Jiangsu, Guangxi, Guangdong, Hainan | South Central | Cross-sectional | Entertainment venues | 6 | English | 43 | 6 | 13.95% |
| Chen XS, 2005[[339](#_ENREF_339)] | 2000 | Yunnan | Southwest | Cross-sectional | Detaining education center | 5 | English | 52 | 5 | 9.62% |
| Wang H, 2009[[84](#_ENREF_84)] | 2006/03-2006/05 | Yunnan | Southwest | Cross-sectional | Entertainment venues | 4 | English | 76 | 6 | 7.89% |
| c) Trichomonas vaginitis | | | | | | | | | | |
| Wang H, 2009[[84](#_ENREF_84)] | 2006/03-2006/05 | Yunnan | Southwest | Cross-sectional | Others | 4 | English | 76 | 15 | 19.74% |
| d) HSV | | | | | | | | | | |
| Wang H, 2009[[84](#_ENREF_84)] | 2006/03-2006/05 | Yunnan | Southwest | Cross-sectional | Others | 4 | English | 76 | 66 | 86.84% |
| Chen XS, 2005[[339](#_ENREF_339)] | 2000 | Yunnan | Southwest | Cross-sectional | Detaining education center | 5 | English | 52 | 41 | 78.85% |

**Table S3. Heterogeneity in subgroup meta-analyses**

| **Subgroups** | **No. of studies** | | **No. of estimates** | **Pooled Prevalence (%) and 95% CI** | **Heterogeneity** | **Publication bias** | **Total No of infection/Sample size** | **Meta-regression coefficient (95% CI)** | **Standard Error** | **Odds Ratio (95% CI)** |
| --- | --- | --- | --- | --- | --- | --- | --- | --- | --- | --- |
|  |  |  |  |  | ***I^2^*** | **Begg’s**  **(p values)** |  |  |  |  |
| ***a)Chlamydia*** | | 96 | 125 | 13.66(12.11-15.37) | 96.720 | 0.000 | 7983/56548 | - | - | - |
| **Region** | |  |  |  |  |  |  |  |  |  |
| East | | 27 | 31 | 11.86(9.36-14.91) | 97.276 | - | 1436/11370 | Ref. | Ref. | Ref. |
| North | | 2 | 5 | 4.51 (2.77-7.28) | 97.515 | - | 273/4470 | -0.95(-2.19-0.29) | 0.62 | 0.39 (0.11-1.33) |
| Northeast | | 1 | 1 | 0.5 (0.36-0.68) | 0.000 | - | 38/7662 | -1.93(-4.48-0.62) | 1.29 | 0.14 (0.01-1.86) |
| Northwest | | 9 | 9 | 13.71 (9.0-20.26) | 95.362 | - | 424/2424 | -0.1(-0.95-0.74) | 0.43 | 0.9 (0.39-2.1) |
| Southcentral | | 31 | 39 | 14.18(11.61-17.21) | 95.032 | - | 1285/10894 | 0.08(-0.5-0.65) | 0.29 | 1.08 (0.61-1.91) |
| Southwest | | 26 | 39 | 18.95 (15.76-22.63) | 94.567 | - | 4519/20254 | 0.13(-0.5-0.76) | 0.32 | 1.14 (0.61-2.14) |
| **Venue** | |  |  |  |  |  |  |  |  |  |
| Entertainment venues | | 72 | 94 | 15.61(13.75-17.69) | 95.188 | - | 6290/36877 | Ref. | Ref. | Ref. |
| National sentinel site | | 3 | 6 | 2.50(1.28-4.80) | 97.789 | - | 129/9576 | -0.55(-1.99-0.88) | 0.73 | 0.57 (0.14-2.42) |
| Detaining education center | | 16 | 20 | 8.94(6.48-12.22) | 97.932 | - | 856/7252 | -0.43(-1.09-0.22) | 0.33 | 0.65 (0.34-1.25) |
| Others | | 5 | 5 | 22.93(13.27-36.67) | 98.067 | - | 708/2843 | 0.86(-0.26-1.97) | 0.56 | 2.35 (0.77-7.16) |
| **Sampling size** | |  |  |  |  | - |  |  |  |  |
| <400 | | 58 | 78 | 15.82(13.57-18.38) | 93.938 | - | 2723/15595 | Ref. | Ref. | Ref. |
| ≥400 | | 38 | 47 | 10.88(8.94-13.19) | 98.106 | - | 5260/40953 | -0.52(-0.96--0.08) | 0.22 | 0.6 (0.38-0.92)* |
| **Language** | |  |  |  |  | - |  |  |  |  |
| Chinese | | 79 | 101 | 12.15(10.65-13.84) | 96.557 | - | 1429/14523 | Ref. | Ref. | Ref. |
| English | | 17 | 24 | 21.24(16.79-26.49) | 95.533 | - | 6554/42025 | 0.62(0.03-1.21) | 0.30 | 1.86 (1.03-3.35) |
| Quality score (continuous) | | 96 | 125 | 13.66(12.11-15.37) | 96.720 | - | 7983/56548 | 0.37(-0.08-0.81) | 0.22 | 1.44 (0.93-2.25) |
| Study period (continuous) | | 96 | 125 | 13.66(12.11-15.37) | 96.720 | - | 7983/56548 | -0.09(-0.17--0.01) | 0.04 | 0.91 (0.84-0.99)* |
|  | |  |  |  |  |  |  |  |  |  |
| ***b)Gonorrhea*** | | 116 | 149 | 6.07(5.29-6.95) | 94.694 | 0.000 | 3723/62430 | - | - | - |
| **Region** | |  |  |  |  |  |  |  |  |  |
| East | | 33 | 43 | 6.88(5.68-8.31) | 93.719 | - | 2099/31525 | Ref. | Ref. | Ref. |
| North | | 7 | 12 | 12.69(7.52-20.60) | 96.154 | - | 311/1806 | -0.36(-1.3-0.58) | 0.48 | 0.7 (0.27-1.79) |
| Northeast | | 1 | 1 | 5.45 (4.22-7.01) | 94.650 | - | 653/13678 | -1.39(-3.9-1.12) | 1.27 | 0.25 (0.02-3.06) |
| Northwest | | 10 | 10 | 4.56 (2.73-7.52) | 96.156 | - | 411/5607 | -0.01(-0.83-0.81) | 0.41 | 0.99 (0.44-2.25) |
| Southcentral | | 36 | 42 | 1.46 (0.28-7.19) | 0.000 | - | 98/6462 | 0.26(-0.27-0.79) | 0.27 | 1.3 (0.76-2.21) |
| Southwest | | 32 | 40 | 5.46 (3.18-9.23) | 84.759 | - | 126/2036 | 0.18(-0.4-0.76) | 0.29 | 1.19 (0.67-2.13) |
| **Venue** | |  |  |  |  |  |  |  |  |  |
| Entertainment venues | | 87 | 107 | 5.93(5.10-6.89) | 91.128 | - | 2903/44262 | Ref. | Ref. | Ref. |
| National sentinel site | | 2 | 5 | 4.45(2.13-9.06) | 98.541 | - | 231/9174 | 0.83(-0.57-2.22) | 0.71 | 2.28 (0.56-9.25) |
| Detaining education center | | 21 | 31 | 7.94(5.91-10.60) | 96.667 | - | 1086/11672 | 0.6(0.04-1.16) | 0.28 | 1.82 (1.04-3.19)* |
| Others | | 5 | 5 | 4.69(2.21-9.69) | 89.298 | - | 258/3443 | 0.04(-1.06-1.14) | 0.56 | 1.04 (0.35-3.14) |
| VCT | | 1 | 1 | 2.08(0.37-10.81) | 0.000 | - | 8/385 | -1.12(-3.32-1.09) | 1.11 | 0.33 (0.04-2.96) |
| **Sampling size** | |  |  |  |  | - |  |  |  |  |
| <400 | | 76 | 105 | 6.70(5.68-7.89) | 90.661 | - | 1885/22556 | Ref. | Ref. | Ref. |
| ≥400 | | 40 | 44 | 5.21(4.15-6.52) | 96.919 | - | 2601/46380 | -0.59(-1.01--0.17) | 0.21 | 0.55 (0.36-0.84)* |
| **Language** | |  |  |  |  | - |  |  |  |  |
| Chinese | | 98 | 125 | 5.63(4.83-6.55) | 94.377 | - | 3280/53977 | Ref. | Ref. | Ref. |
| English | | 18 | 24 | 8.56(6.21-11.70) | 95.866 | - | 1206/14959 | 0.7(0.13-1.28) | 0.29 | 2.01 (1.13-3.58) |
| Quality score (continuous) | | 116 | 149 | 6.07(5.29-6.95) | 94.694 | - | 4486/68936 | 0.24(-0.15-0.64) | 0.20 | 1.28 (0.86-1.9) |
| Study period (continuous) | | 116 | 149 | 6.07(5.29-6.95) | 94.694 | - | 4486/68936 | -0.02(-0.09-0.05) | 0.03 | 0.98 (0.92-1.05) |
|  | |  |  |  |  |  |  |  |  |  |
| ***c)Syphilis*** | | 215 | 377 | 5.24(4.83-5.69) | 95.43 | 0.000 | 13507/245455 | - | - | - |
| **Region** | |  |  |  |  |  |  |  |  |  |
| East | | 75 | 122 | 5.27(4.62-6.01) | 94.297 | - | 5217/94510 | Ref. | Ref. | Ref. |
| North | | 19 | 30 | 2.67(1.95-3.66) | 93.544 | - | 495/23167 | -0.73(-1.14--0.32) | 0.21 | 0.48 (0.32-0.73) |
| Northeast | | 6 | 13 | 4.19(2.65-6.57) | 97.634 | - | 329/11377 | 0.02(-0.56-0.61) | 0.30 | 1.02 (0.57-1.84) |
| Northwest | | 20 | 36 | 1.95(1.25-3.02) | 93.277 | - | 512/19193 | -0.64(-1.04--0.25) | 0.20 | 0.52 (0.35-0.78) |
| Southcentral | | 60 | 121 | 8.33(6.96-9.94) | 96.482 | - | 5202/72445 | 0.24(-0.01-0.49) | 0.13 | 1.27 (0.99-1.63) |
| Southwest | | 31 | 51 | 6.49(5.22-8.04) | 88.804 | - | 1752/24763 | 0.24(-0.12-0.59) | 0.18 | 1.27 (0.89-1.8) |
| **Venue** | |  |  |  |  |  |  |  |  |  |
| Entertainment venues | | 149 | 246 | 5.13(4.66-5.64) | 94.425 | - | 6971/134958 | Ref. | Ref. | Ref. |
| National sentinel site | | 8 | 15 | 1.95(1.25-3.02) | 87.915 | - | 232/13516 | -0.5(-1.06-0.05) | 0.28 | 0.6 (0.35-1.05) |
| Detaining education center | | 40 | 68 | 8.40(7.00-10.05) | 95.101 | - | 3559/35583 | 0.65(0.35-0.95) | 0.15 | 1.91 (1.42-2.58)* |
| Others | | 18 | 48 | 4.23(3.37-5.30) | 96.550 | - | 2745/61398 | -0.04(-0.37-0.28) | 0.17 | 0.96 (0.69-1.33) |
| **Sampling size** | |  |  |  |  |  |  |  |  |  |
| <400 | | 108 | 218 | 6.01(5.37-6.73) | 91.927 | - | 3563/49332 | Ref. | Ref. | Ref. |
| ≥400 | | 107 | 159 | 4.30(3.81-4.85) | 97.079 | - | 9944/196123 | -0.3(-0.51--0.09) | 0.11 | 0.74 (0.6-0.92)* |
| **Language** | |  |  |  |  | - |  |  |  |  |
| Chinese | | 186 | 328 | 5.16(4.72-5.63) | 95.83583026 | - | 11306/209383 | Ref. | Ref. | Ref. |
| English | | 29 | 49 | 5.86(4.64-7.37) | 89.80061126 | - | 2201/36072 | 0.15(-0.22-0.52) | 0.19 | 1.16 (0.8-1.69) |
| Quality score (continuous) | | 215 | 377 | 5.24(4.83-5.69) | 95.43 | - | 13507/245455 | -0.07(-0.31-0.17) | 0.12 | 0.93 (0.73-1.19) |
| Study period (continuous) | | 215 | 377 | 5.24(4.83-5.69) | 95.43 | - | 13507/245455 | -0.02(-0.05-0.01) | 0.02 | 0.98 (0.95-1.0)* |
|  | |  |  |  |  |  |  |  |  |  |
| ***d)Trichomonas vaginitis*** | | 43 | 65 | 8.35 (6.77-10.27) | 96.393 | 0.074 | 2271/24381 | - | - | - |
| **Region** | |  |  |  |  |  |  |  |  |  |
| East | | 11 | 11 | 4.68(2.65-8.15) | 91.610 | - | 175/2823 | Ref. | Ref. | Ref. |
| North | | 1 | 1 | 1.89(0.47-7.23) | 0.000 | - | 2/106 | -1.19(-4.2-1.82) | 1.50 | 0.3 (0.02-6.15) |
| Northwest | | 4 | 4 | 5.16(2.41-10.72) | 75.242 | - | 119/1994 | 1.02(-0.39-2.43) | 0.70 | 2.76 (0.67-11.33) |
| Southcentral | | 10 | 12 | 5.85(3.51-9.60) | 95.402 | - | 341/5630 | 0.99(-0.09-2.06) | 0.54 | 2.68 (0.91-7.87) |
| Southwest | | 17 | 36 | 11.80(9.07-15.2) | 97.131 | - | 1621/13548 | 1.23(0.36-2.1) | 0.43 | 3.41 (1.43-8.15)* |
| **Venue** | |  |  |  |  |  |  |  |  |  |
| Entertainment venues | | 32 | 51 | 7.75(6.19-9.66) | 95.450 | - | 1379/17929 | Ref. | Ref. | Ref. |
| Detaining education center | | 8 | 11 | 13.16(7.89-21.13) | 97.381 | - | 629/3739 | 0.99(0.03-1.95) | 0.48 | 2.7 (1.03-7.05)* |
| Others | | 3 | 3 | 8.70(3.30-21.02) | 71.422 | - | 263/2713 | 0.21(-1.12-1.55) | 0.66 | 1.24 (0.33-4.69) |
| **Sampling size** | |  |  |  |  |  |  |  |  |  |
| <400 | | 27 | 43 | 9.81(7.23-13.19) | 95.633 | - | 1155/9000 | Ref. | Ref. | Ref. |
| ≥400 | | 16 | 22 | 7.52(5.61-10.01) | 96.837 | - | 1116/15381 | -0.9(-1.67--0.12) | 0.39 | 0.41 (0.19-0.89)* |
| **Language** | |  |  |  |  | - |  |  |  |  |
| Chinese | | 34 | 51 | 8.40(6.57-10.69) | 96.099 | - | 1428/14523 | Ref. | Ref. | Ref. |
| English | | 9 | 14 | 10.32(6.74-15.49) | 97.177 | - | 843/9858 | 0.55(-0.34-1.44) | 0.44 | 1.73 (0.71-4.22) |
| Quality score (continuous) | | 43 | 65 | 8.35 (6.77-10.27) | 96.393 | - | 2271/24381 | 0.08(-0.54-0.7) | 0.31 | 1.08 (0.58-2.02) |
| Study period (continuous) | | 43 | 65 | 8.35 (6.77-10.27) | 96.393 | - | 2271/24381 | 0.05(-0.08-0.18) | 0.07 | 1.05 (0.92-1.2) |
|  | |  |  |  |  |  |  |  |  |  |
| ***e) Genital warts*** | | 68 | 76 | 3.27(2.53-4.21) | 94.222 | 0.001 | 1111/39862 | - | - | - |
| **Region** | |  |  |  |  |  |  |  |  |  |
| East | | 20 | 21 | 3.55(2.11-5.92) | 91.379 | - | 207/4446 | Ref. | Ref. | Ref. |
| North | | 2 | 2 | 1.43(0.57-3.57) | 90.371 | - | 11/617 | -0.74(-2.24-0.76) | 0.75 | 0.48 (0.11-2.13) |
| Northeast | | 1 | 1 | 2.19 (0.25-16.81) | 0.000 | - | 168/7662 | -0.93(-4.7-2.84) | 1.89 | 0.4 (0.01-17.09) |
| Northwest | | 6 | 6 | 1.71(1.14-2.56) | 0.000 | - | 23/1480 | -0.56(-1.93-0.81) | 0.68 | 0.57 (0.15-2.24) |
| Southcentral | | 23 | 25 | 4.04(2.56-6.32) | 87.990 | - | 422/8668 | 0.43(-0.52-1.37) | 0.47 | 1.53 (0.59-3.95) |
| Southwest | | 15 | 20 | 3.09 (1.82-5.18) | 96.485 | - | 280/16989 | -0.08(-1.01-0.86) | 0.47 | 0.93 (0.36-2.35) |
| **Venue** | |  |  |  |  |  |  |  |  |  |
| Entertainment venues | | 57 | 65 | 3.21(2.38-4.32) | 93.765 | - | 647/27892 | Ref. | Ref. | Ref. |
| National sentinel site | | 1 | 1 | 2.19(0.23-18.08) | 0.000 | - | 168/7662 | 0.45(-0.65-1.55) | 0.55 | 1.56 (0.52-4.69) |
| Detaining education center | | 8 | 8 | 3.10(1.60-5.92) | 91.866 | - | 227/3380 | 1.82(-0.99-4.64) | 1.41 | 6.18 (0.37-103.1) |
| Others | | 2 | 2 | 5.74(1.13-24.56) | 61.067 | - | 69/928 | 0.95(-2.51-4.42) | 1.74 | 2.6 (0.08-82.73) |
| **Sampling size** | |  |  |  |  |  |  |  |  |  |
| <400 | | 54 | 62 | 4.26(3.38-5.34) | 90.83 | - | 571/12483 | Ref. | Ref. | Ref. |
| ≥400 | | 14 | 14 | 1.94(1.16-3.20) | 97.026 | - | 540/27379 | -1.21(-2.19--0.22) | 0.49 | 0.3 (0.11-0.8)* |
| **Language** | |  |  |  |  |  |  |  |  |  |
| Chinese | | 64 | 72 | 3.30(2.52-4.30) | 94.359 | - | 1021/37732 | Ref. | Ref. | Ref. |
| English | | 4 | 4 | 2.46(0.78-7.51) | 91.575 | - | 90/2130 | 0.21(-1.36-1.78) | 0.79 | 1.23 (0.26-5.92) |
| Quality score (continuous) | | 68 | 76 | 3.27(2.53-4.21) | 94.222 | - | 1111/39862 | -0.17(-1-0.66) | 0.41 | 0.84 (0.37-1.93) |
| Study period (continuous) | | 68 | 76 | 3.27(2.53-4.21) | 94.222 | - | 1111/39862 | -0.03(-0.15-0.08) | 0.06 | 0.97 (0.86-1.08) |
|  | |  |  |  |  |  |  |  |  |  |
| ***f) HSV*** | | 38 | 57 | 15.77(11.74-20.85) | 99.002 | 0.007 | 9529/30345 | - | - | - |
| **Region** | |  |  |  |  |  |  |  |  |  |
| East | | 6 | 8 | 17.85(10.91-27.82) | 96.388 | - | 614/2128 | Ref. | Ref. | Ref. |
| Northeast | | 1 | 1 | 2.19(1.89-2.55) | 0.000 | - | 168/7662 | -0.03(-4.36-4.31) | 2.15 | 0.98 (0.01-74.74) |
| Northwest | | 1 | 1 | 0.2(0.01-3.09) | 0.000 | - | 1/251 | -3.36(-8.12-1.39) | 2.36 | 0.03 (0-4.02) |
| Southcentral | | 12 | 15 | 3.82(2.5-5.79) | 95.969 | - | 299/4901 | -1.79(-3.51--0.06) | 0.86 | 0.17 (0.03-0.94)* |
| Southwest | | 18 | 32 | 41.83(35.3-48.67) | 97.487 | - | 8447/15403 | 0.33(-1.25-1.92) | 0.79 | 1.4 (0.29-6.81) |
| **Venue** | |  |  |  |  |  |  |  |  |  |
| Entertainment venues | | 29 | 48 | 16.77(12.86-21.56) | 98.052 | - | 7239/17202 | Ref. | Ref. | Ref. |
| National sentinel site | | 1 | 1 | 2.19(0.28-15.13) | 0.000 | - | 168/7662 | -1(-2.89-0.89) | 0.94 | 0.37 (0.06-2.45) |
| Detaining education center | | 4 | 4 | 20.37(8.62-40.98) | 98.906 | - | 357/1878 | 0.68(-1.42-2.78) | 1.04 | 1.97 (0.24-16.05) |
| Others | | 4 | 4 | 32.87(14.71-58.17) | 99.385 | - | 1765/3603 | 1.68(-2.45-5.81) | 1.68 | 5.36 (0.09-332.89) |
| **Sampling size** | |  |  |  |  |  |  |  |  |  |
| <400 | | 19 | 33 | 9.41(5.82-14.87) | 97.555 | - | 1502/6265 | Ref. | Ref. | Ref. |
| ≥400 | | 19 | 24 | 30.56(20.88-42.33) | 99.510 | - | 8027/24080 | 0.74(-0.64-2.13) | 0.74 | 2.1 (0.53-8.4) |
| **Language** | |  |  |  |  |  |  |  |  |  |
| Chinese | | 27 | 39 | 7.92(5.46-11.36) | 99.045 | - | 2731/19043 | Ref. | Ref. | Ref. |
| English | | 11 | 18 | 51.29(38.34-64.09) | 97.525 | - | 6798/11302 | 0.92(-0.34-2.18) | 0.92 | 2.51 (0.71-8.8) |
| Quality score (continuous) | | 38 | 57 | 15.77(11.74-20.85) | 99.002 | - | 9529/30345 | 1.69(0.51-2.87) | 0.58 | 5.41 (1.67-17.57)* |
| Study period (continuous) | | 38 | 57 | 15.77(11.74-20.85) | 99.002 | - | 9529/30345 | -0.25(-0.49--0.01) | 0.12 | 0.78 (0.61-0.99)* |
|  | |  |  |  |  |  |  |  |  |  |
| ***g) HBV*** | | 18 | 24 | 10.71(7.30-15.48) | 97.064 | 0.047 | 848/7614 | - | - | - |
| **Region** | |  |  |  |  |  |  |  |  |  |
| East | | 8 | 14 | 6.08 (3.99-9.16) | 81.705 | - | 297/4049 | Ref. | Ref. | Ref. |
| Northwest | | 1 | 1 | 10.74(7.70-14.79) | 65.430 | - | 32/298 | 0.02(-1.95-1.99) | 0.90 | 1.02 (0.14-7.29) |
| Southcentral | | 8 | 8 | 15.49 (9.53-24.19) | 97.211 | - | 490/3008 | 0.29(-0.41-1) | 0.32 | 1.34 (0.67-2.71) |
| Southwest | | 1 | 1 | 11.19(2.60-37.34) | 0.000 | - | 29/259 | 0.58(-0.79-1.94) | 0.63 | 1.78 (0.46-6.98) |
| **Venue** | |  |  |  |  |  |  |  |  |  |
| Entertainment venues | | 10 | 15 | 9.56(4.32-9.83) | 81.398 | - | 264/3497 | Ref. | Ref. | Ref. |
| Detaining education center | | 3 | 4 | 12.13(5.79-23.67) | 80.972 | - | 343/2499 | 0.19(-0.76-1.15) | 0.44 | 1.21 (0.47-3.14) |
| Others | | 5 | 5 | 14.63(7.62-26.27) | 98.524 | - | 241/1618 | -0.58(-1.65-0.49) | 0.49 | 0.56 (0.19-1.64) |
| **Sampling size** | |  |  |  |  |  |  |  |  |  |
| <400 | | 13 | 17 | 8.09(4.67-13.65) | 95.624 | - | 381/3650 | Ref. | Ref. | Ref. |
| ≥400 | | 5 | 7 | 10.27(7.58-13.79) | 90.435 | - | 467/3964 | 0.67(0.01-1.33) | 0.30 | 1.96 (1.01-3.8)* |
| **Language** | |  |  |  |  |  |  |  |  |  |
| Chinese | | 18 | 24 | 10.71(7.30-15.48) | 97.064 | - | 848/7614 | Ref. | Ref. | Ref. |
| English | | 0 | 0 | 0 | 0.000 | - | 0 | 0 | 0.00 | 0 |
| Quality score (continuous) | | 18 | 24 | 10.71(7.30-15.48) | 97.064 | - | 848/7614 | -0.44(-1.19-0.32) | 0.36 | 0.65 (0.3-1.38) |
| Study period (continuous) | | 18 | 24 | 10.71(7.30-15.48) | 97.064 | - | 848/7614 | -0.03(-0.21-0.15) | 0.08 | 0.97 (0.81-1.16) |
|  | |  |  |  |  |  |  |  |  |  |
| ***h) HCV*** | | 97 | 149 | 0.96 (0.73-1.25) | 94.810 | 0.872 | 1366/114722 | - | - | - |
| **Region** | |  |  |  |  |  |  |  |  |  |
| East | | 36 | 65 | 0.88 (0.6-1.29) | 75.400 | - | 403/47587 | Ref. | Ref. | Ref. |
| North | | 7 | 11 | 0.63 (0.25-1.57) | 45.300 | - | 103/14841 | -0.31(-0.94-0.33) | 0.32 | 0.73 (0.39-1.38) |
| Northeast | | 3 | 4 | 0.49 (0.10-2.29) | 31.500 | - | 49/8038 | -0.16(-1.46-1.14) | 0.66 | 0.86 (0.23-3.14) |
| Northwest | | 8 | 9 | 0.55 (0.19-1.56) | 69.400 | - | 55/9257 | -0.44(-1.21-0.33) | 0.39 | 0.64 (0.3-1.39) |
| Southcentral | | 27 | 39 | 1.01 (0.62-1.64) | 81.400 | - | 314/23488 | 0.24(-0.17-0.65) | 0.21 | 1.28 (0.85-1.92) |
| Southwest | | 16 | 21 | 1.98 (1.07-3.62) | 97.900 | - | 412/11511 | 0.54(0.03-1.05) | 0.26 | 1.72 (1.03-2.87)* |
| **Venue** | |  |  |  |  |  |  |  |  |  |
| Entertainment venues | | 61 | 93 | 0.96(0.71-1.29) | 87.245 | - | 517/48657 | Ref. | Ref. | Ref. |
| National sentinel site | | 11 | 20 | 0.87(0.45-1.67) | 80.358 | - | 107/13415 | 0.3(-0.26-0.87) | 0.29 | 1.36 (0.77-2.39) |
| Detaining education center | | 6 | 6 | 5.14(1.73-14.3) | 99.182 | - | 304/2701 | 0.47(-0.42-1.35) | 0.45 | 1.59 (0.66-3.85) |
| Others | | 16 | 27 | 0.90(0.53-1.50) | 94.213 | - | 388/45244 | -0.1(-0.59-0.39) | 0.25 | 0.91 (0.56-1.48) |
| VCT | | 3 | 3 | 0.51(0.096-2.63) | 86.413 | - | 33/5105 | -0.58(-1.8-0.64) | 0.62 | 0.56 (0.16-1.89) |
| **Sampling size** | |  |  |  |  |  |  |  |  |  |
| <400 | | 49 | 84 | 1.26(0.95-1.69) | 84.409 | - | 518/32961 | Ref. | Ref. | Ref. |
| ≥400 | | 48 | 65 | 0.80(0.62-1.03) | 90.547 | - | 848/81761 | -0.26(-0.63-0.1) | 0.18 | 0.77 (0.53-1.1) |
| **Language** | |  |  |  |  |  |  |  |  |  |
| Chinese | | 96 | 148 | 0.98(0.80-1.21) | 89.817 | - | 1342/114412 | Ref. | Ref. | Ref. |
| English | | 1 | 1 | 7.74(5.24-11.29) | 0.000 | - | 24/310 | 3.61(1.77-5.45) | 0.93 | 37.02 (5.9-232.5) |
| Quality score (continuous) | | 97 | 149 | 0.96 (0.73-1.25) | 94.810 | - | 1366/114722 | -0.05(-0.69-0.59) | 0.32 | 0.95 (0.5-1.8) |
| Study period (continuous) | | 97 | 149 | 0.96 (0.73-1.25) | 94.810 | - | 1366/114722 | -0.2(-0.31--0.1) | 0.05 | 0.82 (0.73-0.91)* |

**Reference:**

1. Wei C, Wu W, Xiong Y, et al. [Study on infection rate of sexually transmitted disease s of female commercial workers]. Modern Preventive Medicine 2007(19):3651-52

2. Liu S, Li J, Hao C, et al. [A cross-sectional investigation on AIDS risk factors for female commercial workers in public bath]. Morden Preventive Medicine 2007;**34**(16):3155-56+59

3. Zhang Z, Wu X. [Survey analysis of the STI prevalence in Sanming of Fujian Province]. China J Lepr Skin Dis 2009;**25**(10):721

4. Zheng B, Yao J, Chen J, et al. [STI prevalence of female sex workers from the entertainment venues]. China J Lepr Skin Dis 2003;**19**(6):633

5. Du Y, Yang H, Jian P, et al. [Study on the effectiveness and sustainability of implementing 100% condom use programme in entertainment establishments]. Jiangsu J Prev Med 2004;**15**(3):31-33

6. Yan H, Xu X, Guan W, et al. [A Survey on high risk behavior and main in sexually transmitted diseases among prostitutes in Jiangsu]. Chin J AIDS STD 2004;**10**(01):36-37+52

7. Yang P, Su X, Yin Y, et al. [Investigation of STD intervention modes among female sex workers in China]. China J AIDS/STD 2005;**11**(3):195-97

8. Xu L, Gu W, Zhao G. [Study on medical outreach services in femal sex workers of a sauna bath place in Shanghai]. Chin J Public Health 2006;**22**(8):962-64

9. Lin YQ, Chen LH, Wang RQ, et al. [A study on STD in high-risk populations in Wenzhou city of Zhejiang province]. Zhejiang Journal of Preventive Medicine 2005;**17**(4):20

10. Wang FJ, Jin GS, Qian JR, et al. [Investigation on sexually transmitted infections among 608 female sex workers]. Zhejiang Journal of Preventive Medicine 2008;**20**(5):33, 41

11. Zhang Y, Zhou J, Xie J, et al. [Investigation on knowledge, awareness and risk bebaviour related to HIV/AIDS among female sex workers based on lowclass establishments in some areas of China]. Chin J AIDS STD 2005;**11**(06):415-17

12. Ni CM, Wu HP, Chen MF. [Surveillance on STD infection of some sex workers and their clients in Haiyan county during 2004-2008]. Chinese Journal of Health Laboratory Technology 2011;**21**(9):2269-70

13. Zeng Y, Fang F, Wang H, et al. [Prevalent analysis of Sexua lly Transm itted D iseases in 892 Prostitutes andWhoremasters in Jinshan D istr ict of Shangha i]. Chin J Derm Venereol 2009;**23**(04):228-30

14. Yan H, Chen G, Cao G, et al. [Analysis of baseline survey on expanding behavior interventions programme at entertainment establishments in Jiangsu province]. Jiangsu J Prev Med 2007;**18**(1):12-15

15. Xu HQ, Wang JW. [Analysis on HIV/AIDS intervention for commercial sex workers at public venues in Xiuzhou District, Zhejiang Province]. Zhejiang Journal of Preventive Medicine 2008;**20**(7):72-73

16. Shu CH, LI LM. [Detection of Chlamydia trachomatis among 225 Illicit Prostitutes]. Jiangsu Health Care 2011;**13**(6):18

17. Hu J, Liu L, Ge H, et al. [Investigation on knowledge, behavior about STD/AIDS HIV infection situation of 256 female commercial sex workers ]. Modern Preventive Medicine 2007;**34**(13):2521-23

18. Yu X. [Survey on the knowledge, attitude, and behavior related to HIV/AIDS among prostitutes in the entertainment establishments in Haimen city]. Journal of Public Health and Preventive Medicine 2007;**18**(2):104-05

19. Zhu F, Ji K, Li Z, et al. [Evaluation on the Effect fo AIDS/STD Related Knowledge Training and Behavior Intervention among Femal Sex Workers in Entertainment Places]. Prev Med Trib 2009;**v.15**(05):420-22

20. Wang W, Xue L, Xia L, et al. [Survey of 297 sex workers and the infectious status of STD and HIV/AIDS]. China Tropical Medicine 2008;**8**(05):827-28

21. Yang JY, Fan XW. [Status of STDs Epedemic Among Female Sexual Workers in Chong'an District in Wuxi] Journal of Preventive Medicine Information 2011;**27**(11):905-07

22. Chen JQ, Chen WY. [A survey on HIV/STD infection among 168 female sex workers] Zhejiang Preventive Medicine 2009;**21**(11):25-26

23. Zhao YQ, Zhao XP, Cao XP, et al. [AIDS high-risk behaviour and STD infection rates: Investigation on 396 demimondaines]. Shanghai Journal of Preventive Medicine 2010;**22**(3):137, 41

24. Wang F, Chu Y, Wang L, et al. [Serological survey and investigation on AIDS knowledge and behavior among CSW in Huaiyuan county]. Anhui J Prev Med 2010;**v.16**(03):190-91+202

25. Li XY, Zhang YL, Zhang SP, et al. [Infection of sexually transmitted diseases among 98 female sex workers]. Chinese Journal of Nosocomiology 2011;**21**(11):2274-75

26. Zhang QQ, Huan XP, Yang HT, et al. [Analysis of STDs infection and related high-risk factors among FSWs in Jiangsu Province]. Acta Universitatis Medicinalis Nanjing(Natural Science) 2012;**32**(4):473-78

27. Wei Z, Sun L. [Investigation on behavior and infection of STDs and AIDS among female sex workers at entertainment sites in Yangzhou city]. Prev Med Trib 2010;**16**(12):1123-25

28. Tang ZL, Li XF, Dong XP, et al. [Infection of HIV/STDs through sexual behavior among female sex workers during menstruation]. Chinese Journal of Public Health 2011;**27**(12):1510-12

29. Li DL, Ding HF, Luo FJ. [Epidemiological analysis of sexual transmitted infection in 2517 FSWs and their male clients]. Chinese Journal of AIDS & STD 2006;**12**(4):344-46

30. Zhao YR. [Analysis on STD laboratory screening test among female sex workers and clients]. Chinese Journal of AIDS and STD 2003;**9**(5):310-14

31. Li Y, Wang KL, Tong X, et al. [Analysis of sentinel monitoring of AIDS among high risk population in Heilongjiang Province in 2010]. Chinese Primary Health Care 2011;**25**(5):59-61

32. Lu H, Zhang L, Li S. [A study of HIV/STD knowledge and sexual behaviours among drug users and female sex workers in Urumqi]. Endemic Diseases Bulletin 2003;**18**(04):52-53

33. Xi CH, Liu XF, Li XM, et al. [Investigation on HIV, STD, HBV, HCV prevalence and behavior among female sex workers in entertainment establishments in Lanzhou City of Gansu Province]. Chinese Journal of AIDS and STD 2004;**10**(1):59-60

34. Wei X, Ye Y, Wang H, et al. [A study of HIV/STD testing and KABP among entertainment-based female sex workers in Shihezi prefecture, China]. Journal of Preventive Medicine Information 2005;**21**(3):264-67

35. Li F, Zhang F, Li R, et al. [Study of HIV risk factors among commercial sex workers in four cities of Xinjiang]. Chinese Journal of AIDS and STD 2006;**12**(1):27-29

36. Wang H, Zhang L. [STD/HIV testing results analysis among 366 female sex workers]. Endemic Diseases Bulletin 2006;**21**(04):28-29

37. Song Y, Muheta, Aziguli, et al. [A survey of sexually transmitted diseases among entertainment-based female sex workers in Turpan City, Xinjiang, in 2006]. Endemic Diseases Bulletin 2006;**21**(06):36-37

38. Yang SP, Zhang ZJ, Dong YH, et al. [Evaluation on HIV/STD intervention for female sex workers in Hami City, Xinjiang Uyghur Autonomous Region]. Chinese Journal of AIDS and STD 2008;**14**(05):513-15

39. Abudureyimu AYS, Amuti DLK. [Evaluation of AIDS intervention among road-sided prostitutes in Turpan City]. Xinjiang Medical Journal 2011;**41**(3):58-59

40. Gao W, Li Z, Yan H, et al. Preventive measures against sexually transmitted infections among female sex workers in Lanzhou, China. Scand J Infect Dis 2012;**44**(5):374-80 doi: 10.3109/00365548.2011.644250[published Online First: Epub Date]|.

41. Zhang CJ, Xu SL, Ding J. [Analysis on STD Examination result of 96 sexual criminals]. Henan Journal of Preventive Medicine 2001;**12**(6):347

42. Zhongdan C, Schilling RF, Shanbo W, et al. The 100% Condom Use Program: a demonstration in Wuhan, China. Eval Program Plann 2008;**31**(1):10-21

43. HU B, Lin J, Feng Y, et al. [Analysis of results in popularization the condoms in entertainment places for prevention of STD]. Chin Tropical medicine 2004;**4**(4):658-59

44. Liang L, Lin J, Yang J. [Survey of infectious status of sexually transmitted diseases in 421 girls working in public and entertainment places in Danzhou City]. China tropical medicine 2003(06):856-57

45. He Q, Lin J, Zeng X, et al. [Analysis on STD and HIV infection situation in 417 female sex workers]. Chin J Public Health 2003;**19**(9):1096-97

46. Luo J. [Investigation on the knowledge, attitude, behaviour and infection situation of STD/AIDS among female sex workers]. Modern Preventive Medicine 2005;**32**(06):644-45

47. Peng S, Hong F, LI Y, et al. [A survey of Chlamydia Trachoma injection status among 110 female sex workers]. Modern Preventive Medicine 2004(03):413-14

48. Tang G, Lan S, Wu Z, et al. [Evaluation of Effect of 100% Condom Usage Promotional Project in Prevention of STD in Hecheng District]. Practical Prevent ive M edicine 2007(03):920-21

49. Xiong C, Li S, Chen X, et al. [Effectiveness evaluation of condom distribution in prevention of HIV/STD in entertainment establishment in Wuling district, Hunan province]. Chin Prev Med 2006;**7**(04):316-17

50. Hong Y, Li X, Yang H, et al. HIV/AIDS-related sexual risks and migratory status among female sex workers in a rural Chinese county. AIDS care 2009;**21**(2):212-20

51. Wang B, Li X, Stanton B, et al. Vaginal douching, condom use, and sexually transmitted infections among Chinese female sex workers. Sex Transm Dis 2005;**32**(11):696-702

52. Zhou Y, Liu W, Dong B, et al. [Analysis on HIV 2related knowledge , attitude , condom using and STD prevalence among commercial sex workers in a township]. Chin J Dis Cont rol Prev 2007;**11**(5):488-90

53. Jiang M, Wu JH, Huang JG, et al. [STD/AIDS knowledge, attitude, behaviour and infection status among prostitutes in entertainment sites]. Theory and practice of Chinese Medicine 2005;**15**(1):152-53

54. Yang B, Xu J, Wang X, et al. [Investigation on high risk behavior and chlamydia trachomatis infection in prostitutes]. J of Pub Health and Prev Med 2006(03):31-33

55. Zhang WS, Qiu XQ, Wen YM. [Survery of sexually transmitted diseases and HIV infected condition in Xiangfan city]. Journal of Public Health and Preventive Medicine 2006;**17**(4):67-68

56. Wu M, Huang T, Liang G, et al. [STD/HIV knowledge and infection status survey among entertainment-based female sex workers in Beihai City]. Guangxi Medical Journal 2006;**28**(4):510-12

57. Zhang J. [Investigation on STD clinical epidemics among 103 high-risk females from He Zhou]. Guangxi Medical Journal 2008;**30**(9):2

58. Li C, Ou Y, Luo Y, et al. [Effectiveness evaluation of behavioural interventions for HIV/STDs at-risk populations in low-level entertainment venus in Shaoguan region, China]. Southern China Journal of Dermato-Venereology 2007;**14**(04):242-44

59. Li Y, Detels R, Lin P, et al. Difference in risk behaviors and STD prevalence between street-based and establishment-based FSWs in Guangdong Province, China. AIDS and behavior 2012;**16**(4):943-51 doi: 10.1007/s10461-011-0102-0[published Online First: Epub Date]|.

60. Li Y, Lin P, Detels R, et al. [Prevalence of HIV infection and sexually transmitted disease and associated risk factors among female sex workers in Guangdong province]. Disease Surveillance 2009;**24**(08):599-602

61. Wu Z, Zhu X, Huang S, et al. [Prevalence of Sexually Transmitted Diseases and AIDS in Massagers in Jiangmen City]. Journal of Tropical Medicine 2008;**8**(01):80-81

62. Zhu BY, Bu J, Huang PY, et al. Epidemiology of sexually transmitted infections, HIV, and related high-risk behaviors among female sex workers in Guangxi Autonomous Region, China. Japanese journal of infectious diseases 2012;**65**(1):75-8

63. Jiang, M, Wu, J.H, Huang, J.Q, et al. [Survey of STI/ AIDS-related knowledge, attitude and behaviors and infection rates of sex workers in entertainment places in Jingzhou City]. Chinese Journal of Disease Control & Prevention 2012;**16**(2):175-77

64. Yi QM. [A study on sexual behaviour and sexually transmitted infection among 300 female at entertainment sites]. Practical Prevetive Medicine 2009;**16**(4):1295-96

65. Zhao Y, Li M, Li Y, et al. [The status of sexually tmsmitted infections in the female sex workers at the entertainment venues in Panyu district. International Medicine & Health Guidance News 2010;**16**(18):2212-14

66. Xiang S, Peng J, Tang J, et al. [HIV knowledge, attitude and behavioural survey among female sex workers in Hongjiang city]. Practical prevetive Medicine 2010;**17**(3):596-97

67. Zhong J, Lin J, Hu YM, et al. [HIV/STD infection and risk behaviors among commercial sex workers at various places in Wuzhou City, Guangxi Zhuang Autonomous Region]. Chinese Journal of Health Education 2011;**27**(3):177-80

68. Zhong J, Lin J, Hu Y, et al. [HIV/STI infection and risk behaviors among 406 commercial sex workers in Wuzhou City, Guangxi PRC]. Chinese Journal of Drug Abuse Prevention and Treatment 2010;**v.16**(06):318-21

69. Zhao JZ, Ren SH, Wan Y, et al. [Research on AIDS/Infectious Diseases of Genital Tract and Related Behavior of Female Sex Workers in Low-grade Areas]. Chinese Journal of Social Medicine 2011;**28**(5):326-28

70. Zhu L, Qiu XS, Xie AQ, et al. [Analysis of AIDS Sentinel Surveillance among prostitutes in Xiangyang in 2010]. Journal of Public Health and Preventive Medicine 2011;**22**(3):92-93

71. Chen Z, Huang B, Huang S. [The evaluation on STD/AIDS intervention among female sex workers of Jiang Men] South China J Prev Med 2007;**33**(04):29-30+33

72. Yang P, Wang Q, Zhong M. [Factors associated with STD infection among female sex workers]. China J Lepr Skin Dis 2006(02):149-50

73. Wu Y, Liao H, Zhou W, et al. [Surveillance and Intervention Study of Sexual Transmitted Disease in Commercial Female Sex Workers]. surveillance of diseases 2002;**17**(10):365-67

74. Wang Q, Yang P, Wan S, et al. [Mass treatment service integrated with comprehensive intervention for female sex workers in Sichuan Province]. Chin J AIDS STD 2005;**11**(6):438-41

75. Wu Y, Kuang F, Zhang J, et al. [HIV/STD interventions analysis among female sex workers]. Chinese Journal of AIDS and STD 2007;**13**(01):69-70

76. Dai XQ, Wu Y, Zhang J. [Investigation on genital Molluscum Contaqiosum among female sex workers]. Chongqing Medical Journal 2007;**36**(3):253-54

77. Tan X, Yi H, Wang Z, et al. [An analysis of knowledge, attitude and the feature of high risk behavior on AIDS among 266 unlicensed prostitutes ]. Modern Preventive Medicine 2007;**34**(02):253-55+62

78. Ding X, Yi H, Jiang X, et al. [Analysis of status of AIDS related knowledge, attitude and risk behavior among 519 female sex workers in Chongqing]. Chin J AIDS STD 2006;**12**(04):347-49

79. Wang HB, Smith K, Brown KS, et al. Prevalence, incidence, and persistence of syphilis infection in female sex workers in a Chinese province. Epidemiol Infect 2010:1-9

80. Xu J, Brown K, Ding G, et al. Factors associated with HIV testing history and HIV-test result follow-up among female sex workers in two cities in Yunnan, China. Sex Transm Dis 2011;**38**(2):89-95

81. Wang H, Reilly KH, Brown K, et al. HIV incidence and associated risk factors among female sex workers in a high HIV-prevalence area of China. Sex Transm Dis 2012;**39**(11):835-41 doi: 10.1097/OLQ.0b013e318266b241[published Online First: Epub Date]|.

82. Li Q, Xu J, Wang H, et al. [Prospective epidemiological study of factors correlated with seeking HIV post testing counseling service among female sex workers in Kaiyuan city]. Chin J AIDS STD 2010;**16**(03):295-97

83. Xu JJ, Wang N, Lu L, et al. HIV and STIs in clients and female sex workers in mining regions of Gejiu City, China. Sex Transm Dis 2008;**35**(6):558-65

84. Wang H, Chen RY, Ding G, et al. Prevalence and predictors of HIV infection among female sex workers in Kaiyuan City, Yunnan Province, China. Int J Infect Dis 2009;**13**(2):162-9

85. Wang H, Brown KS, Wang G, et al. Knowledge of HIV seropositivity is a predictor for initiation of illicit drug use: incidence of drug use initiation among female sex workers in a high HIV-prevalence area of China. Drug Alcohol Depend 2011;**117**(2-3):226-32 doi: 10.1016/j.drugalcdep.2011.02.006[published Online First: Epub Date]|.

86. Wang H, Brown KS, Wang G, et al. Knowledge of HIV seropositivity is a predictor for initiation of illicit drug use: Incidence of drug use initiation among female sex workers in a high HIV-prevalence area of China. Drug and Alcohol Dependence 2011;**117**(2-3):226-32

87. Luo X. [Knowledge, behavior about STD/AIDS and HIV infection situation of female commercial sex workers in Yibin city]. J Prev Med Inf 2008;**24**(07):566-68

88. Li Q, Xu J, Wang W, et al. [Survey of high risk behaviors and HIV/STD infection among FSWs from different venues of Kaiyuan city]. Chin J AIDS STD 2009;**15**(02):164-66

89. Wang G, Ding G, Wang H, et al. [Two cross-sectional studies of HIV/STD infection status among entertainment-based female sex workers in Yunnan]. Soft Science of Health 2008;**161**(5):343-45

90. Gao L, Che Z, Lu Y. [A cross-sectional study on STDs/AIDS and hiv of 270 female sex workers]. J Dematology and Venereology 2008;**30**(03):39-42

91. Xu JJ, Smith MK, Chu J, et al. Dynamics of the HIV epidemic in southern China: sexual and drug-using behaviours among female sex workers and male clients in Yunnan. Int J STD AIDS 2012;**23**(9):670-5 doi: 10.1258/ijsa.2009.009128[published Online First: Epub Date]|.

92. Yao Y, Yang F, Chu J, et al. Associations between drug use and risk behaviours for HIV and sexually transmitted infections among female sex workers in Yunnan, China. Int J STD AIDS 2012;**23**(10):698-703 doi: 10.1258/ijsa.2012.011346[published Online First: Epub Date]|.

93. Jin X, Chan S, Ding G, et al. Prevalence and risk behaviours for Chlamydia trachomatis and Neisseria gonorrhoeae infection among female sex workers in an HIV/AIDS high-risk area. International journal of STD & AIDS 2011;**22**(2):80-4 doi: 10.1258/ijsa.2010.010289[published Online First: Epub Date]|.

94. Yan WZ, Zheng KQ, Feng DL, et al. [Investigation on status of STDs and AIDS among female sex workers in JingHong County of Yuinan in 2009]. Journal of Dermatology and Venereology 2011;**33**(1):51+54

95. Zhang XD, Temmerman M, Li Y, et al. Vulnerabilities, health needs and predictors of high-risk sexual behaviour among female adolescent sex workers in Kunming, China. Sex Transm Infect 2013;**89**(3):237-44 doi: 10.1136/sextrans-2012-050690[published Online First: Epub Date]|.

96. Shao ZP, Zhang ZY. [STD /AIDS Detection and Condom Use of Female Sex Workers]. Journal of Preventive Medicine Information 2012;**28**(8):618-20

97. Liao CQ, Zhong YX. [Suvey on AIDS Knowledge and Safe Sex among Femal Sex Workers in Hongkou District of Shanghai]. Health Education and Health Promotion 2011;**6**(3):197-98

98. Guo YY, Zhang GX, Wang HQ. [Seroepidemiological study on syphilis, gonorrhoeae and HIV infection among female sex workers in Xiaoshan District in Zhejiang Province during 2001-2007]. Chinese Journal of Rural Medicine and Pharmacy 2010;**17**(5):63-64

99. Li L, Ding JP, Du YP, et al. [Analysis on baseline study on Jiangsu / WHO 100% condom use program to prevent STD & AIDS]. Jiangsu Preventive Medicine 2003;**14**(3):19-21

100. Qi H. [Study of STD/AIDS detection and condom utilization conditions of 207 unlicensed prostitutes in Yancheng city]. Disease Surveillance 2007;**22**(01):31-32

101. Song S, Chen S, Ding J, et al. [Analysis of the status of STD/AIDS detection and condom utilization of 272 CSW in Hangzhou ]. Modern Preventive Medicine 2008;**35**(18):3625-26

102. Zhang Q, Jiang X, Jiang B, et al. [Analysis on High-risk Behaviors Related to AIDS of CSW in Nanchong City]. J Prev Med Inf 2006;**22**(1):28-32

103. Wang J, Wang B, Dong GD, et al. [Analysis on STD/HIV infection and related risk behaviors among commercial sex workers in entertainment sites in Jiangsu Province]. Southern China Journal of Dermato-Venereology 2008;**15**(6):363-65

104. Chen JH, Fang JA, Zhang YF, et al. [Investigation on HIV/STD infection and related risk behaviors among roadside female sex workers]. Modern Preventive Medicine 2009;**21**(3):280-81

105. Wu RH, Zhang Y, Shu YY, et al. [An analysis of gonorrhea and syphilis conditions among 1142 femal sex workers and their clients in one district of Shanghai]. Chinese Journal of AIDS & STD 2012;**18**(5):325-26

106. Shao M, Shen J, Zhao X, et al. [Investigation on AIDS and Venereal disease infection among CSW crowd in Suzhou Area]. Occup and Health 2010;**26**(8):886-87

107. Zhang QQ, Huan XP, Yin YP, et al. [Incidence rates of sexually transmitted infection and the characteristic analysis of female sex workers failing in follow-up in cohort study]. Acta Universitatis Medicinalis Anhui 2012;**47**(9):1050-54

108. Li JE. [HIV /STD infection station of FSW in entertainment places and evaluation the intervention efficacy of health education]. Chinese Journal of Pest Control 2012;**28**(7):739-41

109. Zhang XY, Hao Y, Ji MS, et al. [An epidemiological study on HIV /STD infection in female sex workers in Baotou city]. The Chinese Journal of Human Sexuality 2012;**21**(10):94-95+63

110. Bing L, Feng S, Jun G, et al. [Investigation and Evaluation of Intervention effect for STD infection in women working in public places for entertainment]. Practical Preventive Medicine 2002;**9**(5)

111. Wei X, Wan C, Wang H. [Investigation on STD epidemics among females sex workers from Shi He Zi, Xinjiang]. China J of PHM 2002;**18**(5):389-90

112. Sun L, Sun J. [Study on STD/AIDS prevention on female sex workers from Kai Feng]. Henan J Prev Med 2003;**14**(4):223-24

113. Sun Jie SL, Zhou Dong Sheng. [Survey on STI epdimics among female sex workers in Kai Feng]. Henan J Prev Med 2003;**14**(2):89-90

114. Peng S, Zhang R, Lai Y, et al. [The comparison of neisseria gonorrhoeae detection of excretion from vagina and cervix among female sex workers]. Modern Preventive Medicine 2004:254-55

115. Liang B, Ye X, Yang A, et al. [Analysis Of Epidemiological Trend and Infextion Status of STD/AIDS Among Sex Workers During 2002 to 2005 in Guangzhou]. Modern Preventive Medicine 2008(08):1401-02+07

116. Fan XJ, Li XP, Du DC, et al. [Evaluation of behavioural intervention for female sex workers in entertainment sites in Shimen County in Hunan Province]. Chinese Journal of AIDS and STD 2007;**13**(S1):26-27

117. Li M, Gan Z, Wang Y, et al. [Evaluation of behavioral intervention of AIDS among the commercial sex workers in the public entertainment places]. Chin J Dis Control Prev 2005;**9**(4):306-08

118. Ren Y. [Study on STD/AIDS epidemics among detained population in Zhu Hai]. Chinese Journal of Drug Abuse Prevention and Treatment 2007(02):113-14

119. Sun C, Chen X, Zhu M, et al. [Effect assessment on the intervention to the high-risk behavior of female sex workers in entertainment places]. Chinese primary health care 2009;**23**(01):30-31

120. Zhang SJ. [Study on HIV/AIDS epidemics in Cong Zuo during 1996 to 2007]. Chinese Journal of Internal Medicine 2008;**v.3**(06):932-35

121. Li X, Guo D, Hu Y, et al. [Cross Sectional Study on Knowledge of AIDS and Sexual Behavior Among Female Sexual W orkers in Public Places]. China and Foreign Medical Journal 2008:12-14

122. Liang HX, Deng X, Tang GZ, et al. [Investigation of AIDS among Female Sex Workers in A District of Shiyan City during 2009-2010]. Occupation and Health 2011;**27**(20):2338-39

123. WeiPing W, ZhongXian S, Pingcheng H, et al. [Invest igation on STDs Infection Among Female Employees in Entertainment Establishments of Zhangjiajie] Practical Preventive Medicine 2008;**15**(1):243-46

124. Chen S, Cao X, Liang Z. [Investigation on the knowledge, attitude and behaviour of AIDS among female sex workers from entertainment venue in Fo Shan]. Chinese Journal Of Practice Medicine 2005:4

125. Wu J. [STD infection status among 103 entertainment female sex workers]. Chin J STD/AIDS Prev Cont 2002;**8**(4):203

126. Li Y, Wen Y, Hu Z, et al. [Integrated AIDS Behavioral Intervention Among Female Sex Workers in Gejiu City]. Journal of Kunming Medical University 2009;**v.30**(09):113-17+21

127. Ruan Y, Cao X, Qian HZ, et al. Syphilis among female sex workers in southwestern China: potential for HIV transmission. Sex Transm Dis 2006;**33**(12):719-23

128. Cao XT, Ruan YH, Jiang ZQ, et al. [Study on sexually transmitted diseases and related risk factors among female sex workers who have regular partners]. Chinese Journal of Preventive Medicine 2006;**40**(2):144

129. Yang X, He Z, Han C, et al. [Study on prevalence of sexually transmitted infections among female sex workers in part areas of Yunnan]. Soft Science of Health 2009;**v.23;No.164**(02):229-31, 33

130. Pan H. [STD/HIV surveillance and health education among entertainment workers]. Medical forums in basic 2011;**15**(04):186-87

131. Zhang YH, Bao YG, Li CM, et al. [Study of HIV/ syphilis infection status of commercial sex workers in 15 cities of China]. China Preventive Medicine 2011;**12**(5):387-90

132. Chen XS, Liang GJ, Wang QQ, et al. HIV prevalence varies between female sex workers from different types of venues in southern China. Sex Transm Dis 2012;**39**(11):868-70 doi: 10.1097/OLQ.0b013e318264c3ba[published Online First: Epub Date]|.

133. Zhang H, Chen CG, Lin FH, et al. [Analysis of Comprehensive Surveillance Results of HIV /AIDS-related High- risk Groups in Fuzhou City in 2010]. Occupation and Health 2011;**27**(21):2406-09

134. Qin QR, Zhan SW, Cao DZ, et al. [Needs and recognition of HIV voluntary counseling and testing among female sex workers in low-class establishments of Maanshan]. Chinese Rural Health Service Administration 2011;**31**(4):391-92

135. Zhang CX, Shan RQ, Zhou ZM, et al. [Investigation on the demographics, HIV and sphilis infection of Female Sex Workers]. Modern Practical Medicine 2011;**23**(10):1169-70

136. Li HX, Tang CG. [Hepatitis B,AIDS,Syphilis simultaneous detection of program analysis and discussion]. Modern Preventive Medicine 2011;**38**(20):4244-45

137. Xie HY. [Investigation on HIV and syphilis infection among 1996 female sex workers in Quanzhou City, Fujian Province]. Strait Journal of Preventive Medicine 2004;**10**(6):44-45

138. Hua Y. [Investigation on syphilis prevalence among 832 female sex workers]. Jiangsu Health Care 2006;**8**(05):12

139. Chen Y, Cai X, You T, et al. [Analysis on risk behaviors and HIV/syphilis infection among female sex workers(FSWS) in detention home of Longyan city from 2001 to 2007]. Prev Med Trib 2009;**15**(09):827-28

140. Wang BF, Chen QJ, Zhang YH, et al. [Analysis on Behaviour Charaeteristics of Prostitute and Epidemiology of HIV and Syphilis]. Disease Surveillance 2004;**19**(8):296-97

141. Xie Y, Chen JH, Wang ZH. [A survey on the characteristics and HIV, syphils infection among prostitutes in reeducation center in Zhangzhou City, Fujian Province]. Strait Journal of Preventive Medicine 2006;**12**(4):38-39

142. Li X, Zhang B, Liu M, et al. [Survey on the behavioral characteristics related to HIV/AIDS and the sero prevalence of sexually transmitted disease(STD) among 466 female sex workers(FSW) in Qingdao]. Chin J AIDS STD 2006;**12**(1):16-18

143. Wang H, Tan L, Sun B, et al. [Cross infectious epidemiological study of HSVII, HIV, Syphilis, HBV and HCV in female sexual servers]. J Clin Transfus Lab Med 2005;**7**(3):187-89

144. Zheng H, Ding Y, Fu QF, et al. [Effect of Intervention to Commercial Sex Workers in Comprehensive Prevention Demonstrative Area]. Jiangsu Health Care 2012;**14**(1):8-10

145. Yang M, Cai X, Li S, et al. [Intervention modes and cross-sectional survey on AIDS behavior among femal sex workers in entertaining establishing in Shanghai]. Chin J Epidemiol 2009;**30**(4):419-20

146. Yu WenXiang QY, Wang HaiYan, Sun Kaiyou. [Surveilance report on HIV/STD epidemics in Yan Cheng, 2005] Jiangsu Prev Med 2006;**17**(4):16-17

147. Wang Y, He Y, Ma L, et al. [Prevalence of HIV and Syphilis and Knowledge of HIV/AIDS among female workers of entertainment places in Tongling city]. Anhui J Prev Med 2007;**13**(04):248-49+52

148. Hong H, Xu GZ, Zhang DD. Long-term follow-up of a comprehensive HIV and sexually transmitted infection prevention program for female sex workers in Ningbo, China. Int J Gynaecol Obstet 2010;**111**(2):180-1

149. Huang Y, He YX, Wang Y, et al. [Evaluation of AIDS intervention program based on sexually transmitted disease clinics among female sex workers]. Chinese Journal of AIDS & STD 2012;**18**(8):543-46

150. Sun Z, Xue F, Lin S, et al. [Survey of knowledge and behavior associated with AIDS in prostitutes in educational house]. China Tropical Medicine 2008;**8**(03):511-12

151. Liao M, Jiang Z, Zhang X, et al. Syphilis and methamphetamine use among female sex workers in Shandong Province, China. Sex Transm Dis 2011;**38**(1):57-62

152. Kang D, Liao M, Jiang Z, et al. Commercial sex venues, syphilis and methamphetamine use among female sex workers. AIDS care 2011;**23 Suppl 1**:26-36 doi: 10.1080/09540121.2011.560930[published Online First: Epub Date]|.

153. Luo Y, Chen S, Xu K, et al. [Survey of STD/AIDS-related knowledge, behaviors and infection rates of sex workers in entertainment places in Hangzhou]. Disease Surveillance 2008;**23**(10):607-09

154. Wang F, Chen X, Su B, et al. [Analysis of the results of the comprehensive HIV/AIDS surveillance among sex workers in Anhui]. Anhui J Prev Med 2009;**15**(06):407-08

155. Tang X, Li Y, Li LY, et al. [The assessment of HIV/AIDS structural intervention program among female sex workers in small entertainment settings in Hongkou District of Shanghai]. Chinese Journal of AIDS & STD 2012;**18**(10):669-72

156. Liao M, Nie X, Pan R, et al. Consistently low prevalence of syphilis among female sex workers in Jinan, China: findings from two consecutive respondent driven sampling surveys. PloS one 2012;**7**(4):e34085 doi: 10.1371/journal.pone.0034085[published Online First: Epub Date]|.

157. Xue F. [Survey of AIDS-related knowledge and infection rates of HIV, HBsAg and Syphlis among female sex workers in entertainment places in Lucheng district of Wenzhou city]. Chinese Journal of Health Laboratory Technology 2009;**19**(7):1649-51

158. Tang X, Wu P, Li Y, et al. [A study on AIDS related knowledge and behavioral characteristics among mini-type entertainment venues based female sex workers in Hongkou district of Shanghai]. Chin Prev Med 2010;**11**(02):162-65

159. Luo XY, Shi RQ. [Investigation on HIV and syphilis infection among female sex workers in Pinghu City, Zhejiang Province]. Zhejiang Preventive Medicine 2010;**22**(7):25-26

160. Yang LQ, Fu HP. [Study on the HIV intervention effects to FSWs from Entertainment venues]. Zhejiang Journal of Preventive Medicine 2012;**24**(12):24-26

161. Chen ZH, Xie JR, He J, et al. [Study on HIV/AIDS detection and related knowledege among female sex workers in Zunji]. Zhejiang Journal of Preventive Medicine 2012;**24**(3):81-82

162. Xi SJ, He YF, Zhou XH. [Analysis on the Result of AIDS Test Intervention in CSW, MSN, IDU Population in Xiacheng District]. Zhejiang Journal of Preventive Medicine 2010;**22**(9):29-30

163. JingHua W. [Survey on HIV, HCV, HBV and syphilis prevalence among high-risk group in a Chinese city]. Journal of Community Medicine 2010;**v.8**(19):22-23

164. Wang B, Wang J. [A study on HIV/AIDS and syphilis surveillance among 400 commercial female sex workers]. Journal of Dematology and Venereology 2010;**32**(4):47-48

165. Chen GS, Wu HS, Yao ZM. [Investigation on HIV/AIDS related knowledge, behavior and infection among commercial female sex workers in one county]. Anhui Journal of Prevent Medicine 2010;**16**(5):418-19

166. Jin HJ, Fang JA, Liu JM, et al. [Sentinel surveillance analysis on female sex workers in Zhenhai County in Ningbo City]. Shanghai Journal of Preventive Medicine 2010;**22**(8):405-06

167. Chen XS, Wang QQ, Yin YP, et al. Prevalence of syphilis infection in different tiers of female sex workers in China: implications for surveillance and interventions. BMC Infect Dis 2012;**12**:84 doi: 10.1186/1471-2334-12-84[published Online First: Epub Date]|.

168. Ma JH, Zhang JB, Mou YX. [A follow-up study on HIV and syphilis infection among prostitutes in Yizheng city of Jiangsu province]. Jiangsu Journal of Preventive Medicine 2012;**23**(06):51-52

169. Chen SX, Zhang ML, Han XM. [Survey on AIDS Related Knowledge, Behavior and the HIV Infection Status Among Commercial Sex Workers in Gaomi City]. Preventive Medicine Tribune 2011;**17**(12):1119-20, 23

170. Chen CC, Lin H, Zhang H. [AIDS knowledge Levels and Behavior Characteristics of 225 Female Sex Workers in Entertainment Places of Fuzhou City]. Occupation and Health 2011;**27**(2):170-72

171. Chen MF, Xu FF, Qiu LX. [Comprehensive survey of female sex workers of HIV]. Chinese Journal of Health Laboratory Technology 2011;**21**(5):1266-67

172. Qian ZH, Wang J, Fan XQ. [HIV/AIDS sentinel surveillence results among prostitutes in Suzhou city]. Jiangsu Journal of Preventive Medicine 2012;**23**(1):30-31

173. Gan WH, Zhu JM, Jiang CH, et al. [Analysis of AIDS sentinel surveillance among the female sex workers in Jinshan district of Shanghai from 2009-2011]. Chinese Journal of AIDS & STD 2012;**18**(7):457-58+74

174. Miao XL, Cheng H, Zhang X, et al. [Analysis on HIV /AIDS Sentinel Surveillance in Wuxi City in 2010]. Occupation and Health 2011;**27**(22):2599-601

175. Ma P, Chen DL, Yuan JM. [Survey on syphilis prevalence and impact factors among femal sex workers in Nantong,2010]. Chinese Journal of AIDS & STD 2011;**17**(5):561-62+76

176. Zhang HF, Wang GH, ZHao Z, et al. [Analysis on the prevalence factors of syphilis among the ordinary women and female sex workers in Tongxiang city]. Chinese Journal of Health Laboratory Technology 2012;**22**(10):2483-85

177. Tang Y, Sheng YH, Wang T, et al. [Surveillance of AIDS and syphilis in different high-risk groups in Pudong New District of Shanghai,2010-2011]. Chinese Journal of AIDS & STD 2012;**18**(8):540-42

178. Ma P, Chen DL, Yuan JM. [Survey on syphilis prevalence and its influence factors among three different groups in Nantong City]. Jiangsu Journal of Preventive Medicine 2012;**23**(2):34-36

179. Liu LL, Yin J. [Analysis of sentinel monitoring on FSWs in Jiangyen City in 2010]. Chinese Primary Health Care 2011;**25**(8):96-97

180. Jiang J, Wang HB, Fang WM, et al. [Survey of AIDS prevalence in female sex workers detained in a correctional facility in Ningbo, Zhejiang]. Disease Surveillance 2012;**27**(08):634-36

181. Ye YingDan SF, Wen Jun. [Surveilance results of syphilis and HIV prevalence of female sex workers in Chang Shu]. Shanghai Journal of Prevenive Medicine 2011;**23**(8):377-79

182. Ye YD, Shi FL, Wen J. [Monitoring of Syphilis and HIV Detection among FSWs in Changshou City]. Shanghai Journal of Preventive Medicine 2011;**23**(8):377-79

183. Qin CZ, Yin J, Liu.Y., et al. [Analysis on AIDS Infection and Influencing Factors Among Female Sexual Workers in Jiangyen City]. Acta Universitatis Medicinalis Nanjing(Natural Science) 2012;**32**(12):1784-86+89

184. Wu J, He JG. [Survey on AIDS-related knowledge and behavior among prostitutes in Jinghu district Wuhu city,2011]. Anhui Journal of Preventive Medicine 2012;**18**(3):175-78

185. Sun BJ. [Survey on AIDS-related Knowledge, Behavior and HIV-infection Among Commercial Sex Workers in Shizhong District,Zaozhuang City,2011]. Preventive Medicine Tribune 2012;**18**(9):654-56

186. Yang YH, Gong CT, Wang ZQ, et al. [Analysis of national sentinel monitoring of AIDS infection in Quanzhou City in 2011]. Strait Journal of Preventive Medicine 2012;**18**(6):32-33

187. Chen YH. [Analysis of syphilis and HIV test results in 400 female sex workers in Rugao city]. Chinese Journal of Health Laboratory Technology 2012;**22**(4):892-93

188. Xu JS, Liu XY, Fu GF, et al. [Syphilis and HIV Infection Status Among Female Sex Workers in Jiangsu Province]. The Chinese Journal of Dermatovenereology 2012;**26**(6):513-15

189. Pan GL, Jin Y, Dong XJ. [Status of STDs Epedemic Among 617 Commercial Sexual Workers]. Zhejiang Journal of Preventive Medicine 2012;**24**(2):72-73

190. Tao SF, Zheng YJ. [Analysis of the characteristics of sexual behavior and syphilis infection among high risk population in Zongyang county,Anhui province]. Anhui Journal of Preventive Medicine 2012;**18**(3):195-97

191. Wu J, Shen Y. [Evaluation of AIDS/ STD risk behavior intervention among entertainment service workers]. Chin J Dis Control Prev 2010;**14**(9):914-16

192. YuQing L. [Study on syphilis prevalence of high risk group in Huai Rou district of Beijing, 2005]. Occupation and Health 2007;**23**(16):2

193. Liu Y, Yu M, Wan B, et al. [Epidemiological characteristics, sexually transmitted disease and HIV/AIDS status among 403 femal sex workers in Chaoyang district, Beijing]. Chin J Drug Depend 2006;**15**(5):401-04

194. Bai JM, Shi WY, Zhu LY, et al. [Study on behaviours and related diseases among 114 female sex workers in Fengtai District in Beijing]. Chinese Journal of AIDS and STD 2006;**12**(3):264

195. Xia Jianhui GY, Zhen Min Na, Liu Zhongquan, Zhu Xiaoke. [Analysis on syphilis epidemics in Tian Jing from 2006 to 2008]. China Tropical Medicine 2010;**v.10**(09):1081-82

196. Guo JM. [Analysis on results of STD and AIDS surveillance during 2006 in Huairou of Beijing]. Occupation and Health 2008;**24**(14):1415-16

197. Liu L, Liu M, Lu H, et al. [Analysis of HIV/AIDS related risk behaviors among female sex workers at entertainment establishments in two districts of Beijing]. Chin J AIDS STD 2007;**13**(6):532-35

198. AO X, Han Q. [Survey of AIDS-related knowledge, behaviors and infection HIV and syphilis among 105 sex workers]. Disease Surveillance 2008;**23**(11):714-16

199. Dong XY, Zhou N, Guo Y, et al. [Analysis of HIV/AIDS Sentinel Surveillance among female sex workers and male clients in Tianjin City, 2007]. South China Journal of Preventive Medicine 2009;**35**(2):40-41

200. Jiang DK, Zhang YY, Zhou L, et al. [The Analyses and Investigtion Reports of The Surveilance Program among The Population at High Risk for AIDS]. Journal of Medical Pest Control 2010;**26**(09):795-96, 99

201. Xu YJ, Wang SP, Xue ZD, et al. [An investigation of awareness rate on HIV/AIDS-related knowledge and risky sexual behaviours of unlicensed prostitutes in Shanxi province in 2009]. Chinese Remedies & Clinics 2011;**11**(3):304-06

202. Li M, Li M, Yu JP, et al. [Investigation and analysis on AIDS-related knowledge and behaviors among female sex workers in Xicheng district of Beijing from 2009-2011]. Occupation and Health 2012;**28**(22):2792-93, 95

203. Feng N. [Analysis of Survelliance monitoring of FSWs in Datong City in 2010]. Medical Information 2011;**24**(8):5028-29

204. Li BY, Zhao XH, Wang GR. [Recognition of AIDS among unlicensed prostitutes in Jinnan district of Tianjin]. Occupation and Health 2012;**28**(4):465-66

205. Li F, Ma SB, Liu HX. [Analysis of AIDS sentinel surveillance of Changping District in Beijing,2011]. Chinese Journal of Health Education 2012;**28**(6):469-71+75

206. Liu ZJ, Jiang L, Wang CB, et al. [Sentinel surveillance of female sex workers in Luannan County in 2011]. Journal of Capital Medical University 2012;**33**(5):621-24

207. Zhang P, Zhang J. [Survey of HIV/AIDS related knowledge and behavior among FSWs]. China Health Industry 2012;**9**(8):68-69

208. Cui YZ, Zhu L, Yuan LL, et al. [AIDS knowledge, behavior and HIV/syphilis infection survey among FSW in Harin from 2006 to 2009]. Chinese Journal of AIDS & STD 2011;**17**(3):351-52

209. Qi G, Zhang X, Li X, et al. [Investigation on HIV/AIDS-related knowledge and behaviors of commerical sex workers in Changchun entertainment establishments]. Chinese primary health care 2008;**22**(12):66-68

210. Zheng J, Yin Y, Zhao WS. [Effect Evaluation on the intervention on HIV-related knowledge and behaviors among female sex workers in Anshan City] Chinese Medical Journal of Metallurgical Industry 2012;**29**(2):201-02

211. Zhou D, Zhou JL, Jiang FX, et al. [An analysis of HIV/AIDS sentinel surveillance to high risk populations in Liaoning province in 2011]. Chinese Journal of AIDS & STD 2012;**18**(10):648-50+56

212. Yu QiaoLing YY, Liu Rong, Chun ZhiMing, Qu Li, Zhang Juan. [Survey on HBV, syphilis and HIV infection rate among high risk group in Dun Huang]. Strait J Prev Med 2007:1

213. Zhang M, Rui BL, Xue Q, et al. [Study on HIV and Syphilis infection among female sex workers in Urumqi of Xinjiang]. China Preventive Medicine 2006;**7**(03):216-17

214. Wei XW, Wang H, Yang JZ, et al. Study of the Interfering Effect on the STD/AIDS Incidence Rate Among the Service Maids. Chin J Derm Venereol 2004;**18**(12):732-34

215. Ji C, Ren X, Shen J, et al. [Analysis of STD and AIDS of commercial sex workers in entertainment places in Gaoping city from 2004 to 2006]. Prev Med Trib 2009;**15**(10):918-19

216. Lin Bing ZK, Meng Wei, Guo Jun, A Simengguli, Hu Jun. [Analysis of AIDS sentinel surveilance among female sex workers in Karamay from 2004 to 2008] Chin J Public Health 2009;**25**(1)

217. Song Y, Wang Q. [A study of HIV infection status among entertainment-based female sex workers in Alar City, Xinjiang, 2004]. Endemic Diseases Bulletin 2005;**20**(02):45-46

218. Zhang L, Li Y, Chen JJ. [Analysis on HIV knowledge and awareness among female sex workers in entertainment venues in Lanzhou City of Gansu Province]. Health Vovational Education 2007;**25**(20):95-96

219. Ma JX, Fan XM, Su YZ, et al. [Analysis of the changing trend of AIDS high-risk behaviors among CSWs in Baiyin City]. Health Vocational Education 2012;**30**(6):98-99

220. Lin B, Luo M, Wang S, et al. [Effectiveness evaluation of AIDS related integrated interventions among female sex workers in entertainment settings in Karamay city]. Chin J AIDS STD 2009;**v.15;No.82**(01):41-43

221. Liang S, Zhang S. [Effectiveness evaluation of HIV interventions among female sex workers in Huinong district, Guangxi province]. J Med Pest Control 2010;**26**(12):1155-56

222. Zhao AL. [Epidemiology of syphilis and HIV among FSWs in Lanzhou city]. Gansu Science and Technology 2011;**27**(4):131-32

223. Ma L, Yang YH. [Report on Final Evaluation of Condom use Intervention to FSWs in Hutubi county in Changji Hui autonomous prefecture]. Bulletin of Disease Control and Prevention 2011;**26**(6):28-30

224. Xu Y, Wang S, Xue Z, et al. [Study on knowledge of HIV infection and unsafe sexual behaviour among female sex workers in Shan Xi, 2009]. Chinese Remedies & Clinics 2011;**v.11**(03):304-06

225. Hu XQ, Yan CY, Yang PR, et al. [Analysis of monitoring AIDS high-risk populations in 2011]. Journal of Preventive Medicine of Chinese People's Liberation Army 2011;**29**(6):437-38

226. Wu R, Xue SF, Yao XW. [An investigation of HIV/AIDS-related risk information among 412 female sex workers]. Chinese Journal of AIDS & STD 2012;**18**(11):793, 96

227. Tao LD, Qi YJ, Wei HW, et al. [Epidemiology of HIV and Syphilis Among Female Sex Workers in Chenguan County, Gansu Province]. Chinese Primary Health Care 2011;**25**(9):65-66

228. Guo H, SheN MX, Ma Q, et al. [The HIV Risk Behavior Surveillance Analysis for 820 Slip Women in Longnan Municiple]. Chinese Primary Health Care 2012;**26**(4):65-66

229. Xu G. [Epidemiology of Syphilis Among Female Sex Workers from 1998 -2005]. Guide of China Medicine 2012;**10**(9):491-92

230. Wang Lirong CJ, Chen Yi, Li Shenghua. [Survey on syphilis prevalence situtation in Zhan Jiang]. Chinese Journal of Health Laboratory Technology 2004(04):483-84

231. Zhong N, Lu Y, Wang F. [Analysis of the result of Syphilis Infection in 3939 Prostitutes]. China Tropical Medicine 2002;**2**(1):100, 04

232. Pei D, Yang B, Lai S, et al. [High risk behavior investigation and syphilis infection survelliance among female sex workers and drug users in Hainan]. Chin J STD/AIDS Prev Cont 2002;**8**(3):160-61

233. Liang Q. [Analysis of syphilis prevalence among detained female sex workers in Liu Zhou, Guang Xi 2002]. Journal of Youjiang Medical College for Nationalities 2004(02):257-58

234. Zheng W, Zhong N, Zhu H, et al. [Analysis of the Monitoring Results of 1144 Unlicensed Prostitutes for Infection of Hepatitis B Virus( HBV) , HumamIm2 munodeficiency Virus( HIV) and Syphilis]. Chin J Derm Venereol 2009;**v.23;No.v.23**(08):502-03

235. Wang X, Zhang Q, Liu Y, et al. [Surveillance reports on AIDS and Syphilis of 103 prostitute women in Dongguan city]. Disease Surveillance 2005;**20**(03):121-22

236. Wu Y, Zhou Y, Liu R, et al. [Analysis of results in detection of syphilis infection in 281 sex workers]. China Tropical Medicine 2005;**5**(1):170-71

237. Wu X. [An analysis of sexual behaviours among 465 female sex workers in Xinyang City]. Journal of Medical Forum 2005;**26**(17):5-7

238. Yang BF, Xu J, Yao ZZ, et al. [An analysis of the behavior characteristics and the infection of HIV and syphilis among the commercial female sex workers]. Chinese Journal of Disease Control & Prevention 2006;**2006**(4):406-08

239. Li W, Lin P, Mai R, et al. [Effectiveness evaluation of HIV interventions among female sex workers in Yangjiang prefecture, China]. South China J PrevMed 2008;**34**(02):31-32

240. Li W, Li Y, Mai R, et al. [Survey of STD and AIDS Knowledge and High-risk Behavior of Female Sexual Workers in Urban Area of Yangjiang City]. China tropical medicine 2007(10):1921-22+40

241. XiaoXia W, BoFen C, Xiaojun F. [Sentinel report of HIV/syphilis from detained sex workers in Dong Guan, Guang Dong]. Disease Surveillance 2008(08):490-92

242. Xu YF, Zhou FH, Mo SJ, et al. [Surveillance of Commercial Sex Workers in Nanning, 200 -2008]. Journal of Preventive Medicine Information 2009;**25**(8):615-17

243. Tan WW, Liu HY, Liu FX, et al. [Sentinel surveillance of syphilis in female sex workers in Nanning city, 2007- 2009]. Disease Surveillance 2011;**26**(2):106-09

244. Wang Q, Yang P, Gong X, et al. [Syphilis prevalence and high risk behaviors among female sex workers in different settings]. Chin J AIDS STD 2009;**15**(04):398-401

245. Tan WW, Liu HY, Liu FX, et al. [Sentinel surveillance of syphilis in female sex workers in Nanning city, 2007-2009]. Disease Surveillance 2011;**26**(2):106-09

246. Qin Y, Wang X, Shi X, et al. [Analysis of HIV and Syphilis infection among sex workers, drug abusers and container drivers in Shenzhen]. Journal of Tropical Medicine 2010;**10**(07):889-91

247. Peng H, Yang L, Zhang S, et al. [HIV knowledge, attitude and behavioural survey among 60 syphilis-infected street female sex workers]. Chin J AIDS STD 2008;**14**(6):628-29

248. Yang P, Wang QQ, Peng H, et al. [A survey of syphilis and HIV infection in medium-low-income female sex workers]. China Journal of Leprosy and Skin Diseases 2009;**25**(3):174-76

249. Tan W, Zhou H, Liu H, et al. [Comprehensive HIV surveillance results analysis for commercial sex workers in Nanning City, 2007]. Guangxi Medical Journal 2008;**30**(11):1727-28

250. Lin M, Jian H, Liang F, et al. [STD/AIDS Screening Rersults and Related Knowledge and Behavior of Female Sex Workers in Western Guangxi]. Journal of Nursing Science 2008;**23**(17):27-29

251. Ba iY. [Investigation on AIDS related knowledge, behaviors and infection among commercial female sex workers in Liuzhou city in 2008]. Prev Med Trib 2009;**15**(12):1224-26

252. Lin RL, Quan XB, Ye RG, et al. [Investigation on core indicator of behavioral surveillance among female sex workers in Yulin,2009-2011]. China Tropical Medicine 2012;**12**(6):700-02+24

253. Ren XQ, Yang LG, Wang XH, et al. [Investigation of syphilis prevalence among high risk people with STD in Guangdong province]. Chinese Journal of AIDS & STD 2011;**17**(4):436-38

254. Xiang H. [Analysis of the HIV-related praxiology among FSWs from entertainment venues in Laifeng County of Hubei Province at 2009]. Journal of Mathematical Medicine 2011;**24**(2):199-201

255. Wu ZZ, Deng WJ, Zhu.X., et al. [Study of Commercial Sex Workers Positive of Syphilis Serum and its Related Factors]. Qingdao Medical Journal 2011;**43**(5):321-24

256. Shi JX, Yan CX. [Investigation on intervention results of female sexual workers in Ping'shan district in Shenzhen]. Chinese Journal of Pest Control 2012;**28**(6):682-83+86

257. Li J, Chen X-S, Merli MG, et al. Systematic Differences in Risk Behaviors and Syphilis Prevalence Across Types of Female Sex Workers: A Preliminary Study in Liuzhou, China. Sexually Transmitted Diseases 2012;**39**(3):195-200 10.1097/OLQ.0b013e31823d2e2a

258. Weir SS, Merli MG, Li J, et al. A comparison of respondent-driven and venue-based sampling of female sex workers in Liuzhou, China. Sex Transm Infect 2012;**88 Suppl 2**:i95-101 doi: 10.1136/sextrans-2012-050638[published Online First: Epub Date]|.

259. Li J, Chen XS, Merli MG, et al. Systematic differences in risk behaviors and syphilis prevalence across types of female sex workers: a preliminary study in Liuzhou, China. Sexually transmitted diseases 2012;**39**(3):195-200 doi: 10.1097/OLQ.0b013e31823d2e2a[published Online First: Epub Date]|.

260. Zhou Y, Chen W, Zhou MR. [Evaluation on AIDS intervention programme among female sex workers in Guilin,2009-1010]. Disease Surveillance 2012;**27**(1):35-39

261. Jiang N. [Surveillance of risk behawiors facilitating among commercial sex works and analysis of HIV,Syphilis,HCV and HBV infection]. Journal of Medical Forum 2012;**33**(4):81-82

262. Huang KZ, Chen XH, Li WJ, et al. [Survey of STD and AIDS knowledge and high risk behavior of female sexual workers in Yangjiang City]. South China Journal of Preventive Medicine 2010;**36**(04):41-42

263. Chen CF, Zhao Y, Xiao HT, et al. [Analysis of HIV/AIDS sentinel surveillance among commercial sex workers in Dangyang City in 2010]. Journal of Public Health and Preventive Medicine 2011;**22**(3):73-74

264. Liu XF, lV ML, Li ZM. [Syphilis infection among different population groups in Zhuhai, Guangdong province]. Chinese Journal of AIDS & STD 2012;**18**(1):50-51+53

265. Xiong CS, Mao LF, Peng J, et al. [Analysis of HIV/AIDS Knowledge and related Behavior within 400 Female Sex Workers]. Journal of Public Health and Preventive Medicine 2012;**23**(3):86-87

266. Chen ZB, Pan XM, Chen ZT, et al. [A study of HIV/AIDS-related knowlege and behaviours on female sex workers at different venues in Lianzhou city of Guangdong province]. South China Journal of Preventive Medicine 2011;**37**(2):34-36

267. Li Y, Wu D, Wang SY. [Analysis of Sentinel Surveillence on FSWs in Xiaonan District of Hubei Province in 2011]. Today Nurse 2012(4):132-33

268. Tang MJ, Zhong FH, Liu JJ, et al. [Analysis of AIDS Sentinel Surveillance in Yulin city in 2011]. Journal of Applied Preventive Medicine 2012;**18**(1):40-42

269. Tang J, Zhang ZK, Zhou Y, et al. [Analysis of sentinel monitoring of AIDS among high risk population in Guilin,2011]. Chinese Journal of AIDS & STD 2012;**18**(8):533-36

270. Ke XZ, Xiong F, Xie RQ, et al. [Analysis of sentinel monitoring of HIV, syphilis and HCV in Huangshi City in 2011]. Journal of Public Health and Preventive Medicine 2012;**23**(5):80-81

271. Quan XB, Lin RL, Huang ZN, et al. [Study on HIV /TP /HCV infection and condom use of prostitute in Yulin city]. Chinese Journal of Health Laboratory Technology 2012;**22**(7):1687-89

272. Guo HJ, Feng D, Zhou CX, et al. [Analysis of results of screening and monitoring of HIV/AIDS/STDs among FSWs in entertainment venues by using a real name system in a county of Guizhou]. Chinese Journal of AIDS & STD 2012;**18**(2):104-06

273. Lei Z, Du M, Wang z, et al. [Analysis of serological and behavioral survey results for HIV and TP in 259 female sex workers (FSW) from communities in 2004] Disease Surveillance 2005;**20**(09):30-32

274. Xushi M, Qiantian X, Pengchuan L, et al. [Survailence outcome of HIV/Syphilis prevalence in Chong Qing 2004 ]. Modern Practical Medicine 2006(05):833-34

275. Choi SYP. State Control, Female Prostitution and HIV Prevention in China. The China Quarterly 2011;**205**:96-114 doi: doi:10.1017/S0305741010001414[published Online First: Epub Date]|.

276. Li D, Yuan F, Hu S, et al. [High risk behaviors and HIV/STI prevalence among female sex workers in different settings]. Chin J AIDS STD 2007;**13**(03):210-13

277. Zheng QC, Gan YG, Li YM, et al. [Evaluation of the Effect of Behaviors Interference Among Female Sexual Workers Serving in a District of Chongqing]. Modern Preventive Medicine 2012;**39**(6):1399-401+404

278. De QingZuoGa CD. [Result analysis on syphilis and HIV prevalence of different population in La Sa, Tibet 2007- 2008]. Practical Preventive Medicine 2009;**v.16**(05):1446-47

279. Li W. [HIV sentinel surveillance and analysis among female sex workers in Jianshui county, China]. Soft Science of Health 2009;**23**(06):711-13

280. Chen C. [Study on HCV, syphilis and HIV infection among female sex workers in middle-low class entertainment venues]. Medical information 2010;**05**(10):2076

281. Zhang H, Li HL, Wang C, et al. [Investigation on HIV/AIDS know ledge and related behavior of 1301 female commercial sex workers]. Soft Science of Health 2012;**26**(1):47-49

282. Xu L, Shi F, Wang L. [Survey on knowledge of STI/AIDS and sexual behaviour among female sex workers in entertainment venue in Chang Shu]. Jiangsu J Prev Med 2008:79-81

283. Wu Y, Zhang J, Hua G, et al. [Screening Analysis and Interventional Study of Trichomonal Vaginitis in Female Commercial Sex Workers]. Chinese Journal of AIDS & STD 2001;**7**(6):366-67

284. Yang HW, Zhu XY, Sun HY, et al. [Investigation on sexual behaviour, health-seeking behaviour and gynecological examination among female sex workers in a city]. Journal of Preventive Medicine Information 2003;**19**(z1):42-43

285. Liu S, An B. [Survey on AIDS related knowledge and behavior in sex workers at recreation places]. Chin J Public Health 2008;**24**(01):18-19

286. Che ZM, Luo W. [The use of an information management system in HIV and STD management amongst female sex workers at entertainment establishments] Journal of Dermatology and Venereology 2008;**115**(2):38-40

287. Wang X, Gu D, Lou B, et al. Hospital-based prevalence of high-risk cervical HPV types infecting the general population and female sex workers in Huzhou, China. Int J Gynaecol Obstet 2013;**120**(1):37-41 doi: 10.1016/j.ijgo.2012.07.019[published Online First: Epub Date]|.

288. YE Qiu-ru SQ, Jiang-hua L, Shu L. [Comparison of Infection Rate and Different Genotypes of the Cervical HPV between Female Sexual Workers and General People]. JOURNAL OF CHINA MEDICAL UNIVERSITY 2009;**38**(12):930-32

289. Li HM, Liang GJ, Yin YP, et al. Prevalence and genotype distribution of human papillomavirus infection among female sex workers in Guangxi, China: implications for interventions. Journal of medical virology 2012;**84**(5):798-803 doi: 10.1002/jmv.23264[published Online First: Epub Date]|.

290. Wang Y, Li X, Zhang B, et al. [Factors associated with HIV/AIDS STD among young female sex workers in Qingdao]. China J Lepr Skin Dis 2008;**24**(06):424-26

291. Yang Y, Yao J, Gao M, et al. Herpes simplex virus type 2 infection among female sex workers in Shanghai, China. AIDS care 2011;**23 Suppl 1**:37-44 doi: 10.1080/09540121.2011.555740[published Online First: Epub Date]|.

292. Dai X, Tang K, Huang X, et al. [Investigation and analysis on sexually transmitted infection among different females group in Guangzhou]. Journal of Tropical Medicine 2009;**9**(07):792-95

293. Ngo TD, Laeyendecker O, Li C, et al. Herpes simplex virus type 2 infection among commercial sex workers in Kunming, Yunnan Province, China. Int J STD AIDS 2008;**19**(10):694-7

294. Liu HB, Nie K, Li PS. [Analysis of the HBV and HCV infection among different populations in Qingyunpu District of Nanchang City]. Journal of North Pharmacy 2012;**9**(1):87

295. Zhao LH, Zhang SR, Wang WR. [Prevalence of HBV, HCV and HIV infection among 361 prostitutes]. Journal of Practical Medical Techniques 2006;**13**(10):1762-63

296. Yin FL, Shi GZ, Zhang Y, et al. [Investigation of HIV/AIDS infection, knowledge and behavior among female sex workers in Jiading District of Shanghai City]. Shanghai Journal of Preventive Medicine 2009;**21**(10):484-85

297. Fu ZW, Sun LY, Zeng XX. [Analysis on results of Hepatitis B serological survey in special populations in Hainan Province]. Modern Preventive Medicine 2012;**39**(23):6285-87

298. YaQun Q. [Analysis on hepatitis infection situation among key population]. China Tropical Medicine 2004;**4**(4):2

299. Ping G. [Logistic regression on sexual transmitted infections and hepatitis virus infection]. Anthology of Medicine 2005;**24**(2):3

300. Yan LM, Wang SY, Li ZH, et al. [STD/AIDS knowledge, attitude, behavior and infection status among 182 prostitutes in Xiaogan urban area]. Journal of Public Health and Preventive Medicine 2007;**18**(1):67-68

301. Chen R. [Investigation on HBV and HCV infection among female sex workers, injecting drug users and sexually transmitted patients in Bao An, Shen Zhen]. OCCUPATION AND HEALTH 2004;**20**(09):84-85

302. Zhang Qian WY, Li Ping. [Study of HIV/TP/HCV infection of 394 prostitutes in Wuhu City]. Chinese Journal of Health Laboratory Technology 2008;**18**(9):1865-68

303. Yu Wenxiang QY, Wang Haiyan, Sun Kaiyou. [Report of AIDS/STD prevalence from sentinel point in Yan Cheng, 2005]. Jiangsu Prev Med 2006(04):16-17

304. Jin T, Yan J, Ma Q, et al. [Survey on status Quo of Knowledge about and behaviors of AIDS prevention and control of unlicensed prostitutes in the entertainment establishments in the city of Lishui ]. Disease Surveillance 2005;**20**(12):621-24

305. Jin Y, Yao Y, Ye D, et al. [Investigation and analysis on condom use status female commercial sex workers ]. Chin J Dis Control Prev 2009;**13**(01):20-22

306. Liao MZ, Liu XZ, Kang DM, et al. [Analysis on the HIV/AIDS Surveillance Data in Shandong Province in 2009]. Preventive Medicine Tribune 2010;**16**(5):398-400, 03

307. Sun XQ, Tang GX, Mao TS, et al. [2010 baseline survey among commercial sex workers of Taihe County]. China Modern Medicine 2011;**18**(28):151-53

308. Hao XG, Wang W. [Sentinel surveillance of HIV/AIDS in female sex workers in Kecheng district of Quzhou city,Zhejiang province ,2011]. Disease Surveillance 2012;**27**(4):300-03

309. Wang DL, Zhu YS, Gu CY, et al. [Analysis of sentinel monitoring of CSWs in Zhonglou district of Changzhou city]. Jiangsu Journal of Preventive Medicine 2012;**23**(3):47-48

310. Wang YP, Wang F, Shi DY, et al. [Analysis of sentinel monitoring on illicit prostitutes in Jurong City]. Jiangsu Health Care 2012;**14**(6):7-8

311. Qiu ZH, Dong ZQ, Jin MH, et al. [Sentinel surveillance of AIDS among female sex workers in Huzhou,Zhejiang, 2011]. Disease Surveillance 2012;**27**(4):291-93

312. Ye ZM, Wang DY, Zhang HM, et al. [Survey on HIV/AIDS -related knowledge and behaviors among FSWs]. Zhejiang Journal of Preventive Medicine 2012;**24**(11):74-76

313. Zhu HW, Huang SP, Zhu RH, et al. [Analysis of AIDS Sentinel Surveillance in Nanchang County in 2011]. Chinese Community Doctors 2012;**14**(32):338

314. Kang YF, Yu AQ, Yang HF, et al. [Analysis of Detection Data of HIV and HCV Infection in Different Sentinel of Yangquan City, 2008-2010]. Preventive Medicine Tribune 2011;**17**(3):276-77

315. Liu XS, Di HY, Wang YY, et al. [Investigation on venereal disease infection and medical treatment among unlicensed prostitutes in Shijiazhuang]. Journal of Medical Pest Control 2012;**28**(11):1272-73

316. Xian XJ. [Analysis of sentinel monitoring on HIV/AIDS in Nongan County of Jilin Province]. Youthful days 2011;**462**(22):261

317. Zhao GD, Zhong L, Li YY. [Analysis of AIDS sentinel surveillance among illicit prostitutes in Shangluo City in 2010]. Journal of Hebei United University(Health Sciences) 2011;**13**(4):469-70

318. Zhang MN, Zhang ZH, Huang L, et al. [An investigation of sexually transmitted infection and related behavioral feature on unlicensed prostitutes in Shanxi province]. Chinese Remedies & Clinics 2011;**11**(9):1051-52

319. Liu C, Xu J, Zhou W, et al. [Baseline investigation on three kinds of high risk population in Wuhan,China Bill& Melinda Gates Foundation AIDS program]. Journal of Public Health and Preventive Medicine 2011;**22**(2):15-18

320. Luo JH, Jin WD, Li GB, et al. [Analysis of Survelliance monitoring of Illicit Prostitutes in Shaodong County, Shaoyang County of Hunan Province in 2009] Practical Preventive Medicine 2011;**18**(1):166-67

321. Nie ZQ, Lin P, Li Y, et al. [Surveillance of AIDS high-risk people in Guangdong province, 2009]. Journal of Tropical Medicine 2011;**11**(1):29-31+45

322. Zhang L. [Invesitigation on HIV-related Knowledge and Behaviors among Illicit Prostitutes in Xinyang City in 2009]. Henan Journal of preventive Medicine 2011;**22**(2):109-10

323. Zhou Jin Lan DKM. [Survailence on HIV in Bei Hu district, 2009]. Practical Preventive Medicine 2010;**v.17**(02):392-93+90

324. Wen X. [An analysis on condom using behavior and influencing factors among female sex workers(FSW) in Guilin city]. Jian Kang Tian Di 2010;**4**(9):2

325. Nie Zhi Qiang LP, Li Yan, Wang Ye. [Survilance analysis on HIV among high risk group in Guang Dong, 2009]. China Tropical Medicine 2011;**v.11**(01):29-31+45

326. Yang JY, Liu Y, Yan YB, et al. [Analysis of HCV infection among populations]. Contemporary Medicine 2011;**17**(18):159-60

327. Gui Q, Zeng LG, Wang LY, et al. [Analysis of sentinel surveillance of FSWs in Wuling District of Changde City in 2010]. Chinese Journal of AIDS & STD 2012;**18**(10):701-02

328. Bai Y, Zhang JP, Ouyang Y. [Analysis on monitoring results of AIDS among female sexual workers in Liuzhou City in 2010]. Chinese Journal of Pest Control 2012;**28**(9):964-66

329. Wang GH, Fu HP, Dong DY. [Investigation of HIV-related bahavior and infection of FSWs]. Zhejiang Journal of Preventive Medicine 2011;**23**(10):35-36

330. Yan WZ, Zheng KQ, Feng DL, et al. [Analysis on STD/AIDS epidemic among female sex workers in Jinghong City of Yunnan Province in 2009] Journal of Dermatology and Venereology 2010;**32**(4):48-50

331. Li YK, Feng L, Deng B, et al. [Effect of Intervention to Commercial Sex Workers in Comprehensive Prevention Demonstrative Area of Sichuan Province]. Journal of Preventive Medicine Information 2012;**28**(7):528-31

332. Wang Wan Wei YSL, Lin Guang Li. [Surveilance results on HIV epidemic among high risk group in Nei Jiang, 2009]. Journal of Preventive Medicine Information 2010;**v.26**(04):309-11

333. He B, Nong LP, Bi SZ, et al. [Analysis on Effectiveness of Comprehensive Interventions for HIV/AIDS Among Entertainment Establishments in Pingxiang]. Practical Preventive Medicine 2012;**19**(3):329-32

334. Zhang H, Kong SW, Wang CY, et al. [HCV Test Results of 4 Classes Population in Yibin]. Parasitoses and Infectious Diseases 2011;**9**(4):233-34

335. Zhou CX, Pan ZP, Chen ZY, et al. [Results analysis of AIDS sentinel surveillance in Zunyi city, 2010]. Jiangsu Journal of Preventive Medicine 2011;**22**(3):11-13

336. Wangmo T, Zhou YL, Zhang XH, et al. [Analysis of results of sentinel monitoring on FSWs in Lhasa City]. Jiangsu Journal of Preventive Medicine 2012;**23**(6):41-42

337. Ouyang H. [Analysis of STDs infection and HIV-related knowledge among commercial sex workers in Hejiang in 2011]. Journal of Occupational Health and Damage 2012;**27**(2):126-27

338. Luo J. [Analysis of the results from HIV Surveillance of unlicensed prostitutes]. Disease Surveillance 2005;**20**(8):409-12

339. Chen XS, Yin YP, Liang GJ, et al. Sexually transmitted infections among female sex workers in Yunnan, China. AIDS patient care and STDs 2005;**19**(12):853-60 doi: 10.1089/apc.2005.19.853[published Online First: Epub Date]|.
