# Supplementary figures and images for: Sustained high prevalence of viral hepatitis and sexually transmissible infections among female sex workers in China: a systematic review and meta-analysis
Source: BMC Infect Dis. 2016 Jan 5;16:2. doi: 10.1186/s12879-015-1322-0 (PMC4702370; doi:10.1186/s12879-015-1322-0)

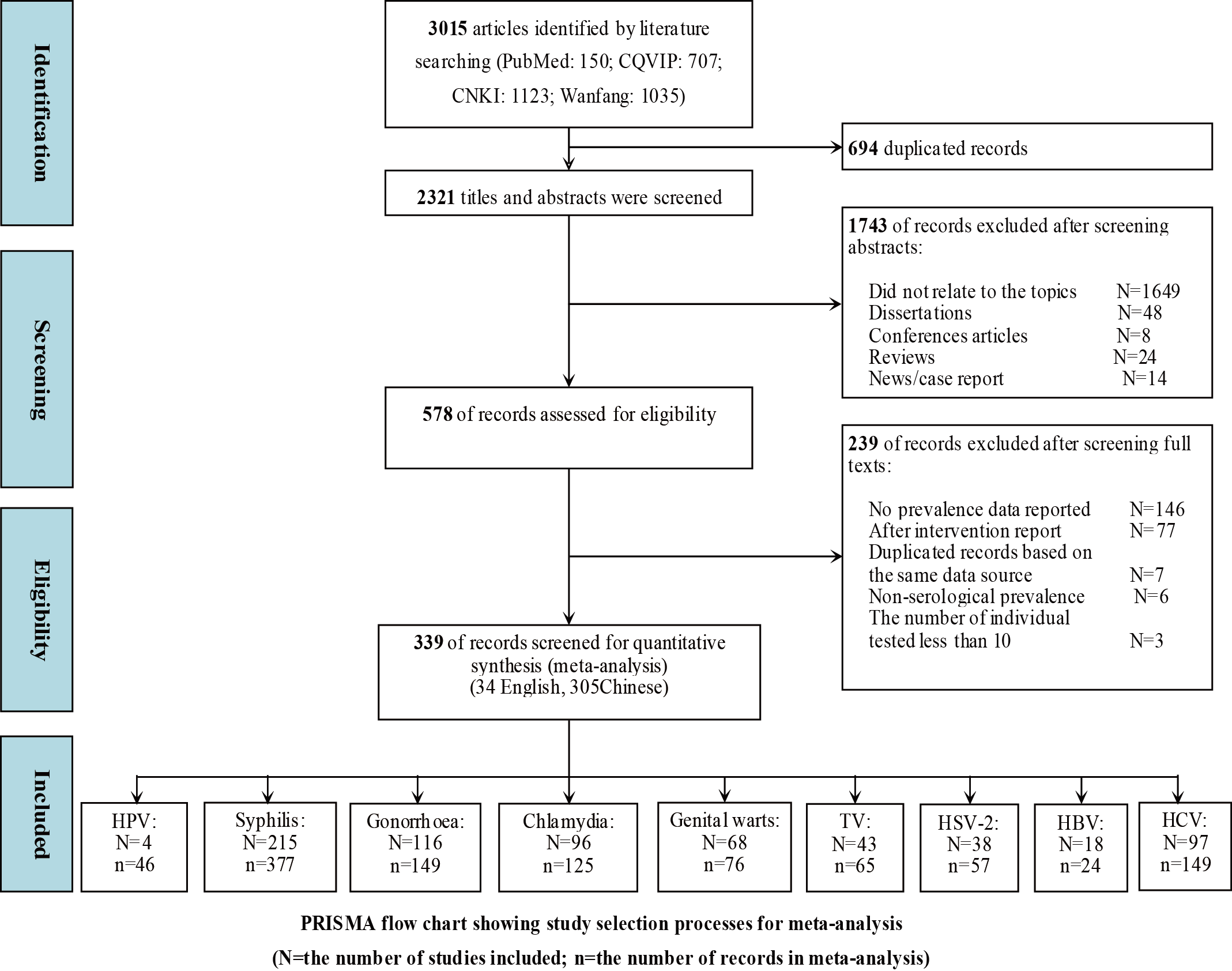

Supplement: Supplementary file 2 — PRISMA flow chart for selection of studies. (PNG 159 kb) [file 12879_2015_1322_MOESM2_ESM.png]
